# Oxidative stress-responsive cell wall remodeling depends on phosphate in *Candida albicans*

**DOI:** 10.1038/s41467-026-77594-x

**Published:** 2026-09-10

**Authors:** Anand Jacob, Wanjun Qi, Jayasubba Reddy Yarava, Murtaza Barkarar, Alicia Withrow, Aswath Karai, Josh V. Vermaas, Julia R. Köhler, Tuo Wang

**Affiliations:** 1 Department of Chemistry, Michigan State University, East Lansing, MI USA; 2 Division of Infectious Diseases, Boston Children’s Hospital/Harvard Medical School, Boston, MA USA; 3 MSU-DOE Plant Research Laboratory, East Lansing, MI USA; 4 Department of Biochemistry and Molecular Biology, Michigan State University, East Lansing, MI USA; 5 Center for Advanced Microscopy, Michigan State University, East Lansing, MI USA

**Keywords:** Biopolymers in vivo, Solid-state NMR, Fungal biology

## Abstract

The growing number of patients susceptible to invasive *Candida albicans* infections has intensified the need for effective antifungal targets in pathways essential for fungal growth and pathogenesis. Among these pathways, phosphate homeostasis has emerged as a significant determinant of virulence, yet how phosphate availability shapes cell wall structure in response to host-derived oxidative stress remains unclear. During commensal growth, *C. albicans* cells typically enjoy phosphate repletion and a neutral redox environment. Transitioning to invade host tissues, they simultaneously experience phosphate deprivation and intense extrinsic oxidative stress. Here, we employ solid-state NMR to render details of cell wall remodeling in response to oxidative stress, in its dependence on phosphate. Phosphate deprived cells remodel the rigid wall core and reduce hydration and polymer mobility in the absence of oxidative stress. During hydrogen peroxide exposure, highly mobile outer polysaccharides are primary interactors. In wildtype cells, some of these polymers are recruited into the rigid core, reinforcing the wall scaffold, whereas phosphate transport mutants fail to undergo this remodeling. These findings establish phosphate acquisition as a component of oxidative defense and link nutrient sensing and -availability to the mechanical resilience of the fungal cell wall, revealing an architectural vulnerability with relevance for antifungal development.

## Abstract (web summary)

*Candida albicans*, the leading cause of invasive candidiasis, requires phosphate acquisition to remodel its cell wall and withstand oxidative stress, revealing phosphate homeostasis as a structural determinant of fungal resilience.

## Introduction

An important worldwide challenge is the growing number of immunocompromised individuals at risk for invasive candidiasis, driven by therapeutic progress in oncology, rheumatology, gastroenterology, neonatology, and transplantation medicine^1–3^. Despite the increasing clinical need, the current antifungal armamentarium, comprising echinocandins, polyenes, azoles, and the pyrimidine analog 5-flucytosine, is constrained by substantial limitations, including toxicity, pharmacologic interactions, inadequate tissue penetration, and formulations restricted to intravenous or enteral delivery^4,5^. Given the wide range of comorbidities and clinical conditions encountered among patients with invasive candidiasis, no single antifungal agent is suitable for all patient profiles^6^. Compounding these challenges, *C. albicans* strains and other *Candida* species resistant to antifungal agents, particularly azoles and echinocandins, emerge during prolonged treatment courses, and multidrug-resistant strains are associated with increased mortality and healthcare costs^4,5,7–10^. Therefore, *C. albicans* has been categorized as a critical-priority fungal pathogen on the WHO fungal priority pathogens list^11,12^. Given the limited efficacy of existing antifungal therapies in complex clinical scenarios, identification and characterization of potential therapeutic targets is urgently needed.

To identify the opportunities for antifungal intervention, it is essential to focus on pathways indispensable for fungal growth and pathogenesis within the host environment, among which phosphate metabolism is particularly compelling. Phosphate is an essential macronutrient for all living organisms, supporting the synthesis of nucleic acids, phospholipids, and ATP, as well as functioning as a key regulator within numerous cellular signaling pathways^13,14^. At the same time, fungal phosphate homeostasis differs fundamentally from that of humans^15^.

*C. albicans* colonizes the gastrointestinal tract of an estimated 50-83% of adults, where it coexists with hundreds of bacterial species in the colon^16,17^. Commensal physiology of *C. albicans* is a distinct state with apparent specific characteristics like suppression of hyphal growth^18,19^. During episodes of disruption of the intestinal epithelial barrier and insufficient response of host neutrophils, the fungus switches its physiology from commensal to infectious, invading host tissues and bloodstream^20^. This transition exposes *C. albicans* to a dramatic change of external conditions, including increased oxygen tension and pH, as well as altered nutrient availability, including phosphate^21^. In addition, invading *C. albicans* cells are typically confronted by host innate immune cells, neutrophils and macrophages.

Human stool contains an average of 11 and up to 33 mM soluble phosphate, indicating that as an intestinal commensal, *C. albicans* experiences phosphate repletion^22^. In contrast, as it invades the tissues and bloodstream of the human host, the fungus experiences phosphate starvation: inorganic phosphate concentration of plasma (which comprises 85% of total plasma phosphate, with the other 15% consisting of organic compounds like glycerophosphocholine) is in the 0.8-1.6 mM range^23,24^. Additionally limiting access to host phosphate is the fact that in the bloodstream and in other extracellular fluid, 15% of inorganic phosphate is protein-bound and another fraction is insolubly complexed to cations like calcium and magnesium. Human body fluids and tissues are slightly alkaline at pH 7.35-7.45, further reducing phosphate accessibility for *C. albicans* whose principal inorganic phosphate transporter, Pho84, has an acidic optimum, a dilemma *C. albicans* shares with other fungal pathogens^25,26^. For these reasons, transcriptional phosphate starvation responses are induced during *C. albicans’* invasive growth in the host^27–29^.

*C. albicans* cells undergoing the transition from commensal to invasive physiology therefore must adapt from a phosphate replete to a phosphate depleted nutritional state. Among genes induced during phosphate deprivation, transcriptional studies have identified cell wall component and remodeling genes, including *MNN1, XOG1*, and *MNN22*^30^. These genes encode an α−1,3-mannosyltransferase, an exo-1,3-β-glucanase and an α−1,2-mannosyltransferase, respectively, and are 18-, 16- and 15-fold upregulated when wildtype cells lack phosphate^30^. β-mannosyltransferases *BMT3* and *BMT4* are 11- and 9-fold upregulated under these conditions, while chitinase *CHT1* and another putative β-mannosyltransferase, *RHD1*, are 4-fold upregulated. Strikingly, putative sensors of cell wall stress are also upregulated, specifically *PGA28* (24-fold up) and *WSC4* (3-fold up), in addition to the marked upregulation of remodeling enzymes, hinting that phosphate scarcity-induced cell wall alterations could predispose to cell wall stress. In fact, cells that lack the major inorganic phosphate transporter Pho84 are hypersensitive to cell wall stress^31^. In addition to Pho84, *C. albicans* encodes three other phosphate transporters that contribute to phosphate acquisition but have more limited physiological roles^25^.

During host tissue and bloodstream invasion, simultaneously with phosphate deprivation and the increased oxygen tension of normally oxygenated blood and tissues, *C. albicans* also encounters intense oxidative stress generated by innate immune cells, neutrophils and macrophages. During the respiratory burst, these phagocytes produce superoxide that is dismutated to hydrogen peroxide, H_2_O_2,_ by enzymes embedded in the cell wall on the fungal surface and in the cytoplasm^32,33^. *C. albicans* deploys an integrated network of antioxidant defenses, including superoxide dismutases, catalase, and the thioredoxin and glutathione systems, which together detoxify ROS and limit cellular damage^34–36^. The activity of these systems is coordinated by stress response signaling pathways such as the Hog1 MAPK pathway and the Cap1 transcriptional regulator^37–41^. In addition, the cell activates protein quality control mechanisms involving the proteasome and initiates DNA repair processes to maintain essential cellular function^42–44^.

Since the wall is the first organelle of the fungal cell to encounter host-derived reactive oxygen species (ROS), it must undergo stress-induced remodeling to maintain structural integrity and modulate immune recognition during infection^45–48^. The cell walls of *Candida* species are organized as a multilayered assembly in which an inner framework of chitin, β−1,3-glucan, and β−1,6-glucan supports an outer layer of mannoproteins extensively decorated with *N*- and *O*-linked mannans^49–52^. Given that phosphate acquisition and signaling via Pho84 are linked to oxidative stress responses, phosphate homeostasis is expected to influence both cell-wall organization and the fungal capacity to withstand host-derived oxidative attack^53^. Most studies probing oxidative stress in *C. albicans* have used high concentrations of H_2_O_2_, which due to their global inhibitory effect do not fully capture active molecular remodeling of cell-wall structures^54–57^. In addition, the phosphate deprivation that *C. albicans* experiences in the host simultaneously with oxidative stress, may alter its responses to stress.

Recent advances in solid-state NMR methodologies, which now allow in situ analysis of cell wall composition, hydration, and dynamics across a broad range of fungal species^58–60^. Recently, these approaches have been applied to *Aspergillus* (*A. fumigatus*, *A. nidulans*, and *A. sydowii*)^61–67^, other filamentous fungi including *Neurospora*, *Mucor*, *Rhizopus*, and *Schizophyllum*^68–71^, and yeast cells of *Cryptococcus*^72–75^, *Schizosaccharomyces*^76,77^, and *Candida* species^78^. In particular, recent in situ solid-state NMR work showed that although *C. albicans* and *C. auris* share the same hierarchical cell-wall architecture, their remodeling strategies diverge under echinocandin exposure: *C. albicans* thickens and reinforces its rigid core, whereas *C. auris* maintains wall integrity by upregulating β−1,6-glucan biosynthesis^78^. These findings provide a structural framework for interpreting stress-dependent remodeling in *Candida* cell walls, including the responses to ROS and phosphate deprivation. However, previous investigations of oxidative stress were performed under high phosphate conditions of laboratory culture media that do not reflect the host environment, where *C. albicans* simultaneously experience phosphate deprivation and oxidative stress.

In this work, we use a moderate, sublethal concentration of 0.8 mM H_2_O_2_ to examine oxidative stress responses under physiologically relevant conditions by comparing phosphate-replete and phosphate-deprived *C. albicans* cells. Using multidimensional ^13^C- and ^1^H-detected solid-state NMR, we directly observe structural changes in intact living cells, focusing on the organization and dynamics of cell wall polysaccharides. We compare the wildtype strain with a mutant lacking all four inorganic phosphate transporters (*pho84Δ/Δ pho89Δ/Δ pho87Δ/Δ fgr2Δ/Δ*) to assess how phosphate availability shapes oxidative stress responses and cell wall remodeling. Although Pho84 plays the dominant role in phosphate availability linked processes^25^, all four inorganic phosphate transporter deficient strain enables us to examine the structural consequences of severely restricted phosphate uptake. Our results demonstrate that phosphate availability reshapes cell wall architecture and modulates the oxidative stress response, revealing a mechanistic link between phosphate metabolism and cell wall resilience.

## Results

### Phosphate transport deficiency fully remodels the cell wall core in *C. albicans*

**Fig. 1:**
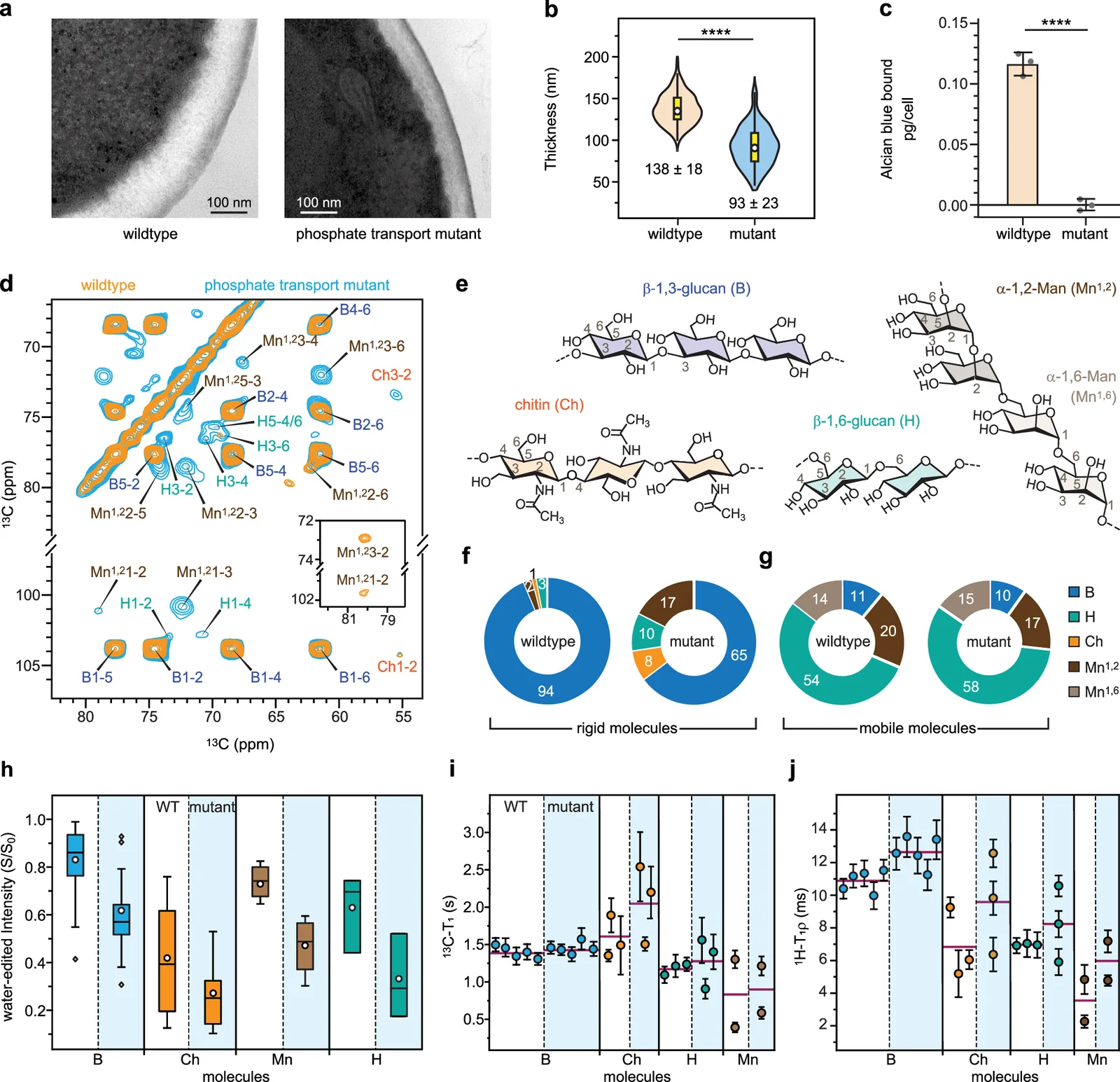
Phosphate-transport mutant of *C. albicans* displays altered cell wall structure. **a** TEM images of wildtype and phosphate transport mutant of *C. albicans* showing thinner cell walls for the mutant. **b** Cell wall thickness in wildtype (JKC 915; orange) and phosphate transport mutant (JKC 2830; blue) of *C. albicans*. Measurements represent 100 measurements of cell wall thickness collected from 10 individual cells (*n* = 10) for each sample. Black rectangles denote the interquartile range (IQR). White circles represent the mean of the dataset, and black vertical lines correspond to 1.5IQR. The mean ± s.d. is provided. Statistical analysis was performed using a two-tailed Welch’s *t*-test comparison between two samples. ****p-value  <  0.0001 (*p* = 4.63 × 10^-34^). **c** Decreased phosphomannan content of phosphate transporter mutant compared with wildtype cells. Cells were stained with alcian blue and the phosphomannan-bound alcian blue was plotted. Histograms: mean with s.d. of 3 biological replicates (*n* = 3). Statistical analysis was performed using a two-tailed Student’s *t*-test ****p-value  <  0.0001 (*p* = 4.25× 10^-5^). **d** Rigid-polysacchari**d**e carbon signals detected by 2D ^13^C-^13^C CORD spectra from wildtype (orange) and phosphate transport mutant (light blue) *C. albicans* cell walls. The inset shows weak mannose signals in the wildtype sample, which are only visible after lowering the contour levels of plotting. NMR abbreviations are used for resonance assignment. β−1,3-glucan: B; chitin: Ch; β−1,6-glucan: H; α−1,2-mannose units: Mn^1,2^; α−1,6-mannan units: Mn^1,6^. For example, B1-2 denotes the cross peak between carbons 1 and 2 in β−1,3-glucan. **e** Simplified structures of major polysaccharides. Molar compositions of **f** rigid polysaccharides and **g** mobile carbohydrates were estimated from resolved cross-peak volumes in 2D ^13^C CORD spectra and DP-refocused J-INADEQUATE spectra, respectively. Numbers represent percentages. **h** Box-and-whisker plot showing the water-edited intensities (S/S_0_) of β−1,3-glucan (blue), chitin (orange), α−1,2-mannose (brown), and β−1,6-glucan (green) in wildtype cells (WT; white background) and in the phosphate transport mutant (light-blue background). For each sample, data points represent individual resolved cross-peak intensity ratios extracted (*n* = 36 for β−1,3-gluca*n* (B), *n* = 5 for chitin (Ch), *n* = 4 for α−1,2-mannose (Mn), and *n* = 3 for β−1,6-gluca*n* (H)). The boxes represent the IQR, with whiskers extending to 1.5 IQR. For β−1,6-glucan, all data points are within the range between the first and third quartile, thus there are no whiskers. Mean values are represented by open circles and medians by purple horizontal lines. **i** ^13^C-T_1_ and **j** ^1^H-T_1ρ_ relaxation time constants of rigid cell wall polysaccharides in wildtype cells (white background) and in the phosphate transport mutant (light blue background). For both panels, error bars represent s.d. of the fitted parameters for each carbon site, and the horizontal lines in magenta indicate the mean of relaxation time constant across all resolved carbon sites for each polymer (*n* = 5 for β−1,3-gluca*n* (B), *n* = 3 for chitin (Ch), *n* = 3 for β−1,6-gluca*n* (H) and *n* = 2 for α−1,2-mannose (Mn)). Source data are provided as a source data file.

To define how phosphate acquisition influences cell wall architecture in *C. albicans*, we combined ultrastructural, biochemical, and solid-state NMR analyses of wildtype and phosphate transport mutant cells. During phosphate starvation, wildtype and mutant cells displayed no substantial differences in overall morphology (Supplementary Fig. 1). However, transmission electron microscopy (TEM) revealed a marked reduction in cell wall thickness from 138 ± 18 nm in wildtype cells to 93 ± 11 nm in the mutant, indicating that phosphate uptake is required to maintain normal cell wall structure and integrity (Fig. 1a, b). Consistent with this structural change, independent quantification of cell wall phosphomannan by Alcian blue staining showed a dramatic reduction in phosphomannan content in the phosphate transport mutant (Fig. 1c), revealing extensive remodeling of the mannan layer.

To characterize these molecular-level changes while preserving native cell wall organization, the ^13^C-labeled cells of *C. albicans* were kept intact and viable during solid-state NMR analysis. Prior to isotopic labeling, the cultures were subjected to sequential phosphate-deprivation cycles to establish a defined phosphate-starved physiological state, ensuring that the observed cell wall features arise from altered phosphate acquisition and signaling rather than from remaining intracellular or extracellular phosphate reserves. Without chemical perturbations, all molecules preserved their native properties and supramolecular organization. Using a variety of polarization techniques, we can achieve selective detection of molecules across distinct dynamical regimes (Supplementary Fig. 2). The rigid fraction was selectively detected through ^1^H-^13^C cross-polarization (CP), which favors rigid molecules exhibiting large C-H dipolar couplings, and was projected into a 2D ^13^C-^13^C correlation spectrum for improved spectral resolution. In wild-type cells, this method predominantly detected β-1,3-glucan signals (Fig. 1d). Representative cross peaks include correlations of carbon 1 with carbons 5, 2, and 4 (B1-5, B1-2, and B1-4), and correlations involving carbon 6 (B4-6, B5-6, and B1-6), enabling assignment of ^13^C chemical shifts for individual carbon sites within the β-1,3-glucan structure (Fig. 1e).

Surprisingly, the rigid fraction of the phosphate transport mutant exhibited substantially increased spectral complexity. In addition to strong β-1,3-glucan signals, the chitin (e.g., Ch3-2 and Ch1-2) and β-1,6-glucan (*e.g*., H3-6) signals were significantly more intense than in the wild type. Moreover, signals corresponding to α-1,2-linked mannose units, which form diverse side chains decorating the α-1,6-linked backbone of mannan in *C. albicans* (Fig. 1e), became prominent in the mutant. Notable examples include cross peaks between carbons 1 and 3 (Mn^1,2^1-3) and between carbons 2 and 3 (Mn^1,2^2-3) (Fig. 1d). Peak-intensity analysis showed that mannan, chitin, and β-1,6-glucan constitute 17%, 8%, and 10% of the rigid polymers in the mutant, respectively, whereas the wildtype cell wall is dominated by β-1,3-glucan (94%) and contains only small amounts of chitin (1%), β-1,6-glucan (3%), and mannan sidechains (2%) in the rigid fraction (Fig. 1f).

In contrast, no substantial changes were observed in the mobile fraction. This fraction was selectively detected using a combination of ^13^C direct polarization (DP) and a short recycle delay, which preferentially excites molecules with rapid ^13^C-T_1_ relaxation and suppresses signals from rigid components that relax slowly. This polarization scheme, combined with 2D refocused J-INADEQUATE spectroscopy, enabled unambiguous identification of the carbon signals and connectivities in mobile cell-wall polymers (Supplementary Fig. 3). We identified both the α-1,6-linked mannan backbone and α-1,2-linked mannose sidechains, as well as β-1,6-linked and β-1,3-linked glucans and their β-1,3,6-linked branching sites. Intensity analysis revealed similar mobile-domain compositions for both wild-type and mutant cells: 54-58% β-1,6-glucan, 32-34% mannan, and 10-11% β-1,3-glucan (Fig. 1g).

Because the rigid fraction plays a central role in cell-wall remodeling in the phosphate transport mutant, we next examined how the phosphate transport defect perturbs hydration and dynamics of rigid biopolymers. Hydration profiles were assessed using water-to-carbohydrate ^1^H polarization transfer experiments, where the S/S_0_ ratio (water-accessed versus control) reflects water accessibility at specific carbon sites (Supplementary Fig. 4). These ratios were considerably lower in the mutant than in the wildtype, decreasing from 0.83 to 0.64 for β-1,3-glucan and from 0.41 to 0.27 for chitin (Fig. 1h). The few weakly resolved peaks attributed to β-1,6-glucan and mannan sidechains exhibit higher S/S_0_ ratios than chitin but lower than those of β-1,3-glucan. Moreover, both β-1,6-glucan and mannan show a decrease in the S/S_0_ ratio. These observations are reasonable because β-1,3-glucan has recently been identified as a key determinant of cell-wall hydration across diverse fungal species. Therefore, the one-third reduction of β-1,3-glucan in the mutant cell wall inevitably compromises the ability of carbohydrate polymers in the rigid core of the *C. albicans* cell wall to retain water molecules.

The motions of polysaccharides were probed on two distinct timescales: ^13^C-T_1_ relaxation to assess local nanosecond dynamics and ^1^H-T_1ρ_ relaxation to probe larger-scale collective motions on the microsecond timescale (Supplementary Fig. 5). Chitin showed marked increases in the averaged values of both ^13^C-T_1_ from 1.6 s in the wildtype to 2.1 s in the mutant (Fig. 1i) and ^1^H-T_1ρ_ from 6.8 to 9.6 ms (Fig. 1j), signifying reduced mobility on both timescales. In contrast, β-1,3-glucan only exhibited an increase in ^1^H-T_1ρ_ from 10.8 ms to 12.6 ms, indicating enhanced microsecond-scale dynamics without corresponding nanosecond-scale restriction.

The small amount of mannan side chains detectable in the rigid phase remains relatively mobile, as evidenced by very short ^13^C-T_1_ and ^1^H-T_1ρ_ relaxation time constants. This indicates that partial mobility persists even after these sidechains come into close proximity with and become partially rigidified by chitin and β-1,3-glucan. In contrast, β-1,6-glucan exhibits ^13^C-T_1_ values comparable to those of β-1,3-glucan but shows greater nanosecond-scale dynamics, as reflected by a shorter ^1^H-T_1ρ_. Both molecules also showed an increase in relaxation time constants from the wild type to the mutant.

In summary, the phosphate transport defect profoundly remodeled the rigid core of the *C. albicans* cell wall. This remodeling involves reshaping both the molecular composition and the physical properties of the rigid scaffold. Whereas the wild-type rigid cell wall is dominated by β-1,3-glucan, the mutant incorporates increased levels of chitin and β-1,6-glucan, together with some mannan side chains that interact with these components. The cell wall also exhibits decreased water accessibility and reduced molecular mobility, resulting in a stiffer, less permeable barrier. This remodeling strategy in response to phosphate deprivation resembles cell-wall adaptations triggered by external stressors, such as antifungal treatment with echinocandins or exposure to a hypersaline environment^61,64,70,78^.

### ^1^H-detected solid-state NMR reveals reduced polymorphism in mobile polysaccharides

It is fully unexpected that molecules in the mobile fraction did not exhibit substantial changes in composition. One possibility is that more subtle structural variations, such as conformational changes, occurred but escaped detection in ^13^C-based experiments due to limited spectral resolution. Therefore, we applied ^1^H-detected solid-state NMR spectroscopy to take advantage of its enhanced spectral sensitivity and the strong dependence of ^1^H chemical shifts on subtle carbohydrate structural differences. This approach enables improved resolution of the polymorphic forms of mobile polysaccharides, which are often poorly distinguished in ^13^C-detected spectra due to extensive chemical-shift degeneracy.

**Fig. 2:**
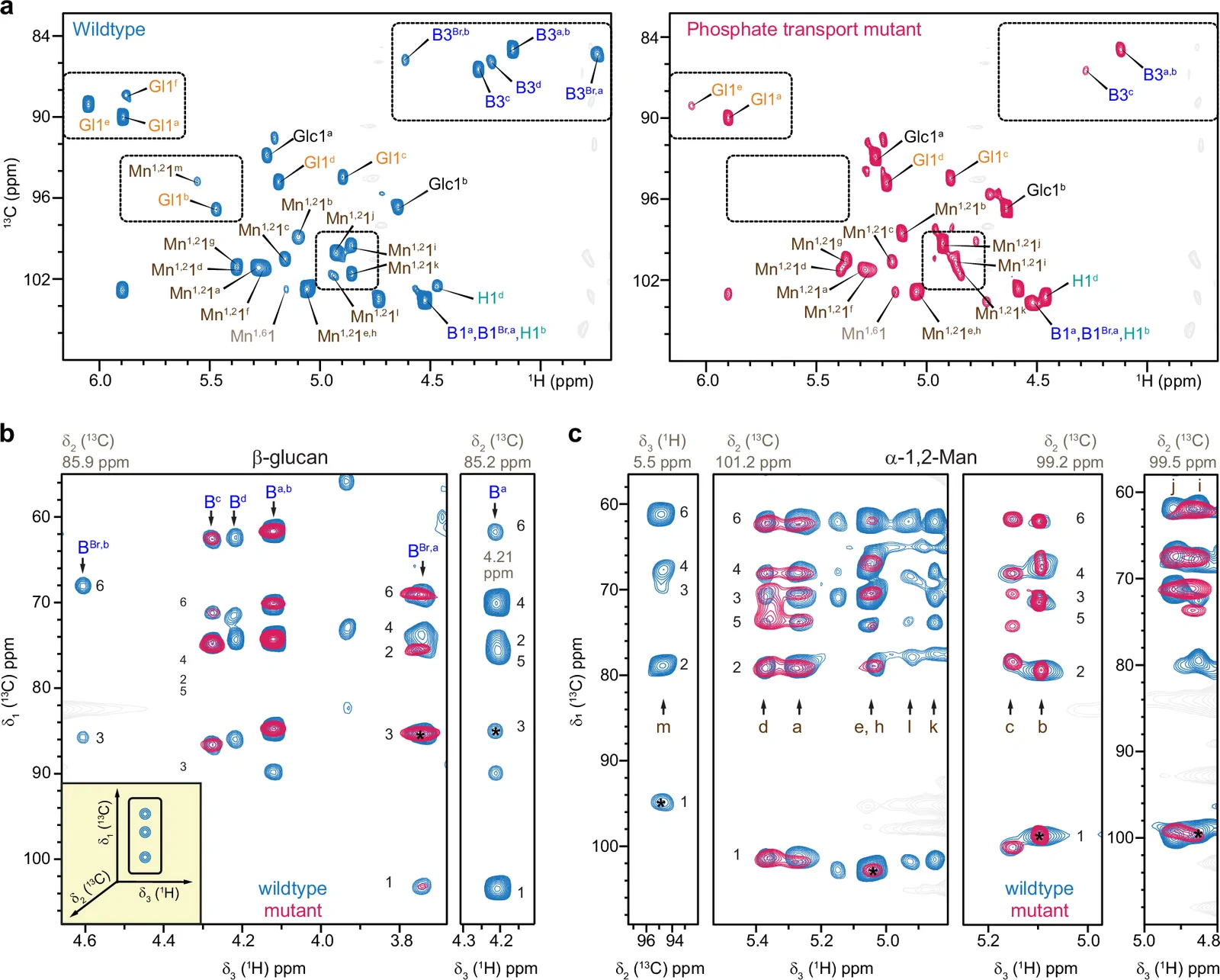
Proton-detected solid-state NMR shows reduced structural polymorphism in mutant walls. **a** Comparison of 2D hcCH TOCSY (DIPSI-3) spectra of wildtype (blue, left) and phosphate transport mutant (magenta, right) strains of *C. albicans*, showing one-bond ^13^C-^1^H correlations. Water signals are in gray. Dotted boxes highlight spectral regions where the mutant displayed fewer carbohydrate peaks relative to wildtype cells. Mn: mannan; B: β-1,3-glucan; B^Br^: branching site of the 1,3,6-Glc unit; H: β-1,6-glucan; Mn^1,2^: α-1,2-Man; Mn^1,6^: α-1,6-Man; Glc: glucose; Gl: galactose or glucose derivatives. Numbers indicate the carbon/proton position, and superscript letters denote different structural forms of a molecule (*e.g*., B3ᵈ denotes the C3-H3 cross peak belonging to type-d β-1,3-glucan). **b** β-glucan and **c** α-1,2-Man exhibit multiple structural allomorphs as resolved from ^13^C-^1^H (δ_1_-δ_3_) and ^13^C-^13^C (δ_1_-δ_2_) strips extracted from 3D hCCH TOCSY (DIPSI-3) spectra. Inset illustrates the extraction of a 2D ^13^C-^1^H (δ_1_-δ_3_) plane from a 3D spectrum. The spectra show through-bond carbon-carbon connectivity (carbon numbers labeled) for each molecule or structural form along the vertical signal traces. Numbers 1-6 indicate specific carbon sites within each molecule. 2D planes were extracted at distinct carbon (δ_2_) and proton (δ_3_) sites, indicated at the top of each panel in gray. The strip extracted at a specific carbon or proton site is indicated by an asterisk. In wild-type cells (blue), resolved signals are assigned to three forms of β−1,3-glucan (B^a^, B^b^, and B^c^) and two forms of branching sites in β-1,3-glucan (B^Br,a^ and B^Br,b^) in Panel B, and eleven forms of α-1,2-Man units with well-resolved carbon connectivity in (**c**). The phosphate transport mutant (magenta) displayed substantially reduced structural polymorphism, with only two types of β-1,3-glucan, one form of the branching site, and seven of the α-1,2-Man forms detected.

The 2D J-hCCH TOCSY spectra of intact *C. albicans* cells revealed marked differences in the carbohydrate anomeric region between wild-type and mutant strains (Fig. 2a). In particular, the α-1,2-linked mannose units showed a substantial reduction in peak multiplicity in the phosphate transport mutant. For example, the Mn^1,2^1^m^ and Mn^1,2^1^j^ peaks corresponding to the C1/H1 sites of the type-m and type-j forms of α-1,2-linked mannose residues completely disappeared. In addition, signals corresponding to the type-j, type-k, and type-i forms merged into a single continuous band in the mutant, rather than three discrete peaks as observed in the wild-type cells. Spectral simplification was also observed for the Gl1 signals corresponding to C1/H1 of Glc/Gal and their derivatives, where type-f and type-b were absent in the mutant. β-1,3-glucan also exhibited reduced structural complexity in the mutant, as demonstrated by the decreased number of peaks for its C3/H3 sites. Signals corresponding to type-d units and branching sites were weaker or absent in the mutant. These results indicate that phosphate transport deficiency leads to a more homogeneous molecular structure within the mobile matrix of the cell wall, despite the overall molecular composition remaining unchanged. This suggests that either the modification of mobile polysaccharides is impeded, reducing their chemical diversity, or that the polymers adopt more homogeneous conformations due to simplified intermolecular interactions and enrichment of energetically favored conformations.

Extending the 2D experiment into its 3D format, the J-hCCH TOCSY experiment incorporating DIPSI-3 mixing enabled tracking of carbon connectivities through J-coupling and allowed unambiguous site-specific assignments for each allomorph^65,75,79^. For β-1,3-glucans, type-a and type-b displayed overlapping signals (B^a,b^) in both samples in the stripe extracted at ^1^H chemical shift of 3.75 ppm. However, the characteristic type-a peaks (B^a^) extracted at ^1^H chemical shift of 4.21 ppm were only detected in the wild type, indicating that type-b is present in both strains, whereas type-a is confined to the wild type. Type-c (B^c^) was detectable in both strains but was weaker in the mutant, and type-d (B^d^) was completely absent in the mutant, consistent with the 2D spectra. The first type of branching site B^Br,a^ was observed in both strains, although weaker in the mutant, while the second type B^Br,b^ was not detected in the mutant (Fig. 2b). These findings revealed a simplified β-1,3-glucan matrix, with reduced complexity in both the linear chains and the branching patterns, forming a more uniform structure across the *C. albicans* cell wall.

As mannan fibrils form the outer layer of the *C. albicans* cell wall, their side chains, predominantly comprising α-1,2-linked mannose units, play a critical role in stabilizing this layer through extensive intermolecular interactions. This leads to remarkable structural diversity of these side chains, reflected in the coexistence of 14 resolvable allomorphs in the spectra. Representative stripes from the 3D J-hCCH TOCSY spectra are shown in Fig. 2c, where the mutant has completely lost the signals corresponding to forms m, l, and k, and the j and i forms are substantially restructured, consistent with the 2D results but now with improved resolution and complete resonance assignments.

Together, these findings demonstrate that the phosphate transport defect significantly reduces the structural complexity accommodated by the mobile domain of the *C. albicans* cell wall, even without major perturbations to molecular composition. They also highlight the power of ^1^H-detected solid-state NMR for resolving polysaccharide polymorphism and subtle structural modifications at high sensitivity and resolution in intact cells.

### Oxidative stress induces distinct changes to polysaccharides in *C. albicans* cell walls

To assess the impact of oxidative stress on *C. albicans* cell wall architecture, both wildtype and phosphate transport deficient mutant cells were treated with 0.8 mM H_2_O_2_, a concentration selected to permit sustained growth of treated cells, as determined in pilot experiments that showed minor growth defects of cultures exposed to this concentration (Supplementary Fig. 6). Sublethal, sub-millimolar H_2_O_2_ elicits adaptive responses in *C. albicans* and may reflect the ROS levels generated during neutrophil and macrophage respiratory bursts in the host, enabling biologically meaningful oxidative stress without catastrophic loss of viability^27,36,55,80–82^. The aim of this experiment was to isolate oxidative stress-specific remodeling of the cell wall during growth of phosphate deprived cells. For this reason, the phosphate transport defective mutant and wildtype cells were cultured in standard, phosphate replete media for 8 h. In this medium, mutant cells are able to import some inorganic phosphate via their glycerophosphocholine transporters^25^.

TEM analysis revealed oxidative stress-associated changes in cell wall thickness in both strains. Wildtype cells increased from 118 ± 21 nm under control conditions to 127 ± 16 nm following H_2_O_2_ exposure, whereas the phosphate transport mutant increased from 104 ± 23 nm to 122 ± 27 nm. Both wild-type and phosphate transport mutant showed a comparable increase in cell wall thickness upon H_2_O_2_ exposure (Supplementary Fig. 7). Comparative analyses revealed marked differences in polysaccharide behavior that depend on polymer dynamics (Supplementary Figs. 8 and 9) and, likely, cell-wall organization. Surprisingly, we also observed perturbations that can only have arisen from the reaction of peroxide directly with cell wall components. This was despite the fact that due to its expected spontaneous disproportionation in the watery medium, no H_2_O_2_ was detectable in the medium at the time of the cell harvest. We ruled out cell morphologic changes as the causes of the observed cell wall alterations at 5 mM H_2_O_2_, hyperpolarized bud growth has been observed but no substantial morphological changes were observed in either wild-type or phosphate transport mutant under our conditions (Supplementary Fig. 6)^34^. Hence, we attribute the observed cell wall alterations to two factors, firstly, active remodeling of the wall by cells responding to oxidative stress, and secondly, the chemical reaction of peroxide with cell wall components.

**Fig. 3:**
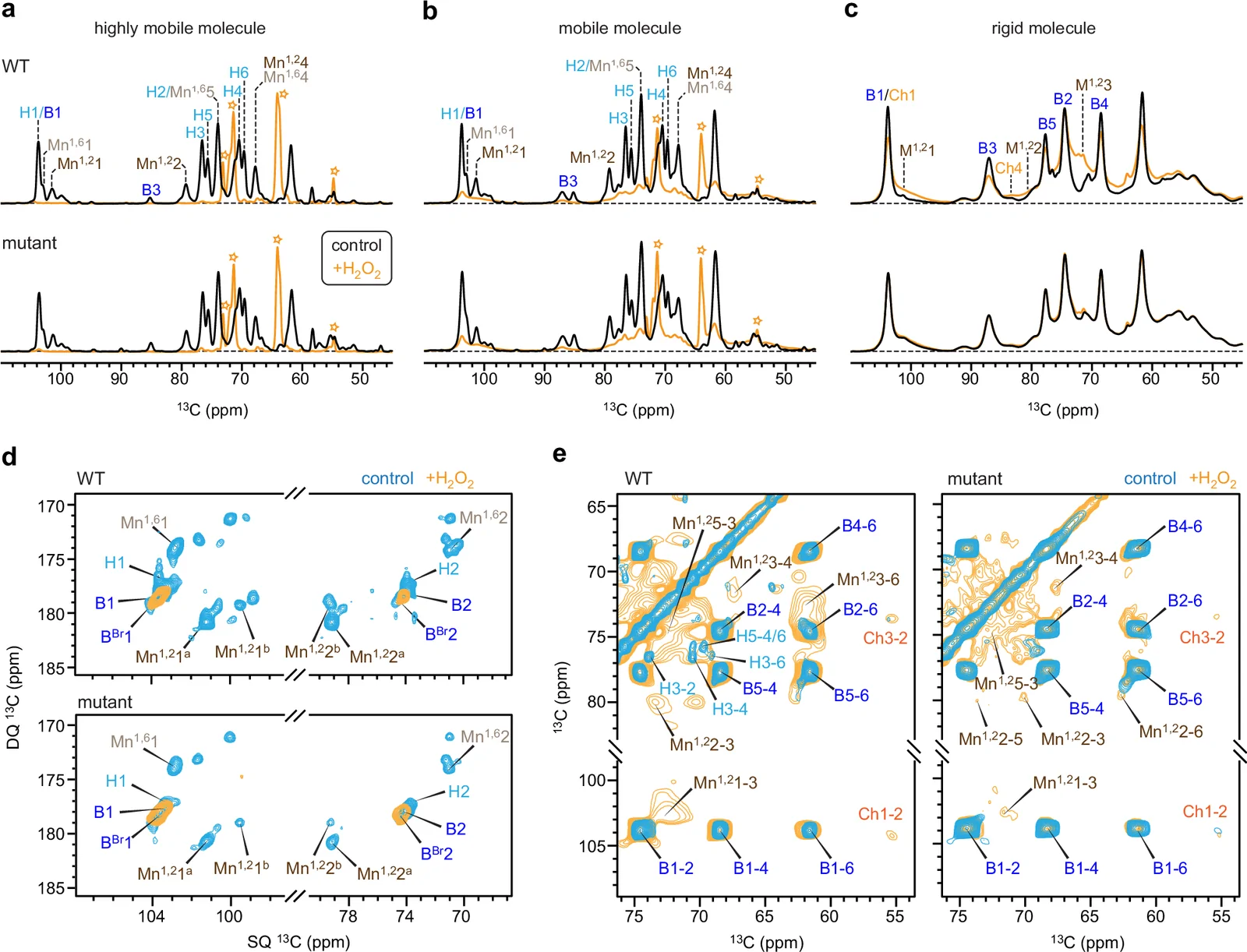
Alterations of polysaccharides in *C. albicans* cell walls during oxidative stress. 1D ^13^C NMR spectra were obtained using **a** INEPT to detect highly mobile polysaccharides, **b** DP to detect mobile polysaccharides, and **c** CP to detect rigid polysaccharides. Spectra are shown for wildtype (top) and mutant (bottom) cells under control (black) or H_2_O_2_-treated (orange) conditions. Reaction of H_2_O_2_ with carbohydrates generates paramagnetic species (free radicals), which broaden out the signals of associated carbohydrates. Dashed horizontal lines denote the spectral baseline. Open stars mark predominant lipid signals in the INEPT and DP spectra of H_2_O_2_-treated samples, where carbohydrate signals are broadened out due to their interactions with H_2_O_2_. **d** mobile molecules detected through 2D ^13^C DP refocused J-INADEQUATE spectra of wild-type (top) and mutant (bottom) cells under control (blue) or H_2_O_2_-treated (orange) conditions. H_2_O_2_ treatment nearly eliminates all mobile polysaccharide signals, except for β-1,3-glucans. (**e**) 2D ^13^C-^13^C CORD spectra of wild-type (top) and mutant (bottom) cells under control (blue) or H_2_O_2_-treated (orange) conditions. Mannan signals appear after H_2_O_2_ treatment, indicating a transition from the mobile fraction to the rigid cell-wall core.

Highly mobile and largely solvated carbohydrates, detected using the 1D refocused INEPT experiment (Fig. 3a), and the overall mobile fraction, detected using 1D ^13^C DP spectra (Fig. 3b), showed the characteristic signals of α-1,2-Man and α-1,6-Man in mannan sidechains and backbones, respectively, β-1,6-glucan, and partially β-1,3-glucan in the wild-type cells. This agrees with their localization: mannan is surface-exposed, and β-glucans are dynamically flexible and highly hydrated. After H_2_O_2_ exposure, these signals completely disappeared in both wildtype and mutant cells in the highly mobile fraction detected by INEPT (Fig. 3a). This is a signature feature of paramagnetic relaxation enhancement (PRE)^83,84^, in which NMR peaks are severely broadened by rapid transverse relaxation arising from the presence of paramagnetic species, such as radicals formed when carbohydrates react with H_2_O_2_, which generates hydroxyl and related radicals capable of abstracting hydrogen from polysaccharide residues, forming carbon or oxygen centered radicals^85–89^. Peroxide-induced structural changes to polysaccharides, such as hydrogen abstraction, degradation, or cross-linkin,g may further contribute to the signal loss, but to a lesser extent compared to paramagnetic relaxation enhancement. The remaining signals were dominated by lipids, which are located deeper in the fungal cell membrane below the cell-wall layers.

Carbohydrates in the overall mobile fraction detected by ^13^C DP combined with short recycle delays also exhibited a substantial reduction in signal intensity (Fig. 3b), but unlike the highly mobile fraction, these signals were not entirely lost. A residual pattern of broad peaks remained after treatment, indicating the presence of semi-mobile molecules with restricted accessibility to peroxides. These observations were consistent across both wild-type and mutant cells.

In contrast, rigid molecules detected by ^13^C CP showed only moderate line broadening in the wild type (Fig. 3c). A more pronounced change occurred in mannan, where certain α-1,2-Man units displayed stronger signals in the rigid fraction. The phosphate transport mutant, however, exhibited little to no radical-induced paramagnetic broadening among mannan and glucan peaks and showed reduced compositional changes in the rigid fraction, suggesting a decreased remodeling response to oxidative stress. Although the phosphate transport mutant exhibits reduced polymorphism and greater structural homogeneity in the mobile polysaccharide matrix, as shown by hCCH TOCSY (DIPSI-3) spectra in Fig. 2, the broader ^13^C CP signals reflect increased conformational heterogeneity within the distinct rigid cell-wall core probed in Fig. 3c.

Higher-resolution analysis of mobile molecules using 2D ^13^C DP-INADEQUATE spectra revealed that signals corresponding to β-1,6-glucan and mannan were completely absent after oxidative stress, whereas β-1,3-glucan signals partially persisted (Fig. 3d and Supplementary Fig. 10). The rigid fraction showed more complex changes: the 2D ^13^C-^13^C CORD spectra revealed strong paramagnetic broadening in the wildtype, particularly for α-1,2-mannose units and β-1,6-glucan (Fig. 3e). The severe broadening of α-1,2-mannose resonances in the wildtype indicates direct interaction of peroxide and the radicals generated upon reaction with this polysaccharide. In contrast, the mannan signals remained substantially sharper in the phosphate transport mutant, confirming that mannans in the rigid core of the mutant cell wall did not react with peroxide to generate radicals. These peroxide-induced line-broadening effects were further confirmed by analysis of 1D cross-sections extracted from the 2D CORD spectra (Supplementary Fig. 11), enabling direct assessment of resonance linewidths and PRE-associated signal attenuation otherwise masked by spectral congestion in rigid polysaccharide resonances. These oxidative stress-induced spectral changes were consistently observed in an independent biological replicate prepared and analyzed using the same sample preparation protocol. The replicate reproduced the major spectral features of the primary dataset, including H_2_O_2_-induced broadening of polysaccharide resonances in the rigid cell wall fraction and the differential response between the wild-type and phosphate transport mutant cell walls (Supplementary Figs. 12 and 13). Consistent with these observations, full width at half maximum (FWHM) values of the resolved α-1,2-mannose and β-1,3-glucan resonances from the 2D CORD spectra showed similar linewidth trends across the two biological batches (Supplementary Table 6).

These findings provide deeper insights into how the cell wall is remodeled physiologically versus during phosphate scarcity, in response to ROS. In the wild type, the inner core undergoes significant reorganization, in which a portion of the originally mobile α-1,2-mannose units and β-1,6-glucan is incorporated into the rigid core where it interacts with chitin and β-1,3-glucan to reinforce the structural scaffold. In the phosphate transport mutant, peroxide induces much weaker reinforcement of the rigid core. Although α-1,2-mannose units and β-1,6-glucan also redistribute partially into the rigid core after H_2_O_2_ exposure, the extent is substantially reduced. Peroxide preferentially targets the mobile, surface-exposed polysaccharides, primarily mannan, and the underlying β-1,6-glucans in the intermediate layer of the *C. albicans* cell wall. Thus, the flexible outer polysaccharide layer is highly vulnerable to oxidative damage. These results demonstrate that cell wall remodeling during the physiologic oxidative stress response requires phosphate repletion of the cell. Defective phosphate transport impairs the cell wall remodeling processes that optimize *C. albicans’* ability to withstand oxidative stress.

Interestingly, when *C. albicans* cells previously exposed to hydrogen peroxide were profusely washed, the post-wash spectra showed sharp, well-resolved resonances, particularly for dynamic polysaccharides such as α-1,2-mannose and β-1,6-glucan (Supplementary Figs. 14–16). This recovery indicates efficient quenching of radicals generated during peroxide exposure and confirms that the peroxide-induced loss of NMR signals was primarily due to paramagnetic relaxation enhancement. These carbohydrate radicals appear to be transient and can be removed by simple washing, restoring hydroxyl groups or causing only minimal modification, while irreversible processes such as covalent cross-linking or bond cleavage have not occurred or have occurred only in a very small fraction of molecules. These findings demonstrate that oxidative damage to the mobile domain of the cell wall, mediated through radicalization of flexible mannans and branched glucans, is largely reversible.

### Molecular Dynamics simulation reveals non-specific H_2_O_2_ reactivity with polysaccharides

**Fig. 4:**
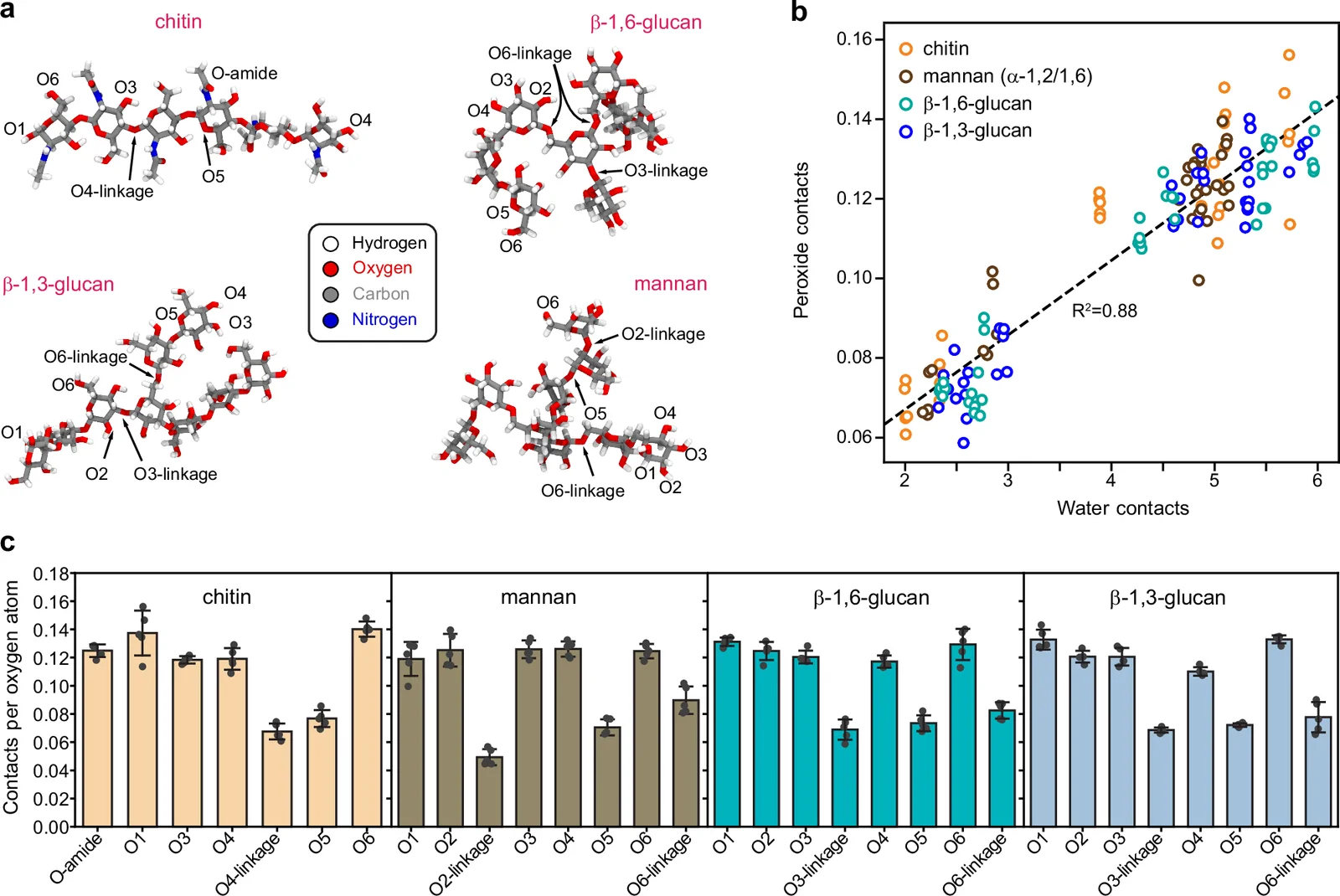
MD simulations reveal peroxide accessibility independent of polysaccharide type. **a** Representative MD snapshots illustrating the atom-naming conventions used in this study, highlighting the specific oxygen atoms that can interact with H_2_O_2_. **b** Scatter plot comparing the contacts that each labeled oxygen atom forms with either water or hydrogen peroxide during the simulations. Each point represents a single simulation and specific oxygen position; data points are colored by polysaccharide chain type. Dashed line represents the linear regression, which demonstrates a strong fit with an *R*^2^ value of 0.88. **c** Quantification of H_2_O_2_ contacts per oxygen atom per simulation snapshot across the four glucan chain types analyzed. Bar height represents the mean, and error bars represent the standard deviation, computed based on the variance observed across the five simulation repeats (*n* = 5) for each glucan chain type.

When examining the direct chemical effects of peroxide on cell wall components, the next question was whether the distinct oxidative responses of cell-wall polysaccharides arise from intrinsic chemical reactivity or from different degrees of accessibility within the differently organized cell walls of wild-type and mutant strains. To evaluate whether particular polysaccharides or structural motifs are preferentially exposed to oxidative attack, we performed molecular dynamics (MD) simulations to assess the interactions between hydrogen peroxide and representative fungal polysaccharides in water (Supplementary Fig. 17). These included chitin, mannan, β-1,6-glucan, and β-1,3-glucan incorporating branched structures (Fig. 4a).

Across all examined oxygen sites, including those within glycosidic linkages, the frequency of peroxide contacts varied only modestly in the range of 0.06-0.16 (Fig. 4b). Even oxygen atoms expected to be sterically shielded, such as those participating in backbone linkages, showed only slightly reduced interactions with peroxide. Consistent with this, a strong linear correlation was observed between the number of water contacts and peroxide contacts. Oxygen sites that were more frequently hydrated were also more frequently contacted by peroxide, indicating that interaction is governed primarily by solvent accessibility rather than inherent chemical preference. Thus, to first approximation, all oxygen atoms within these polysaccharides are susceptible to oxidative attack.

A detailed comparison of individual oxygen positions revealed a consistent trend across all polysaccharides (Fig. 4c). Oxygen atoms forming the glycosidic linkages (e.g., O3 and O6 in β-glucans, O2 and O6 in mannan, and the O4-linkage in chitin) exhibited fewer peroxide contacts than the hydroxyl oxygens. O5, which is involved in ring closure and is less solvent-exposed, showed a similar decrease. Aside from these predictable reductions, the overall range of peroxide contacts was highly similar among chitin, mannan, β-1,6-glucan, and β-1,3-glucan. This consistency demonstrates that the detailed chemistry of the carbohydrate backbone does not strongly bias peroxide association. Instead, solvent exposure of hydroxyl groups is the dominant determinant of peroxide interaction, regardless of polysaccharide type. Although the absolute differences in contact frequency are small, the extensive sampling in the simulations ensures that the observed trends are statistically robust. Consequently, the most probable sites of glucan peroxidation correspond to the most solvent-accessible oxygen atoms, reinforcing the notion that direct oxidative damage preferentially targets the exposed and dynamic domains of the fungal cell wall. Future simulations incorporating packed polysaccharide assemblies that mimic native wild-type and phosphate-deficient cell-wall architectures may further clarify how mesoscale organization influences water and peroxide accessibility.

## Discussion

*Candida albicans* is the most commonly isolated invasive fungal pathogen of humans. This is because, unlike environmental fungal pathogens like the *Aspergilli* and *Mucorales*, it is already present on most humans’ mucous membranes and hence is poised to invade their tissues and bloodstream when epithelial barriers and innate immune defenses weaken. However, the transition from life as a commensal to life as an invasive pathogen exposes *C. albicans* cells to dramatic changes in environmental conditions. In addition to increased ambient oxygen tension and pH, *C. albicans* cells experience sharp nutritional shifts, including phosphate scarcity.

Simultaneously with phosphate deprivation during the transition from commensal to invasive pathogen, *C. albicans* in most human hosts experiences the attack of innate immune cells that unleash superoxide anions and antimicrobial peptides onto the fungal cell. Superoxide dismutases at the fungal surface convert this product of host cells’ NADPH oxidase into H_2_O_2_, which diffuses readily across membranes and induces intracellular oxidative stress^90–92^. Given the importance of ROS in the host-pathogen interaction, numerous studies have focused on the impact of oxidative stress on *C. albicans* cells. They have, however, examined oxidative stress responses during the phosphate repletion state that *C. albicans* experiences as a commensal, while the constraints to its oxidative stress responses during the phosphate scarcity typical for its invasive infectious lifestyle have not been investigated.

As a large organelle that interfaces directly with the host, the cell wall is particularly important for *C. albicans*’ ability to withstand stress. Its three wall-anchored superoxide dismutases are a first line of defense against host-generated superoxide^90^. The H_2_O_2_ produced by their catalytic activity may diffuse away, may enter the *C. albicans* cell and cause internal oxidative stress, and may, as we now find, directly alter the chemistry of the structural cell wall polysaccharides.

Intracellular ROS like H_2_O_2_ and the product of its reaction with iron-sulfur clusters, the hydroxyl anion, inflict oxidative damage on membrane lipids, nucleic acids, and proteins. Signaling pathways mediated by the stress-activated kinase Hog1 and the transcription factor Cap1 coordinate induction of protective genes: for example, the *hog1*Δ mutant is hypersensitive to ROS^39^. Additional regulators such as the zinc-finger factor Sfp1 and the DNA-binding protein Rap1 have been implicated in fine-tuning ROS response and antioxidant gene expression, linking oxidative stress defense to global transcriptional and metabolic regulation^93^. In this way, protective intracellular enzymes are induced, like superoxide dismutases, catalase, glutathione- and thioredoxin-dependent systems, which detoxify ROS and repair oxidative damage^94^. Overall, ROS remain a potent challenge to *C. albicans*: combined stresses (*e.g*., oxidative plus cationic stress) can suppress antioxidant responses (via inhibition of Cap1 nuclear accumulation) and kill fungal cells, contributing fundamentally to host innate immune efficacy^95^.

While in vivo, phosphate deprivation and oxidative attack on *C. albicans* cells occur together during their transition period from commensal to pathogenic lifestyle, we here sought to isolate the effects of these stressors on the cell wall as well as characterize their combined effect on the cell wall. Although experiments performed in defined synthetic media cannot fully recapitulate the complexity of host-associated stresses, this controlled reductionist approach enables mechanistic isolation of phosphate-dependent oxidative-stress responses. We first defined in molecular detail the changes in cell wall architecture induced by phosphate deprivation, as the cell seeks to preserve precious phosphate for essential functions like nucleic acid biosynthesis and core carbon metabolism. We then exposed cells unable to import sufficient phosphate, the phosphate transport mutants, versus phosphate-replete wild-type cells to a sublethal H_2_O_2_ concentration, examining the effect of combined phosphate deprivation and peroxide stress.

Our findings extend current models by showing that ROS susceptibility is strongly shaped by cell-wall accessibility and architecture, not only by intracellular antioxidant capacity. We identify phosphate availability as a major determinant of wall structure and oxidative resilience, showing that phosphate homeostasis shapes not only intracellular metabolism but also the organization and dynamics of the extracellular wall. By demonstrating that the mobility, exposure, and redistribution of polysaccharides govern ROS interactions, we establish the cell wall as an active participant in oxidative protection and show that nutrient availability acts as a structural regulator of ROS resistance.

By analyzing intact *C. albicans* cells, we show that severe restriction of inorganic phosphate uptake extensively remodels the rigid wall core: the β-1,3-glucan-dominated scaffold characteristic of wild-type cells is replaced by a composite enriched in chitin, β-1,6-glucan, and mannan side chains, and exhibits reduced hydration and molecular mobility. This remodeling resembles stress-induced reinforcement observed under echinocandin or osmotic challenge, revealing that nutrient limitation alone can reconfigure the rigid scaffold and establishing phosphate availability as a structural regulator of the fungal cell wall. Phosphate-dependent changes in wall architecture also govern susceptibility to ROS. Hydrogen peroxide primarily targets the highly mobile, surface-exposed mannans and β-1,6-glucans, whereas the rigid core remains comparatively protected. In wildtype cells, oxidative exposure drives some mobile polymers into the rigid scaffold, reinforcing the wall; this response is markedly diminished in phosphate transport mutants. Under the moderate concentration used in this study, peroxide-induced radicalization of mobile polysaccharides is found to be reversible, consistent with a transient redox-buffering mechanism, and H_2_O_2_ lacks intrinsic polymer specificity, reacting instead with solvent-exposed and dynamic sites. Together, these findings establish phosphate transport and cell-wall architecture as key determinants of oxidative susceptibility and identify accessibility, rather than monomer identity, as the principal regulator of ROS interaction in fungal cell walls.

**Fig. 5:**
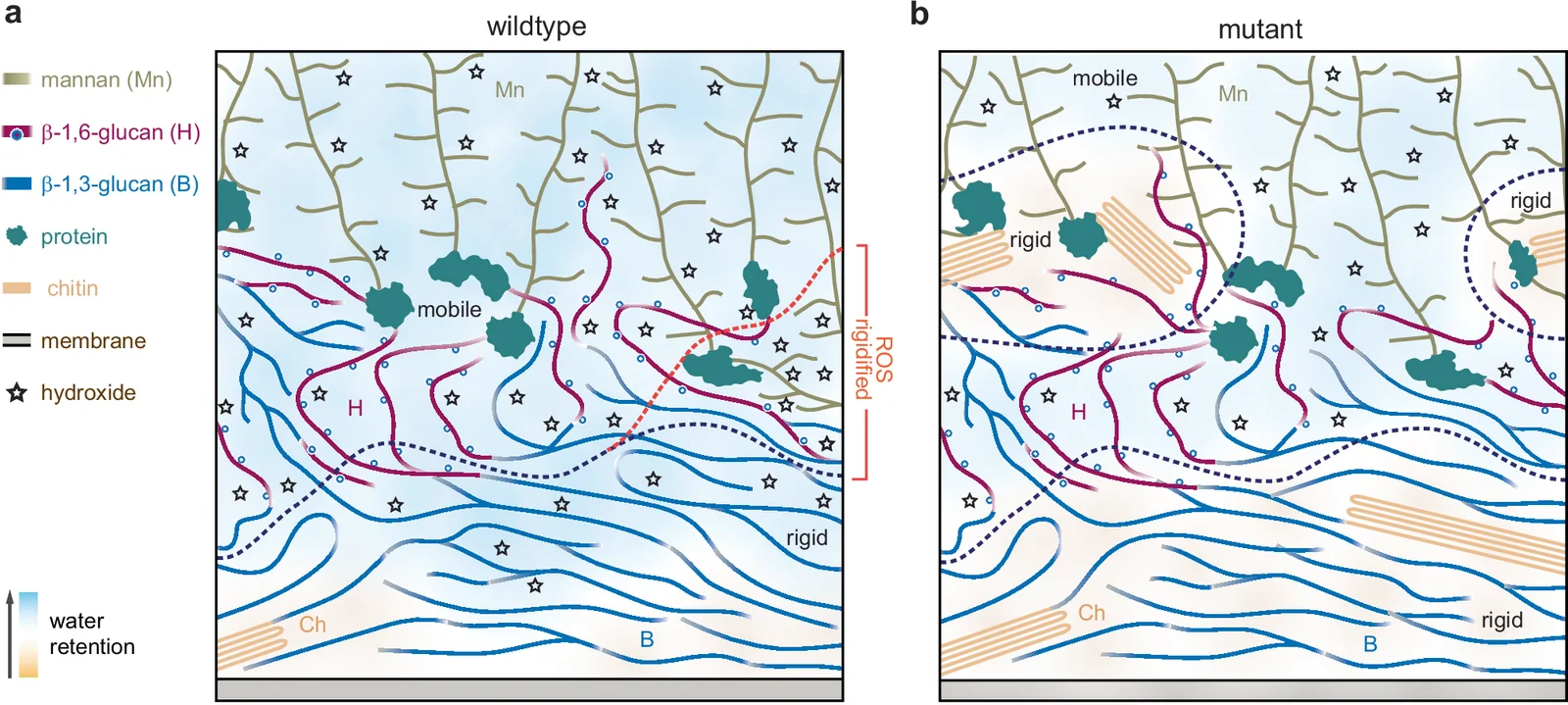
Schematics of wildtype and mutant *C. albicans* cell walls and peroxide retention. **a** Organization of the wildtype cell wall showing distinct inner and outer domains (separated by black dashed lines). Blue ribbon: β-1,3-glucan; purple ribbon: β−1,6-glucan; yellow branches: mannan; orange fibrils: chitin; gray: plasma membrane; green: protein; open blue circles: β-1,3-glucan branching from β−1,6-glucan backbone; black stars: retained peroxides. The orange-to-blue background gradient indicates increasing hydration. The orange dashed line marks the region that rigidifies upon hydroxide treatment, where hydroxides preferentially accumulate. **b** Schematic of a phosphate-transport-deficient mutant. Increased chitin content introduces additional rigid domains, causing normally flexible components (e.g., mannose sidechains and β−1,6-glucan) to become stiff, decreasing overall hydration and reducing the cell wall’s capacity to retain peroxides. The schematic illustrates polysaccharide organization and redistribution within the cell wall and does not represent the number or extent of covalent cross-links between chitin and glucans. Schematics reflect relative molecular composition but may not be strictly to scale.

High-resolution solid-state NMR thereby enables us to build an integrated structural model of both strains and to visualize how phosphate availability and ROS exposure reshape the cell-wall architecture. In the wildtype, the rigid core is organized primarily around β-1,3-glucan and a small portion of chitin, with additional contributions from a low content of β-1,6-glucan, and remains relatively hydrated and dynamic (Fig. 5a). In contrast, the rigid core of the phosphatetransport-deficient mutant is more heterogeneous, incorporating not only β-1,3-glucan and an increased amount of chitin, but also more α-1,2-mannose units and β-1,6-glucan, which become associated with these rigid components (Fig. 5b). In the mutant, some chitin microfibrils appear to reorganize into the outer wall domain, where they contact and rigidify mannan fibrils, similar to the remodeling mechanism observed in *C. albicans* following echinocandin treatment^50,96,97^. Although chitin synthesis occurs at the plasma membrane, the reorganized wall architecture in the mutant likely promotes progressive redistribution of chitin into mannan-associated outer wall regions during maturation. The reduced content of β-1,3-glucan and the increased abundance of chitin microfibrils lower hydration and restrict polymer mobility within the rigid fraction, resulting in a less accessible and more static architecture.

Upon H_2_O_2_ exposure, mobile α-1,2-mannose units and β-1,6-glucan in the wild type are recruited into the rigid scaffold (Fig. 5a). Preferential broadening of α-1,2-mannose resonances in the H_2_O_2_ induced rigidified domain indicates direct interaction between H_2_O_2_ and these polysaccharides. Oxidative damage involves both rigid and mobile fractions but occurs more extensively within the latter. In the mutant, H_2_O_2_ has limited access to the rigid core because of reduced hydration and increased packing, consistent with minimal broadening of rigid-core signals (Fig. 5b). Oxidative interactions occur primarily in the mobile layer, which rapidly loses NMR visibility in both strains, in agreement with molecular dynamics simulations showing that H_2_O_2_ is not intrinsically selective for any particular polymer. Together, these observations support a structural concept in which the wild-type wall intercepts ROS more effectively by engaging a substantial mobile carbohydrate fraction, a portion of the original rigid core, and polysaccharides that become rigid upon ROS exposure. In contrast, the mutant relies predominantly on its mobile carbohydrates, a significant fraction of which is already rigidified through interactions with β-1,3-glucan and chitin microfibrils and is therefore inaccessible to ROS. This limited capacity to redistribute and trap ROS contributes to the oxidative stress hypersensitivity of phosphatetransport mutants^13,25^.

The structural differences we observe are consistent with prior evidence that phosphate acquisition is closely linked to cell wall biosynthesis in *C. albicans*. Inorganic phosphate import supports intracellular phosphate pools and drives the production of nucleotide-sugar precursors required for β-glucan, chitin, and mannan synthesis^25,31^. Deletion of *PHO84* or disruption of the phosphate transport regulatory network increases susceptibility to cell-wall-active agents and compromises wall assembly, indicating that phosphate availability regulates wall architecture through its control of biosynthetic capacity^25,31^. Thus, the extensive remodeling we detect by solid-state NMR represents the structural outcome of phosphate-regulated biosynthetic pathways, extending beyond signaling and metabolic changes.

Our findings also reveal an unexpected biochemical intersection between phosphate metabolism and oxidative stress defense. Deletion of high-affinity phosphate transporters impairs intracellular phosphate homeostasis and attenuates TORC1 signaling, both of which are established regulators of oxidative adaptation and cell-wall integrity^13,25,31,53,98^. As a result, the phosphate transport mutant fails to initiate stress-induced redistribution of polysaccharides that is needed for reinforcing the rigid core of the wall. This indicates that phosphate sensing coordinates intracellular antioxidant responses with extracellular structural defenses. Reduced hydration and accessibility in the mutant further restrict peroxide binding and radical quenching. Thus, phosphate availability emerges as a central regulator that integrates nutrient sensing, redox homeostasis, and the mechanical architecture of the fungal cell wall.

Because Pho84-dependent phosphate acquisition and its downstream signaling pathways are conserved among fungal pathogens and required for virulence, targeting phosphate uptake or signaling could restrict the ability of pathogens to remodel and reinforce their cell wall during immune attack^53,99^. The pronounced vulnerability of the mannan-rich outer layer also identifies a structurally exposed domain that may be exploited pharmacologically or by host immune mechanisms. Together, our findings highlight phosphate homeostasis and wall architecture as actionable determinants of oxidative stress resilience and suggest promising therapeutic approaches that combine nutrient limitation, cell wall-active agents, and fungal-specific oxidative stress^100^ to safely and effectively eradicate *C. albicans* infections even in immunocompromised hosts.

## Methods

### Preparation of ^13^C-labeled yeast cells

The wild-type (JKC 915) and phosphate transport-deficient mutant (JKC 2830; *pho84Δ/Δ pho89Δ/Δ pho87Δ/Δ fgr2Δ/Δ*) of *C. albicans* (cells were received from Dr. Julia R. Köhler’s laboratory at Boston Children’s Hospital, MA, USA) were first pre-cultured in 50 mL of standard yeast nitrogen base (YNB) medium containing phosphate to ensure robust initial growth. The YNB medium was composed of 0.34 g (0.67%) YNB with ammonium sulfate and without amino acids, supplemented with 1 g of glucose (2% w/v) and 0.25 g of ammonium sulfate (5 g/L final concentration). A small inoculum (approximately the size of a pinhead) was transferred into 50 mL of this medium and incubated at 30 °C with agitation at 200 rpm for approximately 15 h. Cells from this culture were subsequently diluted 1:20 by transferring 2.5 mL into fresh 50 mL YNB medium lacking phosphate but containing 2% glucose and ammonium sulfate and incubated under the same shaking and temperature conditions for 24 h. This phosphate-deprivation step was repeated by pelleting cells via centrifugation at 1500 rpm for 15 min, decanting the supernatant, and resuspending the pellet in freshly autoclaved YNB medium without phosphate, containing 2% glucose and ammonium sulfate, followed by another 24 h incubation under identical conditions. For isotopic labeling, the cells were harvested again as above and transferred into 50 mL of freshly prepared YNB medium devoid of phosphate but supplemented with 2% uniformly labeled ^13^C glucose (1 g), 0.335 g of YNB without phosphate, and 3 mM of KH_2_PO_4_ (20.4 mg). The culture was incubated at 30 °C with shaking at 200 rpm for 24 h to allow for incorporation of ^13^C into cellular biomass. Cells were subsequently harvested and packed into 3.2-mm MAS rotors for solid-state NMR analysis.

### Preparation of ^13^C yeast cells for evaluating oxidative stress response via solid-state NMR

Prior to detailed NMR analysis, pilot experiments were conducted to determine the concentrations of H_2_O_2_ that diminish growth in the strains used here, without fully inhibiting it. For solid-state NMR analysis, *C. albicans* cells were grown in 0.67% yeast nitrogen base (YNB with phosphate), 2% glucose, and 5 g/L ammonium sulfate in 100 mL milli-Q water. Cells from YPD plates were inoculated into fresh medium to a starting OD_600_ of approximately 0.5 and grown overnight at 30 °C with shaking at 200 rpm. Cultures then were incubated at the same conditions until the OD_600_ of the control reached 1.0 (approximately 15 h), at which point all cultures were harvested by centrifugation at 1500 rpm in falcon tubes. Pellets were washed with Milli-Q water to remove residual medium.

For oxidative stress experiments, cells were cultured in defined medium supplemented with uniformly labeled ^13^C-glucose as the primary carbon source. The inclusion of 0.8 mM H_2_O_2_ (30% stock solution) allowed for controlled induction of peroxide stress under defined nutrient conditions. Two experimental media containing 0.8 mM H_2_O_2_ and a control without H_2_O_2_ were prepared. Following overnight pre-growth, cells were inoculated into each condition to a standardized OD_600_ of 0.2 and incubated at 30 °C with shaking. Cell density in the control condition was monitored, and all cultures were harvested when the control reached an OD_600_ of 0.4. Pellets were washed with Milli-Q water to remove extracellular metabolites and media components. Natively hydrated cells, approximately 45–50 mg per sample, were packed into 3.2-mm MAS rotors (HZ16916, Cortecnet) for subsequent solid-state NMR analysis.

### ^13^C solid-state NMR experiments

A variety of 1D and 2D ^13^C solid-state NMR experiments were conducted on two Bruker Avance Neo spectrometers at the MSU Max T. Rogers NMR Facility. High-resolution 1D and 2D spectra were acquired on the 800 MHz (18.8 T) spectrometer, while relaxation and water-editing experiments were performed on the 400 MHz (9.4 T) system. All spectra were acquired using 3.2-mm HCN probes at 15 kHz MAS and 293 K. ^13^C chemical shifts were externally referenced to the adamantane CH_2_ peak at 38.48 ppm on the tetramethylsilane (TMS) scale. Unless otherwise specified, typical radiofrequency field strengths were 80-100 kHz for ¹H decoupling, 62.5 kHz for ^1^H hard pulses, and 50-62.5 kHz for ^13^C. A summary of experimental parameters is provided in Supplementary Table 1. All spectra were processed using TopSpin 4.3.0, graphs were generated with OriginPro 9.0, and figures were prepared in Adobe Illustrator 2023 (v27.2.0).

1D ^13^C spectra were collected using different polarization schemes to selectively probe rigid and mobile components (Supplementary Fig. 2). Rigid components were identified using dipolar-coupling-based 1D ^1^H-^13^C cross-polarization (CP) with a 1 ms contact time, maintaining Hartmann-Hahn matching conditions at 62.5 kHz RF for ^1^H and 47.5 kHz RF for ^13^C channels.^101,102^. Mobile components were measured using ^13^C direct polarization (DP) with short 2 s recycle delays, while extending the delay to 35 s enabled quantitative detection of all carbon species in the same DP experiment. Highly mobile and solvent-exposed molecules were identified using J-coupling-based refocused Insensitive Nuclei Enhancement by Polarization Transfer (INEPT) experiments^103^.

Resonance assignments for rigid molecules were obtained from 2D ^13^C-^13^C CORD homonuclear correlation spectra with a 53 ms mixing time, which detect predominantly intramolecular cross-peaks^104^. For mobile components, 2D DP refocused J-INADEQUATE spectra were measured to determine through-bond connectivity, with each of the four τ delays optimized to 2.3 ms^105^. Assigned chemical shifts were compared with entries in the Complex Carbohydrate Magnetic Resonance Database (CCMRD) to validate carbohydrate identities^106^. The ^13^C chemical shifts of carbohydrates identified in *C. albicans* are listed in Supplementary Table 2.

Water accessibility of polysaccharides was assessed using a water-edited 2D ^13^C-^13^C correlation experiment^107,108^. The experiment began with ¹H excitation followed by a ^1^H-T_2_ filter (1.4 ms × 2 for the wildtype sample and 1.5 ms × 2 for the phosphate transport mutant), which suppressed approximately 95% of carbohydrate signals while retaining 75–80% of the water magnetization (Supplementary Fig. 4). The remaining water magnetization was transferred to nearby polysaccharides through a 4 ms ^1^H-^1^H mixing period and subsequently to ¹³C via a 1 ms ^1^H-^13^C CP step for high-resolution ^13^C detection. A 50 ms DARR mixing period was used for both the water-edited spectrum and a control 2D spectrum with full signal intensity. The ratio of intensities between the water-edited spectrum (S) and the control spectrum (S_0_) was calculated, with S/S_0_ values reflecting the relative hydration levels at individual carbon sites. All intensities were pre-normalized to the number of scans acquired for each spectrum.

The dynamics of cell wall polysaccharides were examined using ^13^C spin-lattice (T_1_) relaxation, which was measured using a series of z-filter delays ranging from 0.1 μs to 12 s (Supplementary Fig. 5 and Supplementary Table 3)^109^. For each well-resolved peak, the decrease in signal intensity with increasing z-filter time was quantified and fitted to a single-exponential equation to obtain the ^13^C-T_1_ relaxation constant for each carbon site. Peak intensities were pre-normalized by the number of scans. In addition, ^13^C-detected ^1^H-T_1ρ_ relaxation was measured using a Lee-Goldburg (LG) spin-lock sequence combined with LG-CP to suppress ^1^H spin diffusion during both the spin-lock and cross-polarization periods. This approach enabled site-specific analysis of ^1^H-T_1ρ_ relaxation through detection of the directly bonded ^13^C nuclei. The decay of peak intensity was fitted to a single-exponential function to obtain the ^1^H-T_1ρ_ relaxation time constant for each resolved site (Supplementary Fig. 5 and Supplementary Table 3).

The molecular composition was estimated by analyzing the intensities of distinct carbohydrate signals resolved in 2D ^13^C spectra. The composition of rigid components was estimated from 2D ^13^C-^13^C CORD spectra, and that of mobile molecules was evaluated using ^13^C DP refocused J-INADEQUATE spectra. Only well-resolved peaks were included in the analysis to mitigate uncertainty from spectral overlap. Relative polysaccharide abundances were determined by summing the integrated peak volumes for each polysaccharide and normalizing by the number of associated cross-peaks. The standard error for each polysaccharide was calculated as the standard deviation of the integrated peak volumes divided by the corresponding cross-peak count. The overall standard error was obtained as the square root of the sum of the squared individual standard errors. Percentage error was estimated by dividing each polysaccharide’s standard error by its mean integrated peak volume and then scaling by its relative abundance and its contribution to the total integrated volume.

### ^1^H-detected solid-state NMR experiments

The mobile polysaccharide components of the *C. albicans* cell wall were analyzed in both the wildtype and phosphate transport-deficient strains using scalar-coupling-based ^1^H-detected solid-state NMR experiments. All measurements were performed on a Bruker Avance Neo 800 MHz spectrometer equipped with a 3.2-mm HCN triple-resonance MAS probe, operating at a spinning frequency of 15 kHz. The feasibility of this experiment at slow MAS arises from the exclusive detection of mobile molecules, whose rapid dynamics sufficiently attenuate ^1^H-^1^H dipolar couplings to permit high-resolution spectra. ^13^C chemical shifts were calibrated to the TMS scale using the methylene resonance of adamantane at 38.48 ppm, and ^1^H chemical shifts were referenced to DSS (trimethylsilyl propanesulfonate) in D_2_O, with the DSS peak set to 0 ppm.

Proton-detected 2D ^1^H-^13^C *J*-hCcH TOCSY (total correlation spectroscopy)^67^ and 3D ^1^H-^13^C *J*-hCCH TOCSY experiments were performed using DIPSI-3 (decoupling in the presence of scalar interactions) mixing^68^ to enable efficient magnetization transfer through scalar couplings. In this pulse-sequence notation, capital letters denote nuclei whose frequencies are evolved (thus generating corresponding ^13^C or ^1^H dimensions in the spectra), while lowercase letters indicate nuclei for which frequency evolution is omitted. The 90° pulse lengths were 3.5 µs (71.4 kHz) for ^1^H and 5 µs (50 kHz) for ^13^C. SPINAL-64 (small phase incremental alternation with 64 steps) heteronuclear decoupling^69^ was applied during both t_1_ and t_2_ periods with a radiofrequency field strength of 71.4 kHz, and WALTZ-16 (wideband alternating-phase low-power technique for zero-residual splitting) decoupling^70^ was used during ¹H acquisition with a field strength of 17 kHz. Water suppression was implemented using the MISSISSIPPI pulse sequence (multiple intense solvent suppression intended for sensitive spectroscopic investigation of protonated proteins)^71^, with a pulse duration of 40 ms and a radiofrequency field strength of 26 kHz. DIPSI-3 mixing for homonuclear ^13^C-^13^C TOCSY transfers employed a 2 ms spin-lock pulse followed by a 25.5 ms mixing period, both applied at a radiofrequency field strength of 17 kHz.

For the wildtype sample, a 3D *J*-hCCH TOCSY dataset was collected using 128 complex points in both the t_1_ and t_2_ dimensions, 8 scans per increment, and a 2 s recycle delay, resulting in a total acquisition time of 77 h 16 min. A corresponding 2D *J*-hCcH TOCSY spectrum was also recorded using identical parameters, except with one t_1_ point, 512 t_2_ points, and 32 scans per increment, yielding a total duration of 2 h 25 min. All spectra were acquired using States-TPPI for quadrature detection in the indirect dimensions^72^. The assigned ^13^C and ^1^H chemical shifts are provided in Supplementary Table 4. Complete acquisition parameters are summarized in Supplementary Table 5.

### Molecular dynamics simulations

Molecular dynamics simulations were performed to assess the propensity of hydrogen peroxide molecules to interact with specific glycans or their linkages, as illustrated in Supplementary Fig. 17. Four glycan chains were examined. A short chitin chain was constructed using ChitinBuilder^110^. A mannose chain and two mixed glucan chains with distinct linkage patterns were generated using psfgen in VMD (version 1.9.4a58)^111^, and subsequently minimized with LongBondEliminator^112^ to remove potential ring penetrations. Each glycan was solvated in a TIP3P water box with 6 nm side lengths^113^. To introduce oxidative conditions, 67 water molecules were replaced with hydrogen peroxide molecules, resulting in an effective hydrogen peroxide concentration of approximately 0.55 M. This high concentration allows for sufficient sampling in a limited simulation length, and the atomistic representation allowed us to quantify specific interactions at the nanoscale.

Each system was simulated five times for 1000 ns using NAMD 3.0.1^114^ and the CHARMM36 force field for carbohydrates and chitin^115,116^. The simulations were performed at 298 K and 1 atm, maintained by a Langevin thermostat^117^ and isotropic barostat^118^. Each simulation step was 2 fs long, enabled by the SETTLE algorithm^119^. Long range electrostatics was handled by particle mesh Ewald^120^ summation with a 1.2 Å grid spacing. As is typical for CHARMM36, we use a 12 Å cutoff, smoothly switching at 10 Å.

The primary analysis for these trajectories was to analyze the interaction propensity for specific moieties on the glycans with hydrogen peroxide. We do this through evaluating a contact number that smoothly rather than harshly cuts off if atoms move further apart as shown in Eq. 1:

1 $$C={\sum}_{i,j}1/1+{e}^{5\left({d}_{{ij}}-4{{{{\AA }}}}\right)}$$

This contact definition has been used previously to quantify intermolecular interactions in plant biopolymers^121–123^. We evaluate this contact number over just heavy-atom pairs, which means that even a single contact is quite significant. This contact metric varies quickly over time (Supplementary Fig. 17), and so more specific atomic contacts as in Fig. 4 are taken over the full trajectory.

### Transmission electron microscopy and analysis of cell wall thickness

Cell wall thickness in *C. albicans* was evaluated by TEM under phosphate-depleted conditions and following oxidative stress (0.8 mM H_2_O_2_). Cells were fixed in 2.5% glutaraldehyde and 2% paraformaldehyde prepared in 0.1 M sodium cacodylate buffer. To preserve cellular morphology and minimize processing-induced shrinkage, samples were immobilized in 2% agarose prior to further preparation. Post-fixation was performed with osmium tetroxide, followed by dehydration through a graded acetone series and sequential infiltration with epoxy resin-acetone mixtures (25:75, 50:50, and 75:25). After overnight incubation in the 75:25 resin-acetone mixture, samples were transferred to 100% epoxy resin for 48 h with multiple resin exchanges before polymerization at 70 °C. Ultrathin sections were prepared using a Leica Microsystems LEICA EM UC7 ultramicrotome, stained with 1% uranyl acetate and lead acetate, and mounted on carbon-coated grids for imaging. Cell wall thickness was quantified from 100 independent measurements for each strain and condition using ImageJ (v1.54 g). Statistical comparisons were performed using an unpaired two-tailed Student’s *t*-test in Microsoft Excel 365, and distribution profiles were visualized as color-coded violin plots generated with OriginPro 2021.

### Alcian blue staining assay method

The standard curve and Alcian blue binding were determined as in Hobson et al.^124^. Briefly, harvested cells were washed twice with 0.9% NaCl and adjusted to an OD_600_ of 1.0 in 0.9% NaCl. Cells were collected by centrifugation at 5000 × g for 5 min at room temperature. Following removal of the supernatant, cell pellets were resuspended in 1 mL of 30 µg/mL Alcian blue prepared in 0.02 N HCl and incubated at room temperature for 10 min. Cells were then pelleted again by centrifugation at 5000 × g for 5 min, and unbound Alcian blue remaining in the supernatant was quantified. Cell pellets were photographed immediately after supernatant removal.

### Reporting summary

Further information on research design is available in the Nature Portfolio Reporting Summary linked to this article.

## Supplementary information

Supplementary Information

Reporting Summary

Transparent Peer Review file

## Source data

Source Data

## Data Availability

All relevant data that support the findings of this study are provided in the article and Supplementary Information. All the original ssNMR data files have been deposited in the Zenodo repository and can be freely accessed (https://doi.org/10.5281/zenodo.21690324; accessible at https://zenodo.org/records/21690324). MD input data and analysis are available for download, and selected outputs are also available from Zenodo (https://doi.org/10.5281/zenodo.19411396; accessible at https://doi.org/10.5281/zenodo.19411396). Source Data are provided as a source data file. Source data are provided with this paper.
