# Supplementary material for: Oxidative stress-responsive cell wall remodeling depends on phosphate in Candida albicans
Source: Nat Commun. 2026 Sep 10;17:10676. doi: 10.1038/s41467-026-77594-x (PMC13654929; doi:10.1038/s41467-026-77594-x)
Supplement: Supplementary file 1 [file 41467_2026_77594_MOESM1_ESM.pdf]

Supplementary Information

Oxidative Stress-Responsive Cell Wall Remodeling Depends on Phosphate in *Candida albicans*

Anand Jacob[1], Wanjun Qi[2], Jayasubba Reddy Yarava[1], Murtaza Barkarar[3,4], Alicia Withrow[5], Aswath Karai[1], Josh V. Vermaas[3,4], Julia R. Köhler[2]*, Tuo Wang[1]*

[1] Department of Chemistry, Michigan State University, East Lansing, MI 48824, USA

[2] Division of Infectious Diseases, Boston Children's Hospital/Harvard Medical School, Boston, Massachusetts, USA

[3] MSU-DOE Plant Research Laboratory, East Lansing, MI, USA

[4] Department of Biochemistry and Molecular Biology, Michigan State University, East Lansing, MI, USA

[5]Center for Advanced Microscopy, Michigan State University, East Lansing, MI 48824, USA

Corresponding authors: wangtuo1@msu.edu, julia.koehler@childrens.harvard.edu

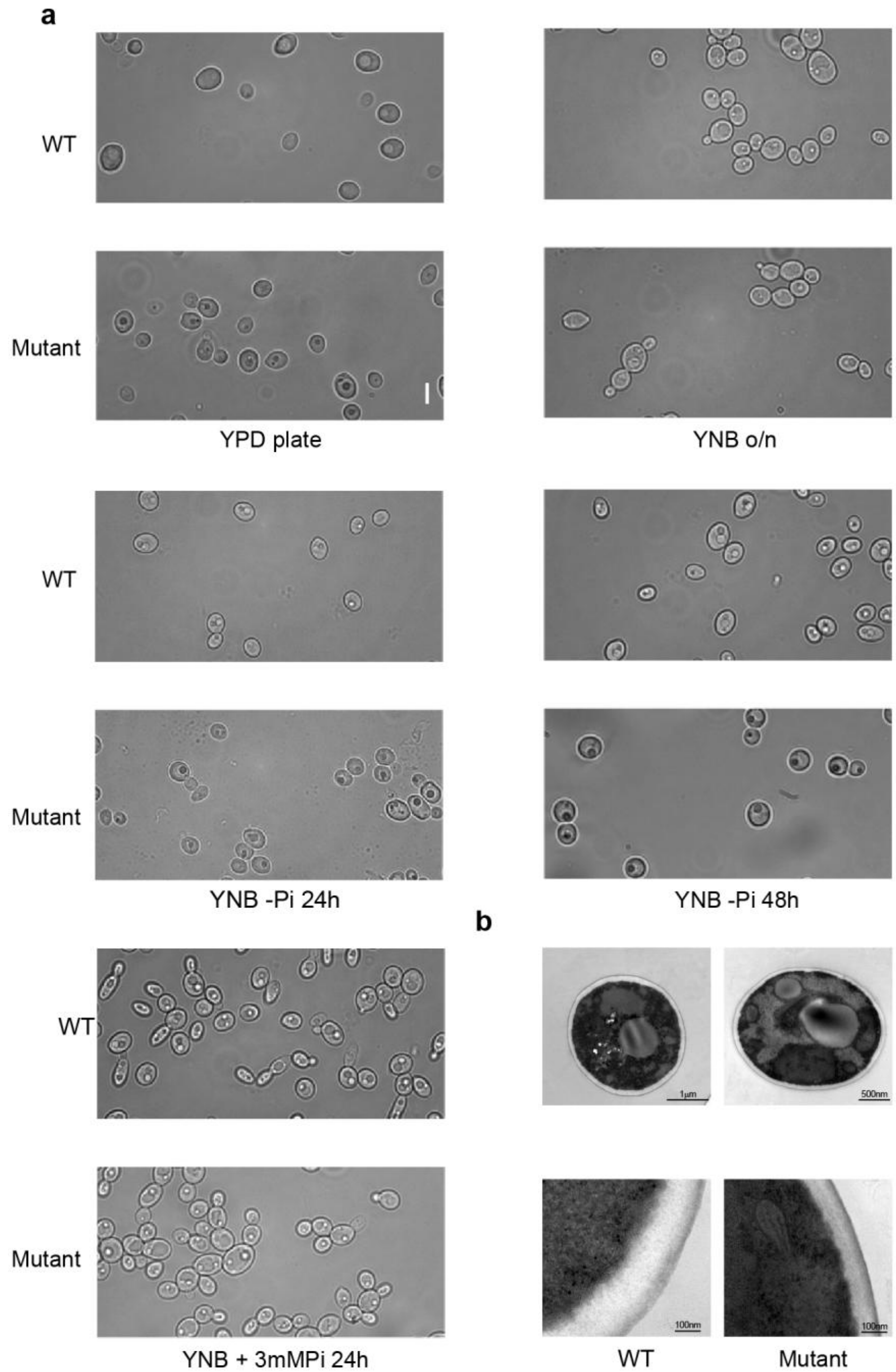

Supplementary Figure 1. Wildtype and phosphate transport mutant cell morphologies did not substantially diverge during phosphate starvation. (**a**) Micrographs of cells harvested from YPD plates, after overnight (o/n) preculture in standard yeast nitrogen base medium (YNB), after 24 and 48 h of phosphate starvation (YNB -Pi) and after 24 h in YNB containing 3 mM Pi (YNB + 3mM Pi). Images are representative of 4 biological replicates ($n = 4$). Scale bar represent 5 µm. Image brightness and contrast were adjusted using Microsoft PowerPoint and Affinity Designer. Strains are wildtype and the phosphate transporter mutant. (**b**) TEM images of wildtype and phosphate transport mutant of *C. albicans*.

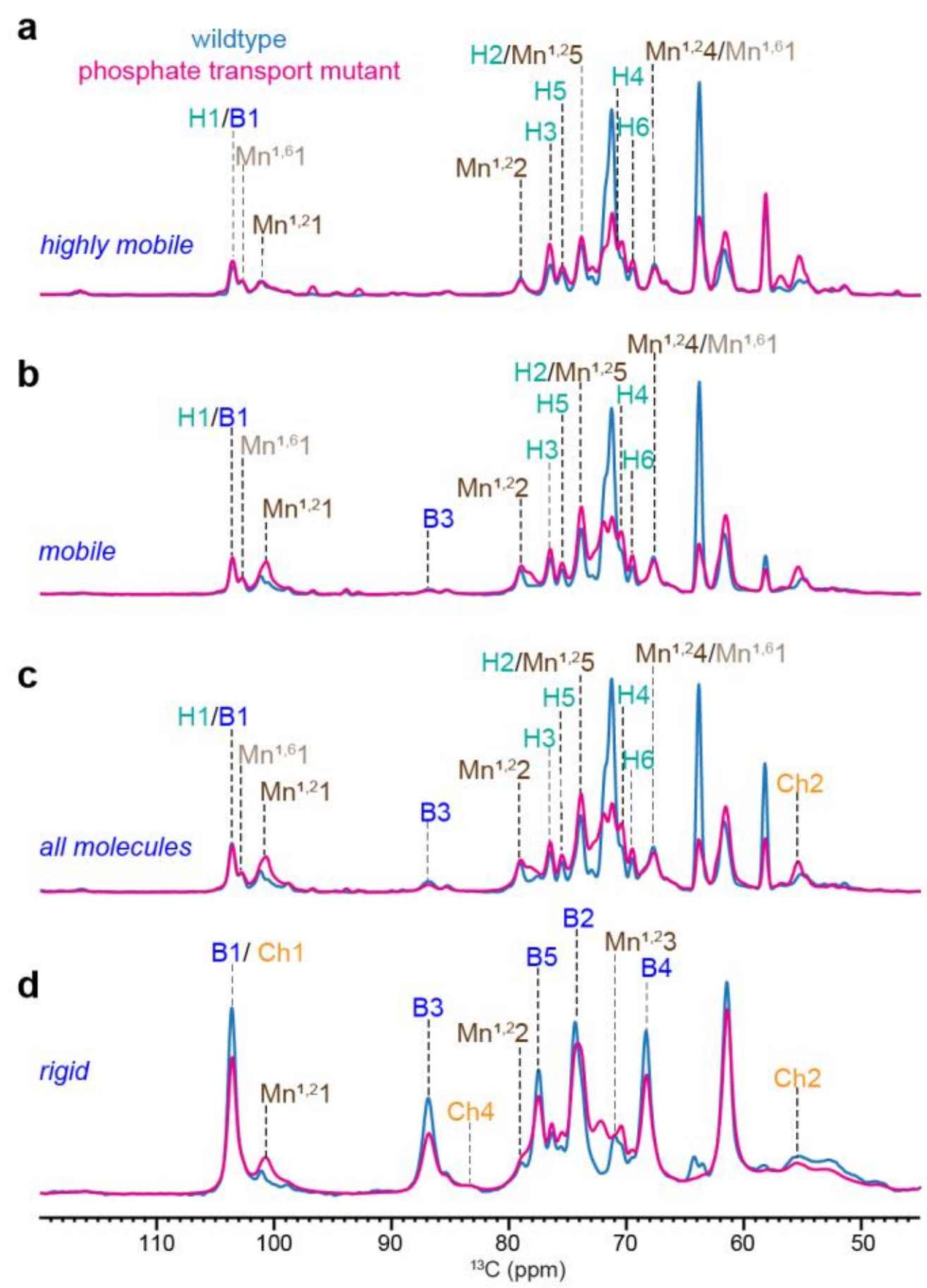

Supplementary Figure 2. Dynamical gradient of polysaccharides in *Candida albicans* cell walls. From top to bottom are four sets of 1D ^{13}C spectra measured with (**a**) refocused INEPT experiment for probing the most dynamic molecules. (**b**) DP spectra with short recycle delay of 2 s for selection of mobile components. (**c**) DP with long recycle delays (35 s) for quantitative detection of all molecules. (**d**) CP for selecting rigid polysaccharides. The spectra of *C. albicans* wildtype and phosphate transport mutant are shown in blue and magenta, respectively. For example, the $Mn^{1,2}1$ peak at 101 ppm annotates the carbon 1 of α-1,2-mannose ($Mn^{1,2}1$).

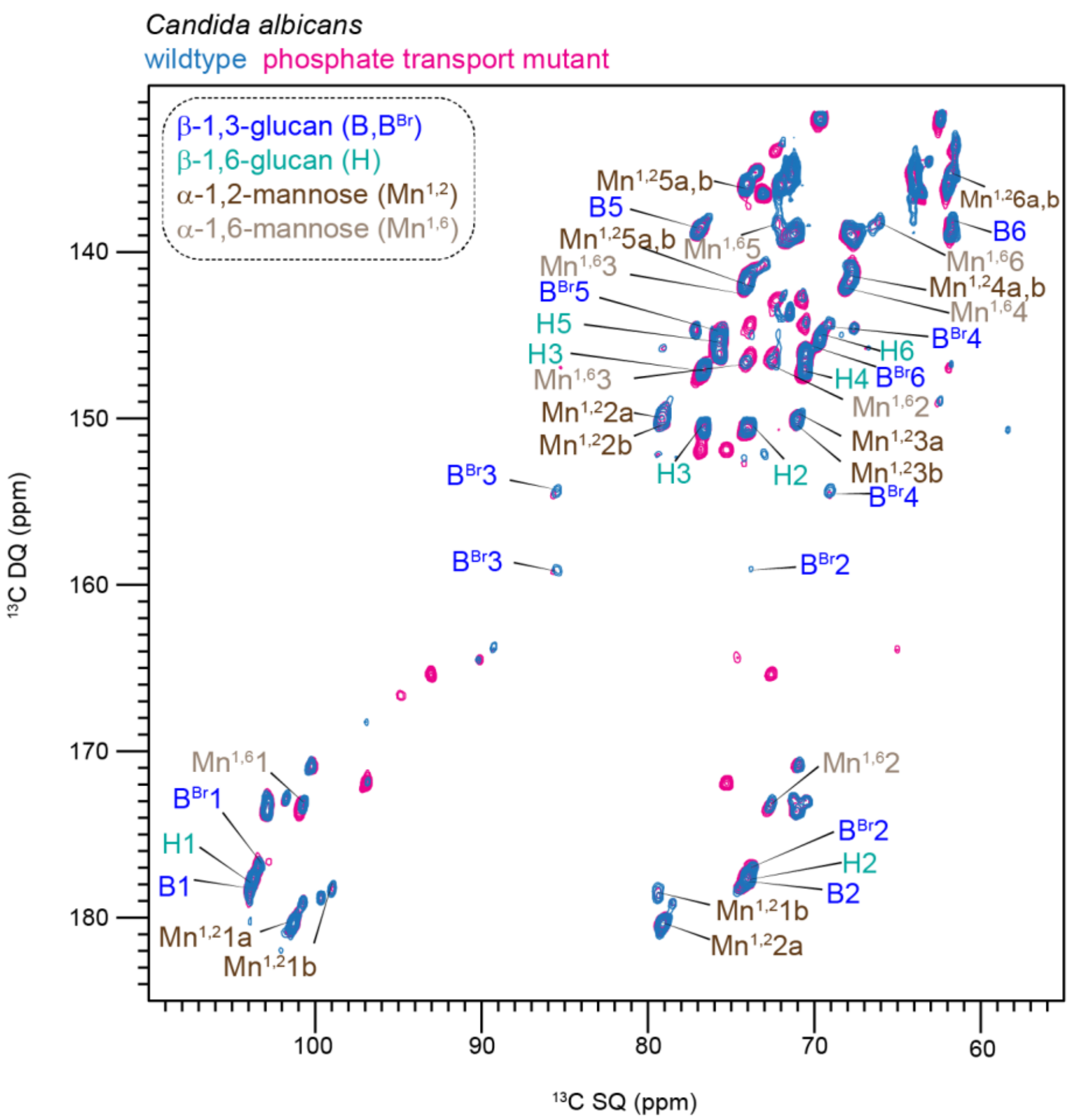

Supplementary Figure 3. 2D ^{13}C DP refocused J-INADEQUATE spectrum of *C. albicans* samples. Signals are detected for linear β-1,3-glucan (B), linear β-1,6-glucan (H), β-1,3,6-glucopyranose unit (B^{Br}) of the branched glucan, as well as α-1,6-mannan ($Mn^{1,6}$) and α-1,2-mannose ($Mn^{1,2}$) residues of mannan. The changes noted are mainly from soluble, small molecules rather than these cell wall polymers.

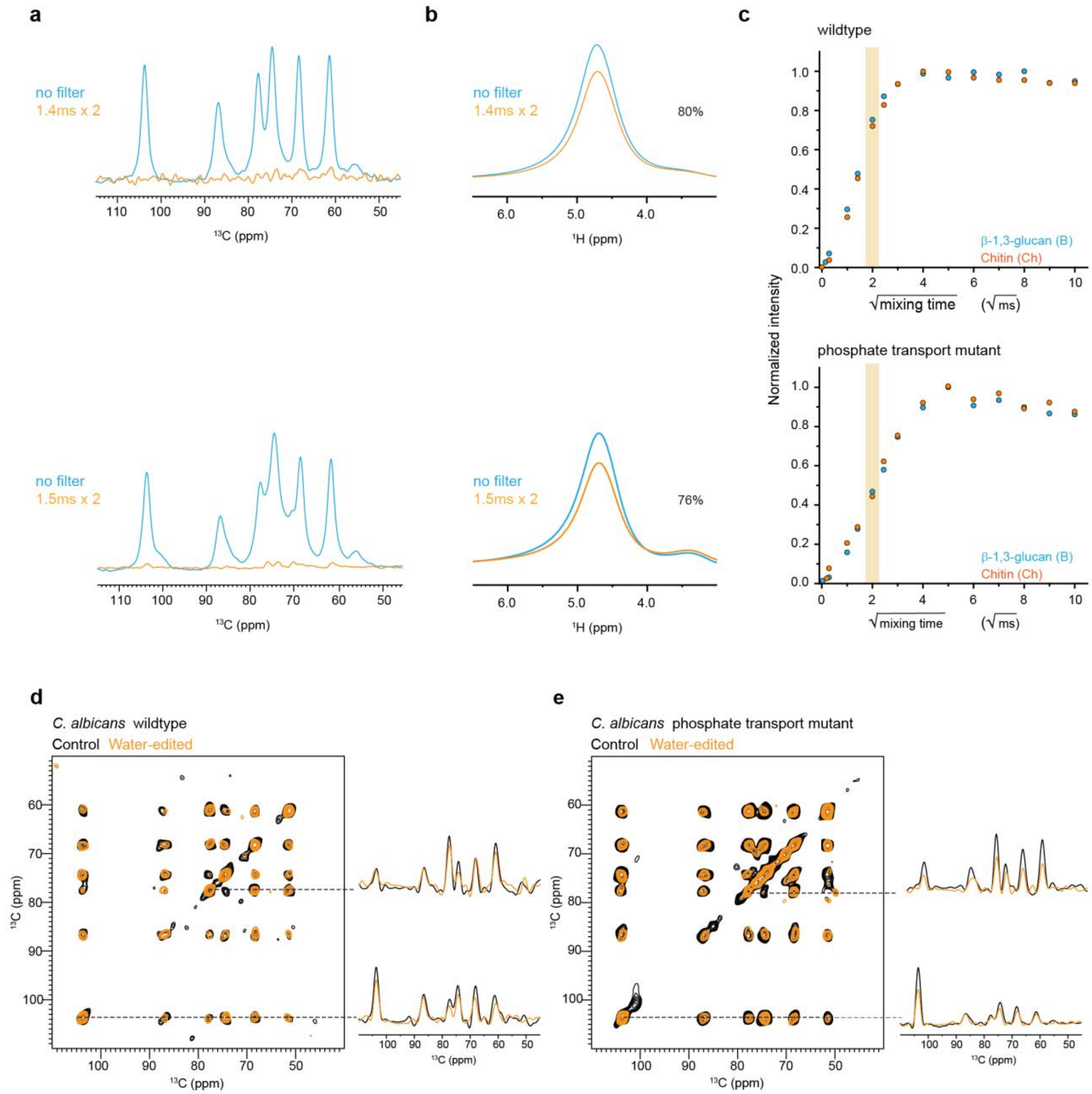

Supplementary Figure 4. Water-edited spectra of *C. albicans* to access polymer hydration. (**a**) ^{1}H-T_2 filtered (orange) and control (cyan) ^{13}C spectra are shown for wildtype (top) and phosphate transport mutant (bottom) *C. albicans* samples. No spin diffusion was applied. Approximately 95% of carbohydrate ^{13}C signals were removed by the ^{1}H-T_2 filter. (**b**) ^{1}H-T_2 filtered (orange) and control (cyan) ^{1}H NMR spectra, with 80% and 76% of water signal retained for wildtype and phosphate transport mutant respectively after the ^{1}H-T_2 filter. (**c**) Representative water-to-polysaccharide ^{1}H spin diffusion buildup curves are shown for wildtype (top) and phosphate transport mutant (bottom). Overlay of 2D water-edited (orange) and control (black) ^{13}C-^{13}C correlation spectra of (**d**) wildtype sample and (**e**) phosphate transport mutant. Representative 1D slices extracted from the 2D ^{13}C-^{13}C correlation spectra are shown for each sample. All spectra were measured on a 400 MHz spectrometer at 10 kHz MAS.

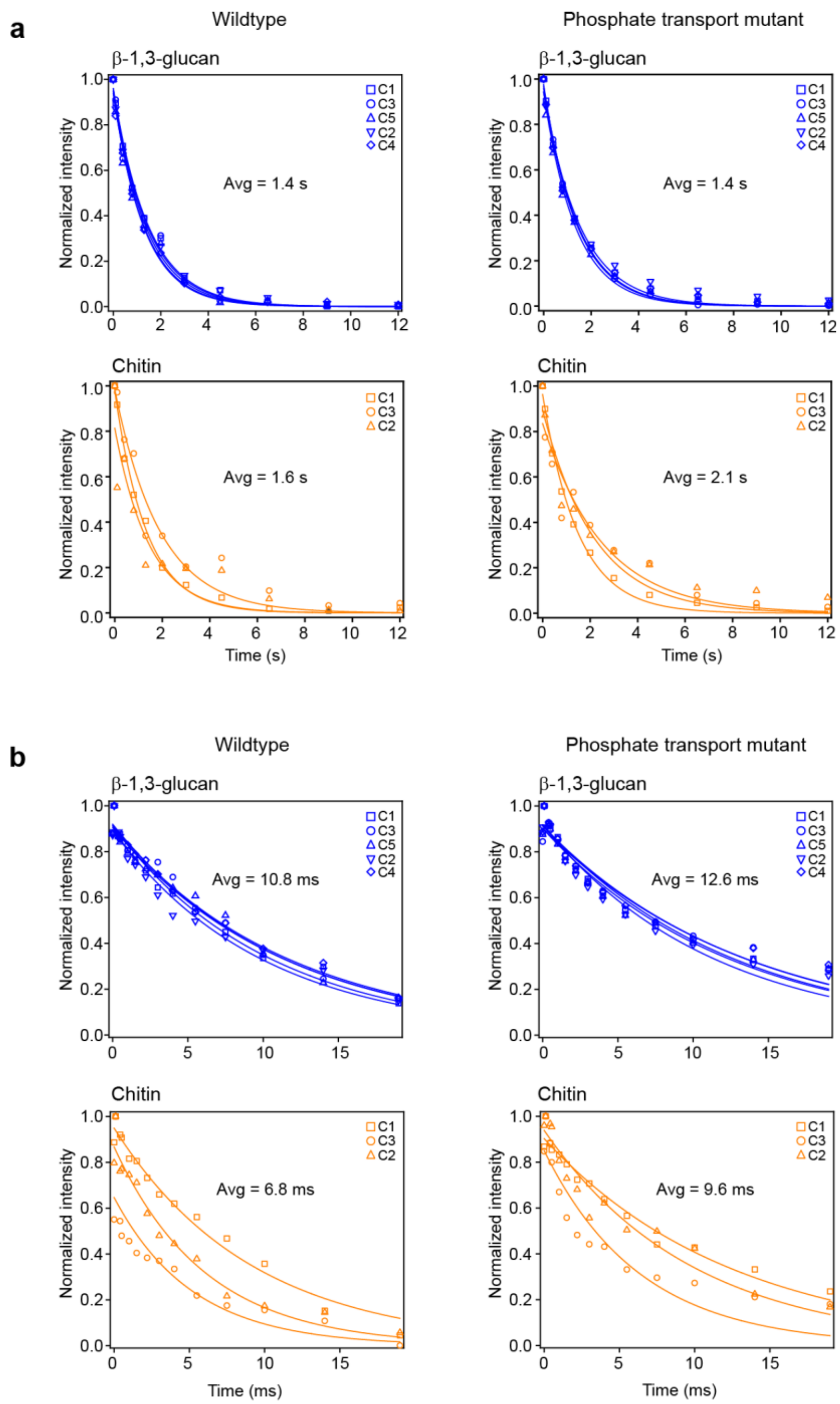

Supplementary Figure 5. **Relaxation curves of polysaccharides in *C. albicans* cell wall.** The relaxation decay curves are shown separately for (**a**) ^{13}C T1 and (**b**) ^{1}H T1ρ of the *C. albicans* wildtype and phosphate transport mutant.

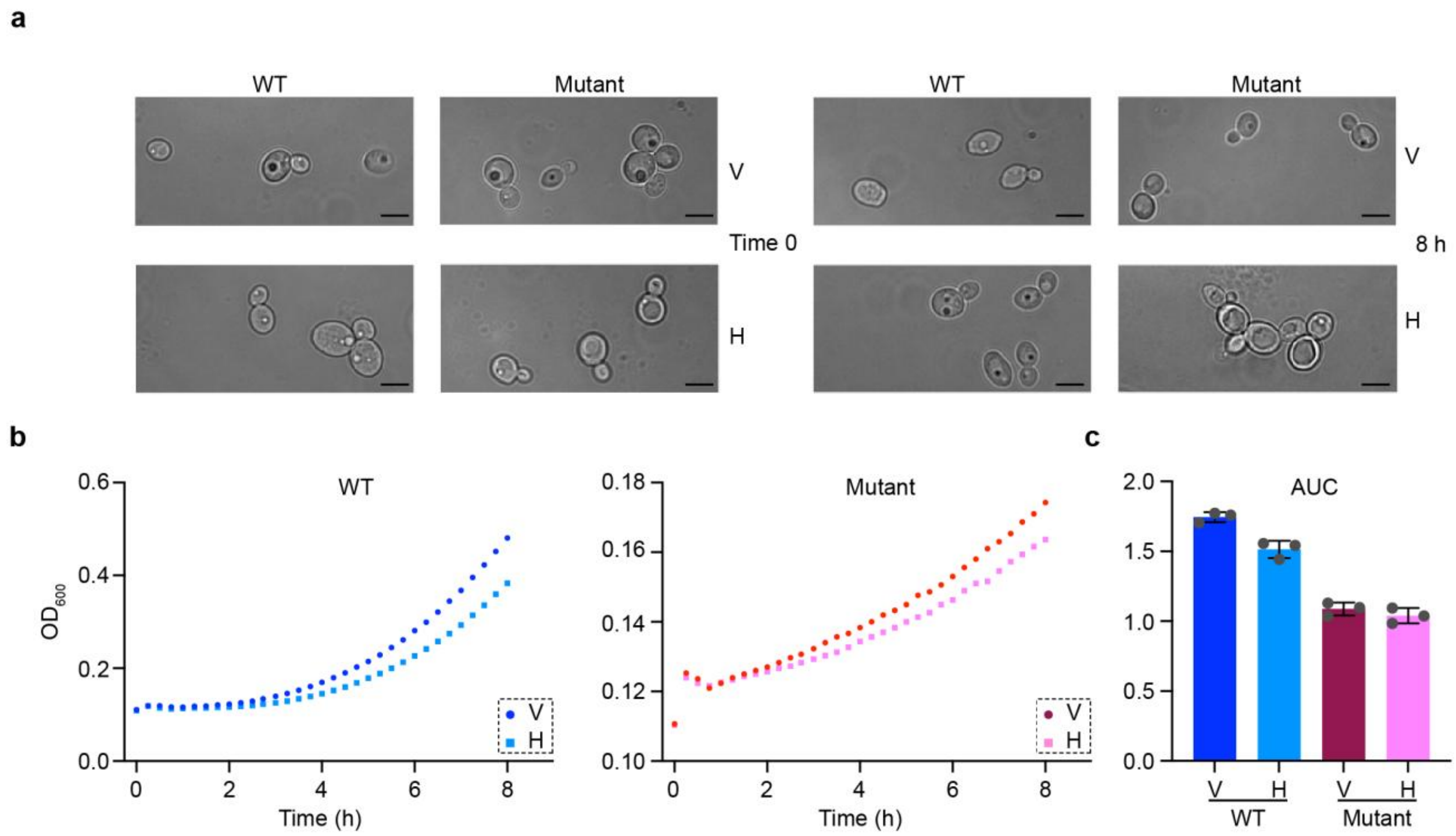

Supplementary Figure 6. **Minimal morphological divergence and minor growth inhibition of wildtype and phosphate transport mutant cells following exposure to 0.8 mM H_2O_2.** (**a**) Micrographs of cells inoculated into standard YNB containing vehicle (V, H_2O) or 0.8 mM H_2O_2 (H) at the beginning (time 0) and at the end (8 h) of H_2O_2 exposure were formaldehyde fixed, washed in phosphate-buffered saline (PBS) before microscopy. Images are representative of 2 biological replicates; bar = 5 µm. Image brightness and contrast were adjusted using Microsoft PowerPoint and Affinity Designer. Strains are wildtype (WT, JKC915); phosphate transporter mutant (Mutant, JKC 2830, *pho84Δ/Δ pho89Δ/Δ pho87Δ/Δ fgr2Δ/Δ*). (**b**) Wildtype and phosphate transporter mutant cells were pre-grown in YNB overnight, then inoculated into YNB containing vehicle (V, H_2O) or 0.8 mM H_2O_2 (H). OD_{600} readings were monitored every 15 min up to 8 h using a BioTek Synergy2 plate reader. Growth curve, representative of $n = 3$ biological replicates, shows mean of 3 technical replicates. (**c**) Total area under the growth curves in panel A were calculated and plotted using Prism 9 GraphPad (GraphPad Software, Inc., CA, USA). Plots show the mean with s.d. of 3 biological replicates ($n = 3$). Source data are provided as a source data file.

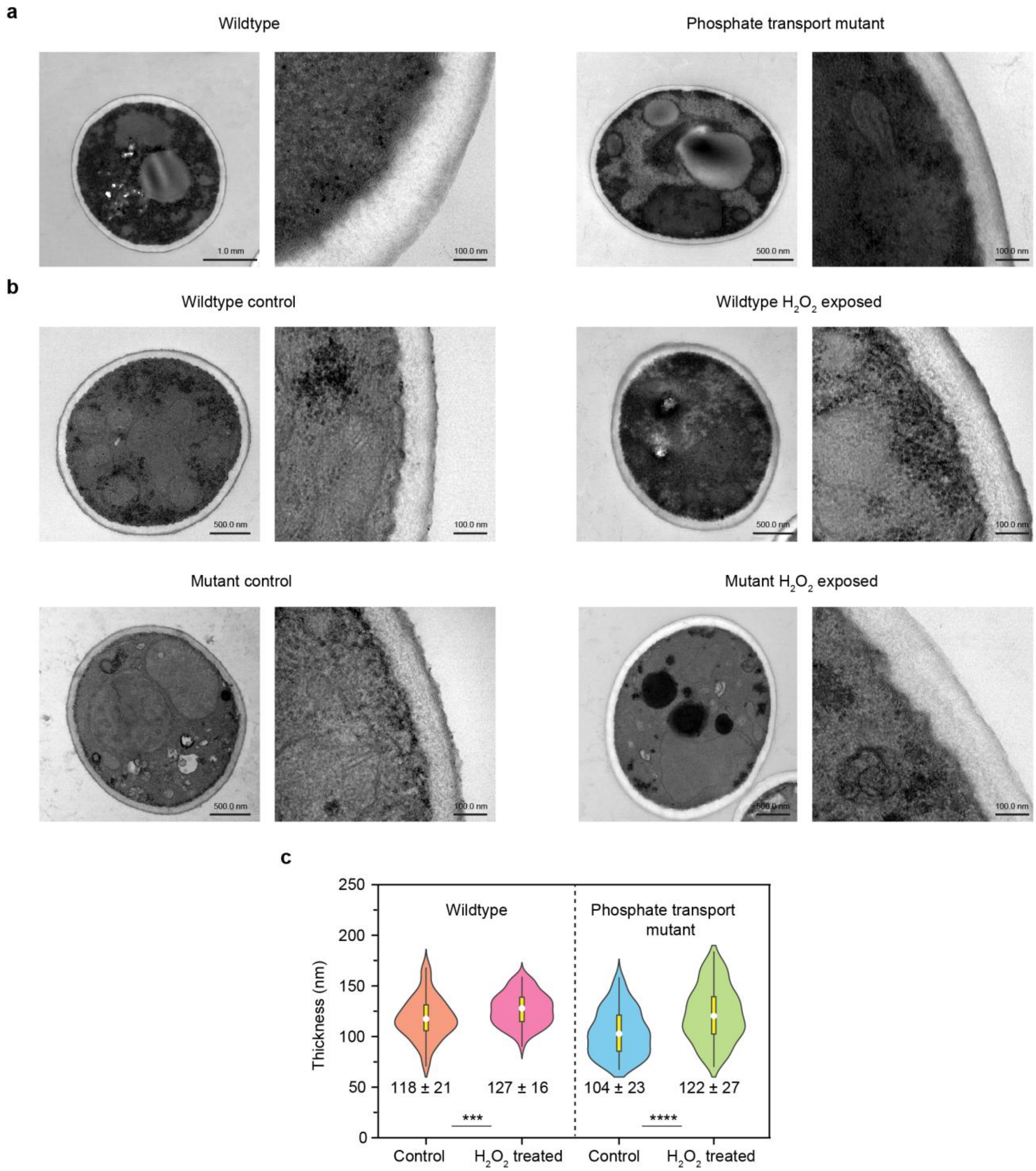

Supplementary Figure 7. TEM analysis of cell wall thickness. (**a**) TEM images of wildtype and phosphate transport mutant and (**b**) under oxidative stress. (**c**) Comparison of cell wall thickness in wildtype (orange) and phosphate transport mutant (blue) of *C. albicans* under oxidative stress. The mean ± s.d. are provided. $n = 10$ for each sample. Black rectangles denote the interquartile range (IQR) from 25th to 75th percentile. White circles represent the mean of the dataset, and black vertical lines correspond to 1.5IQR. Statistical analysis was performed using a two-tailed Welch's *t*-test comparison between the two samples. Statistically significant: ***p-value<0.001 (9.29×10^{-4}), ****p-value < 0.0001 (8.89×10^{-7}).

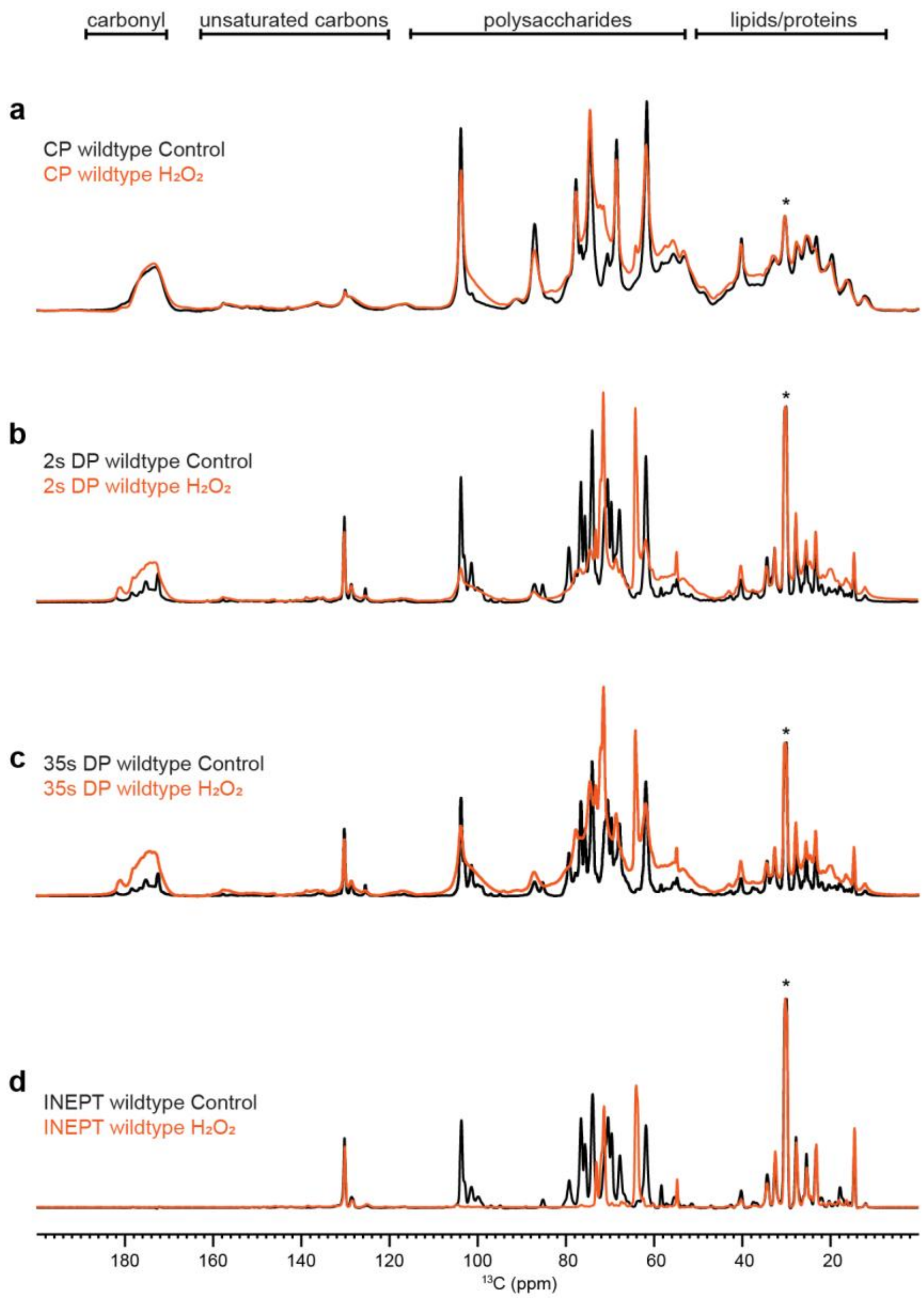

Supplementary Figure 8. Full 1D ^{13}C spectra of wildtype *C. albicans* cell wall after H_2O_2 treatment. From top to bottom are four sets of 1D ^{13}C spectra measured with (**a**) CP for selecting rigid polysaccharides. (**b**) DP spectra with a short recycle of 2s for selection of mobile components. (**c**) DP with long recycle delays (35s) for quantitative detection of all molecules. (**d**) Refocused INEPT experiments for probing the most dynamic molecules. The spectra for the wildtype of *C. albicans* control and H_2O_2 treated is shown in black and orange color, respectively.

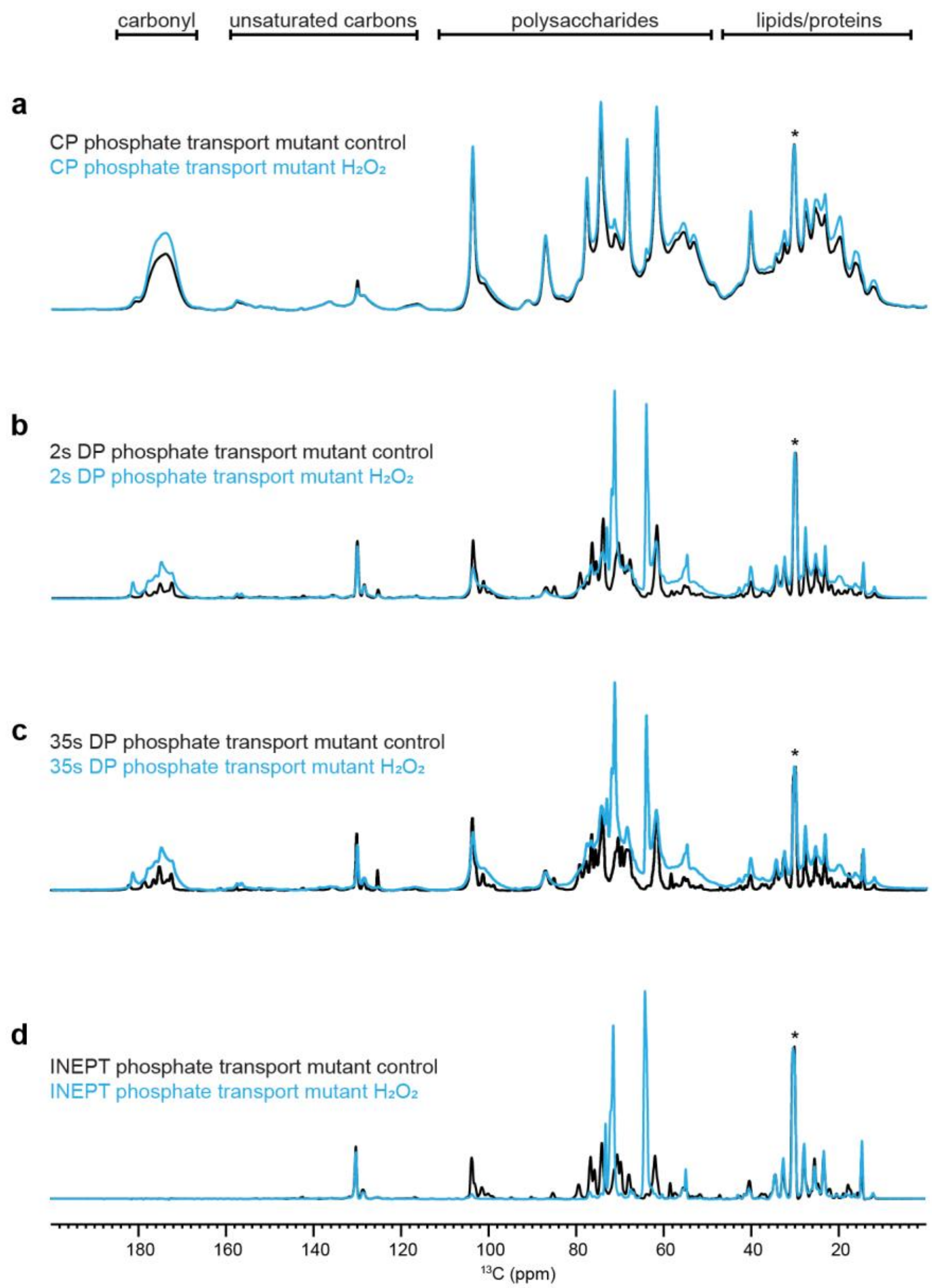

Supplementary Figure 9. Full 1D ^{13}C spectra of phosphate transport mutant *C. albicans* cell wall after H_2O_2 treatment. From top to bottom are four sets of 1D ^{13}C spectra measured with (**a**) CP for selecting rigid polysaccharides. (**b**) DP spectra with a short recycle of 2s for selection of mobile components. (**c**) DP with long recycle delays (35s) for quantitative detection of all molecules. (**d**) Refocused INEPT experiments for probing the most dynamic molecules. The spectra for the phosphate transport mutant of *C. albicans* control and H_2O_2 treated is shown in black and cyan color, respectively.

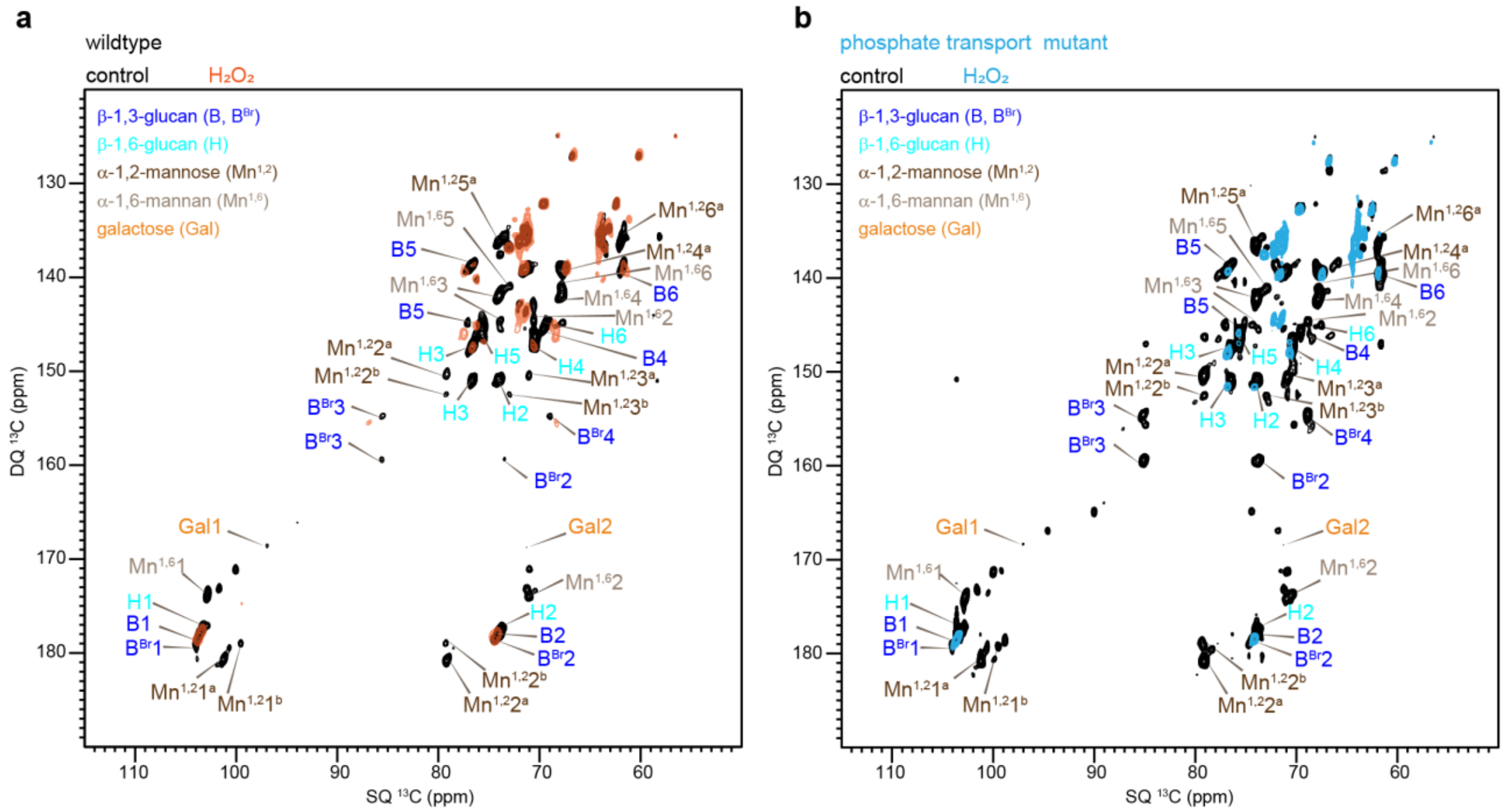

Supplementary Figure 10. **Full carbohydrate region of 2D ^{13}C DP refocused J-INADEQUATE spectra.** Mobile molecules are detected in (**a**) wildtype and (**b**) phosphate transport mutant cells under control or H_2O_2-treated conditions.

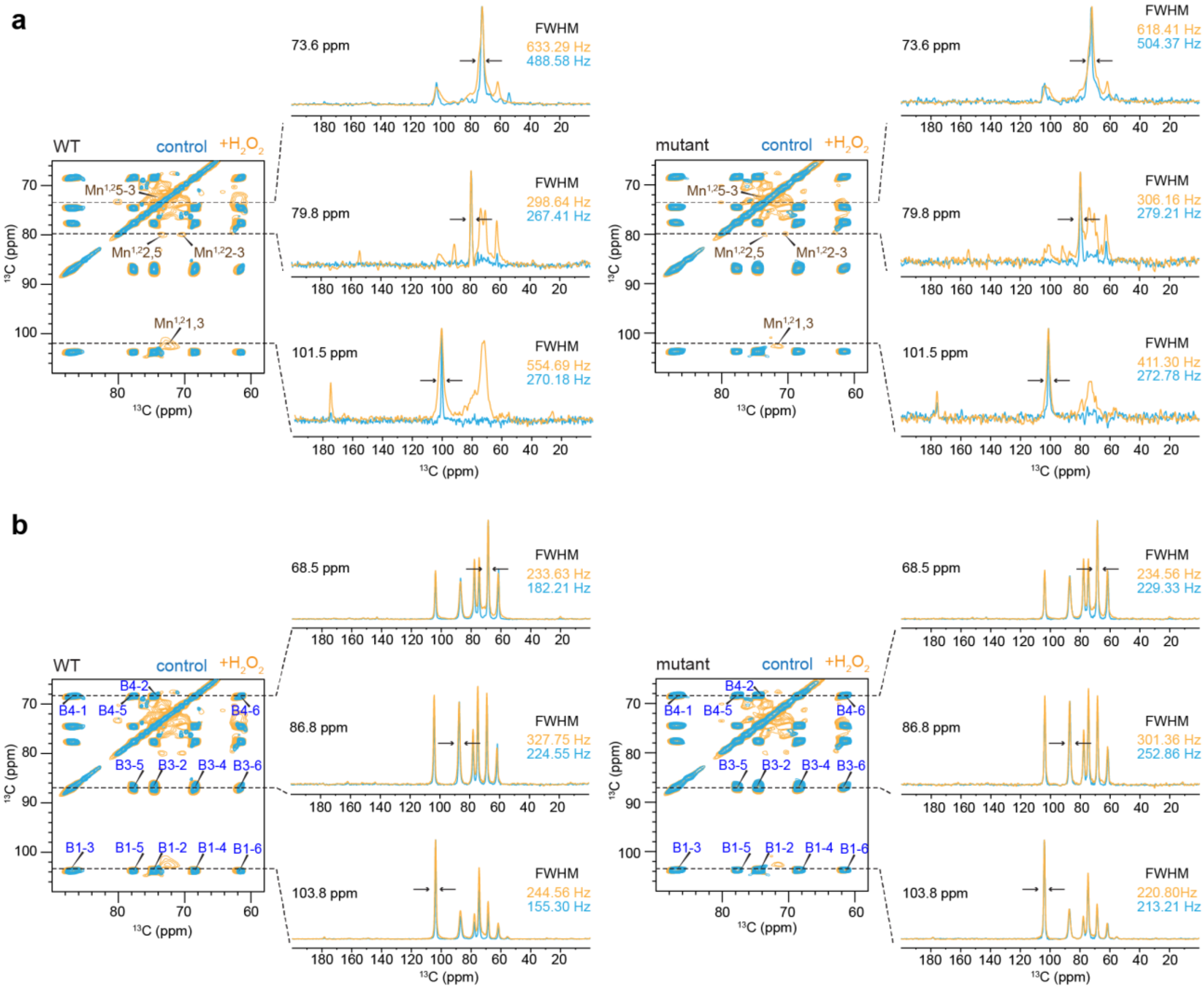

Supplementary Figure 11. 1D cross sections extracted from the 2D ^{13}C-^{13}C CORD spectra. Selected 1D cross sections corresponding to (**a**) α-1,2-mannose and (**b**) β-1,3-glucan, comparing control and H_2O_2 treated wildtype and phosphate transport mutant cell wall. All 1D spectra were processed identically using a GM window function (LB = -10 Hz, GB = 0.05) and 2D ^{13}C-^{13}C CORD processed using QSINE (SSB =4, LB = -10 Hz, GB = 0.05). The cross-sectional analysis enables direct comparison of line-broadening and intensity variations between wildtype and phosphate transport mutant, which is arising from PRE effect, and resolves H_2O_2 induced signal broadening in rigid polysaccharide domains.

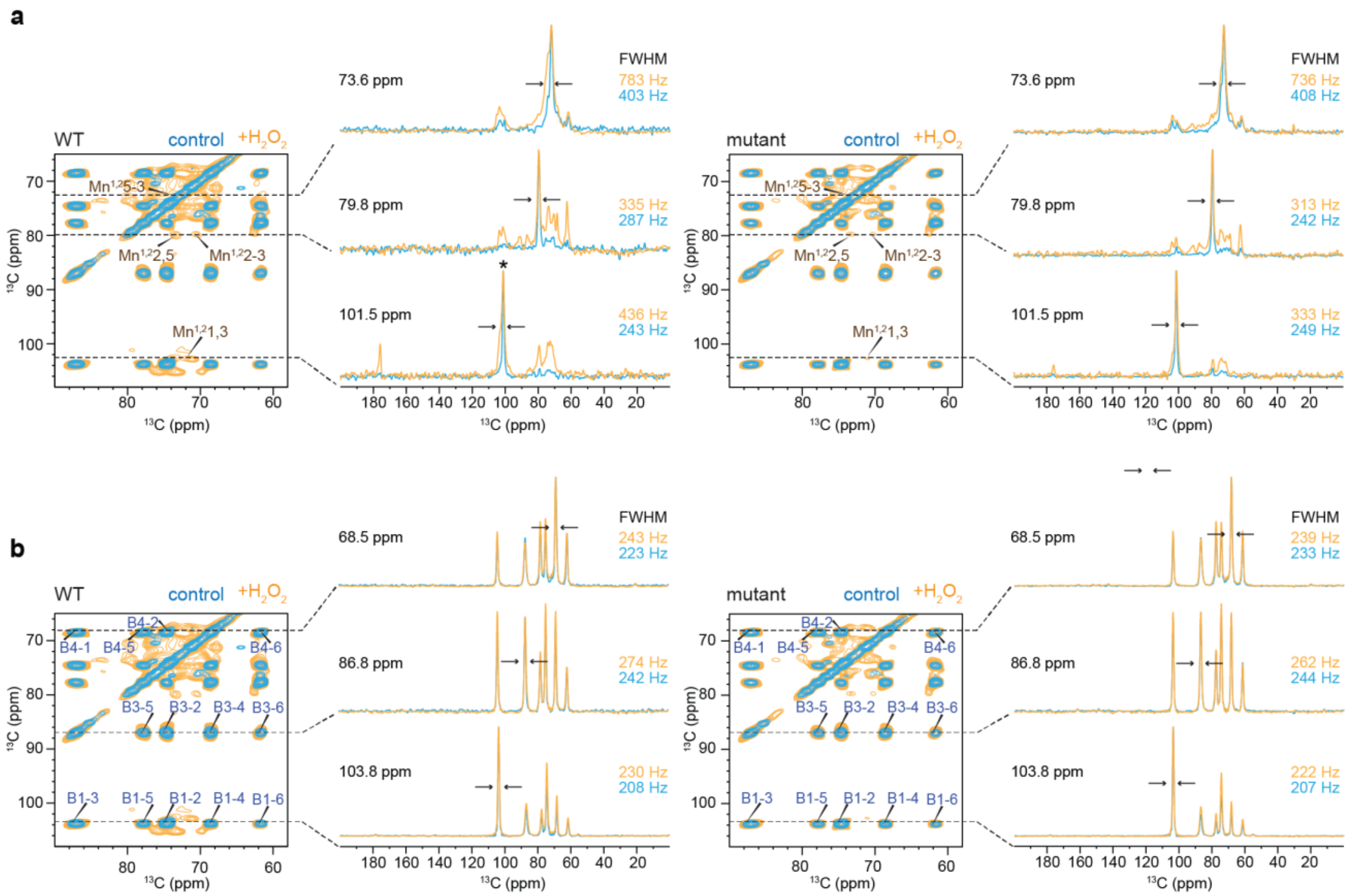

Supplementary Figure 12. Linewidth differences in a second biologically independent sample. Selected 1D cross sections extracted from the 2D ^{13}C-^{13}C CORD spectra of a second biologically independent preparation, showing (**a**) α-1,2-mannose and (**b**) β-1,3-glucan resonances from control and H_2O_2 treated wildtype and phosphate transport mutant cell walls. All 1D spectra were processed identically using a GM window function (LB = -10 Hz, GB = 0.05) and 2D ^{13}C-^{13}C CORD processed using QSINE (SSB =3, LB = -10 Hz, GB = 0.05). Cross-sectional analysis of biological replicate demonstrates reproducible linewidth broadening between wildtype and phosphate transport mutant samples, reflecting PRE associated effects and H_2O_2 induced perturbations in rigid polysaccharide domains.

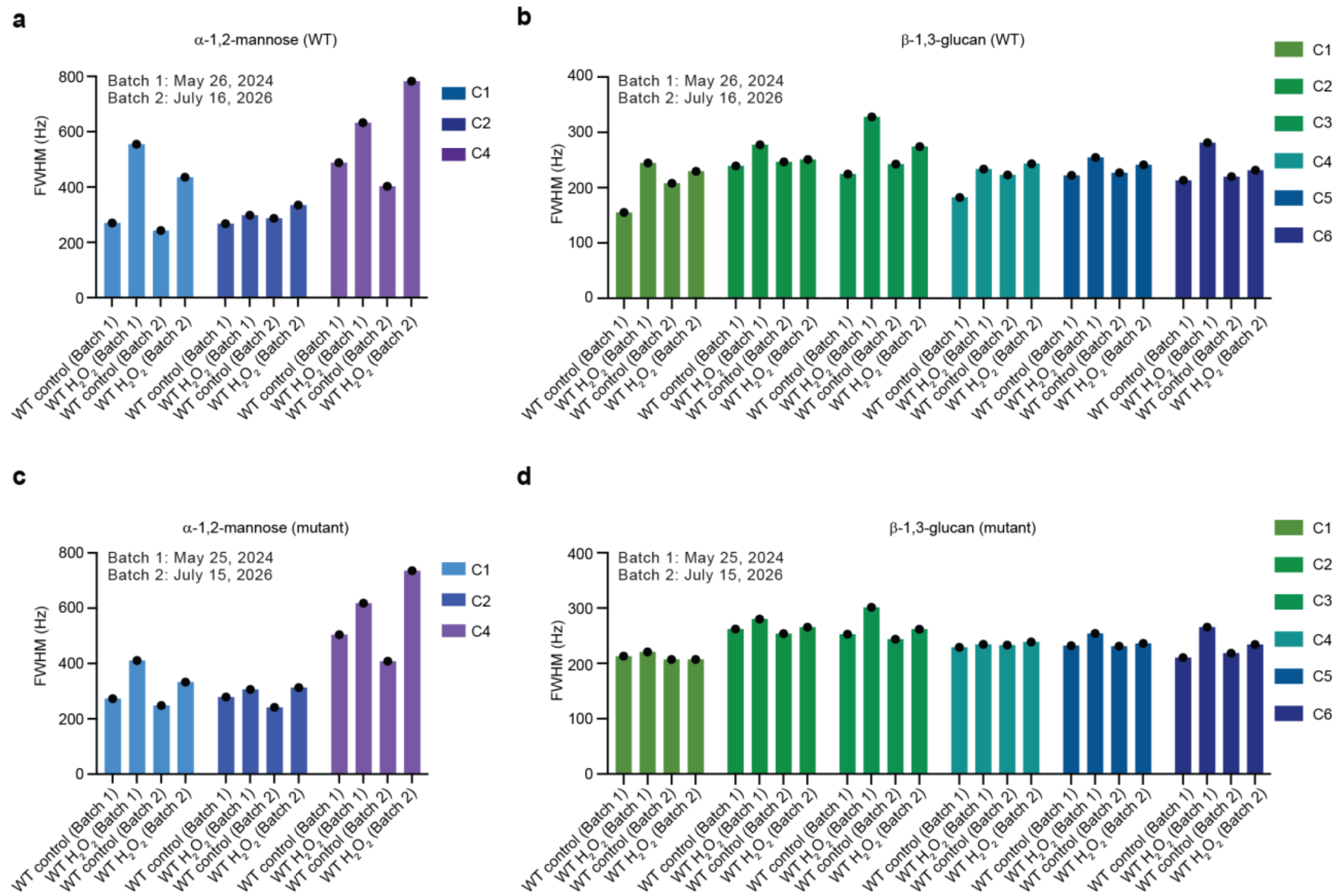

Supplementary Figure 13. ^{13}C Resonance linewidths in control and H_2O_2-treated cell wall. FWHM values were extracted from individual carbon resonances in 2D ^{13}C-^{13}C CORD spectrum and compared across all resolved carbon sites between two biologically independent sample batches (Batch 1 and Batch 2). Each bar represents a single FWHM value from one batch (n = 1) with the corresponding data point overlaid on each bar. Comparisons were performed for wildtype and phosphate transport mutant cell walls under control and H_2O_2 treated conditions. The two batches showed consistent linewidth patterns, including H_2O_2 induced broadening of rigid polysaccharide resonances and reproducible differences in the extent of line broadening between wildtype and phosphate transport mutant cell walls, with wildtype showing greater broadening relative to their respective control spectra.

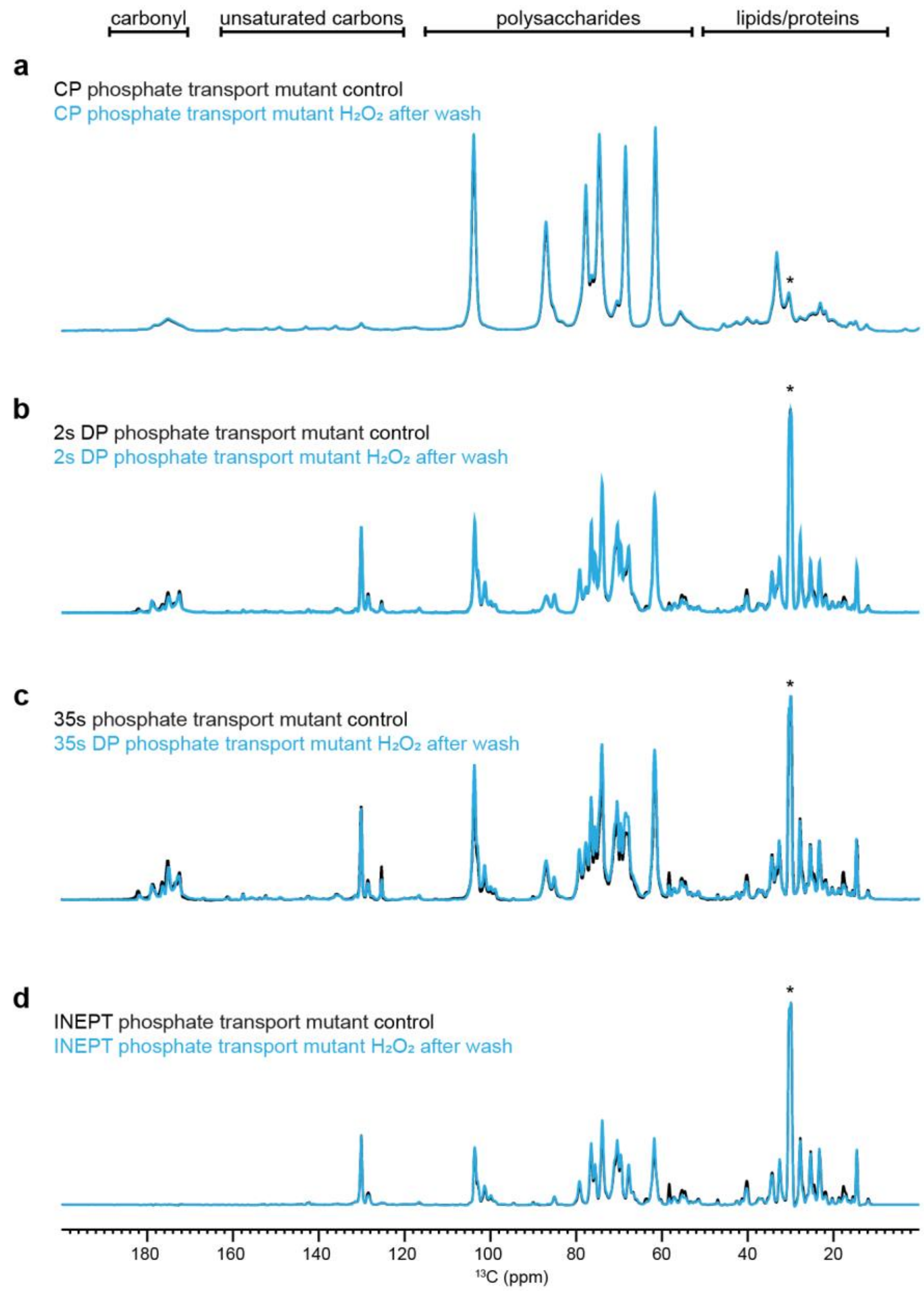

Supplementary Figure 14. 1D ^{13}C spectra of H_2O_2-treated phosphate transport mutant *C. albicans* cell wall after washing. From top to bottom are four sets of 1D ^{13}C spectra measured with (**a**) CP for selecting rigid polysaccharides. (**b**) DP spectra with a short recycle of 2s for selection of mobile components. (**c**) DP with long recycle delays (35s) for quantitative detection of all molecules. (**d**) Refocused INEPT experiments for probing the most dynamic molecules. The spectra for the phosphate transport mutant of *C. albicans* control and washed after H_2O_2 treatment is shown in black and cyan color, respectively.

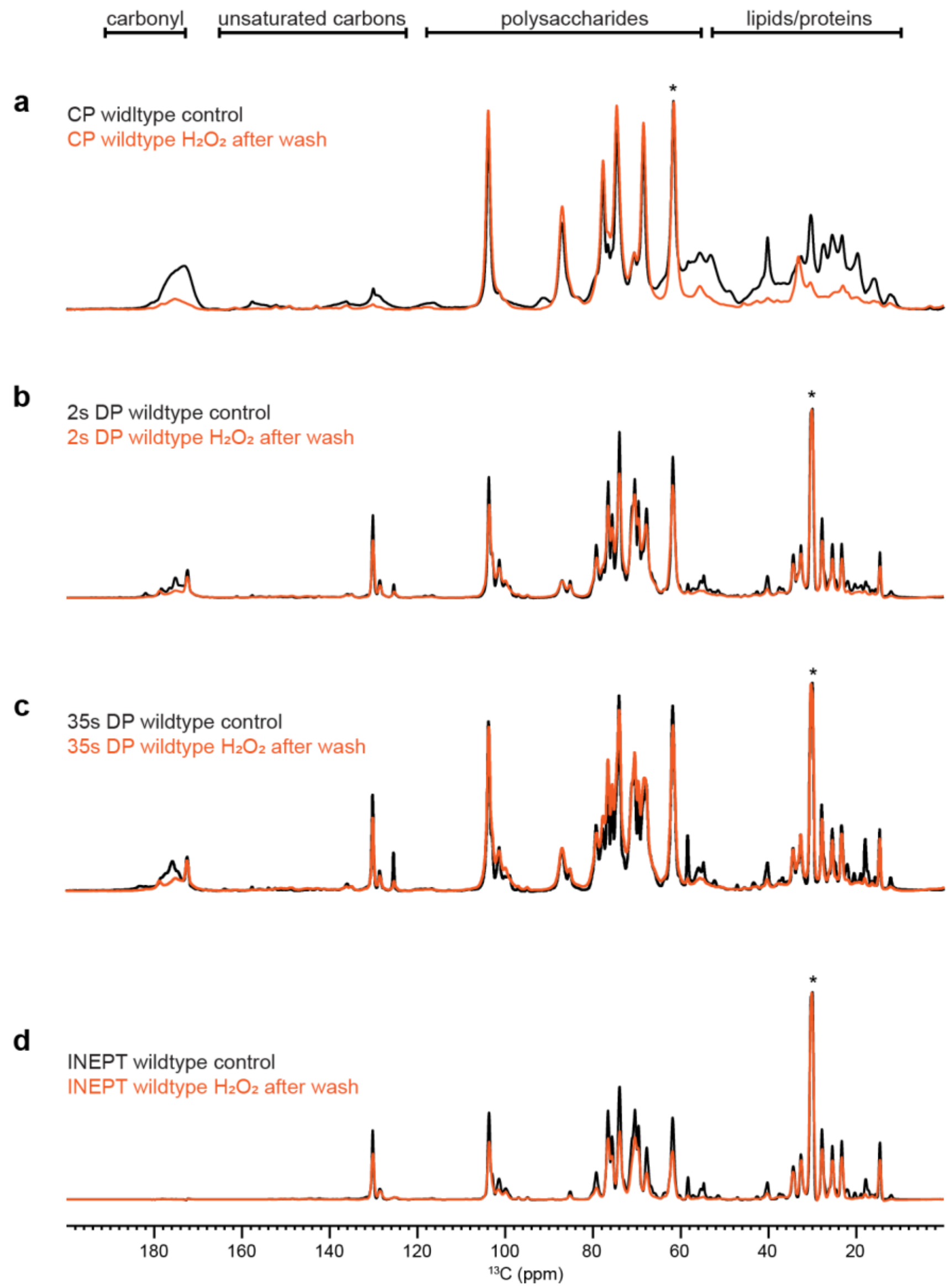

Supplementary Figure 15. 1D ^{13}C spectra of H_2O_2-treated wildtype cell wall after washing. From top to bottom are four sets of 1D ^{13}C spectra measured with (**a**) CP for selecting rigid polysaccharides. (**b**) DP spectra with a short recycle of 2s for selection of mobile components. (**c**) DP with long recycle delays (35s) for quantitative detection of all molecules. (**d**) Refocused INEPT experiments for probing the most dynamic molecules. The spectra for the wildtype of *C. albicans* control and washed after H_2O_2 treatment is shown in black and orange color, respectively.

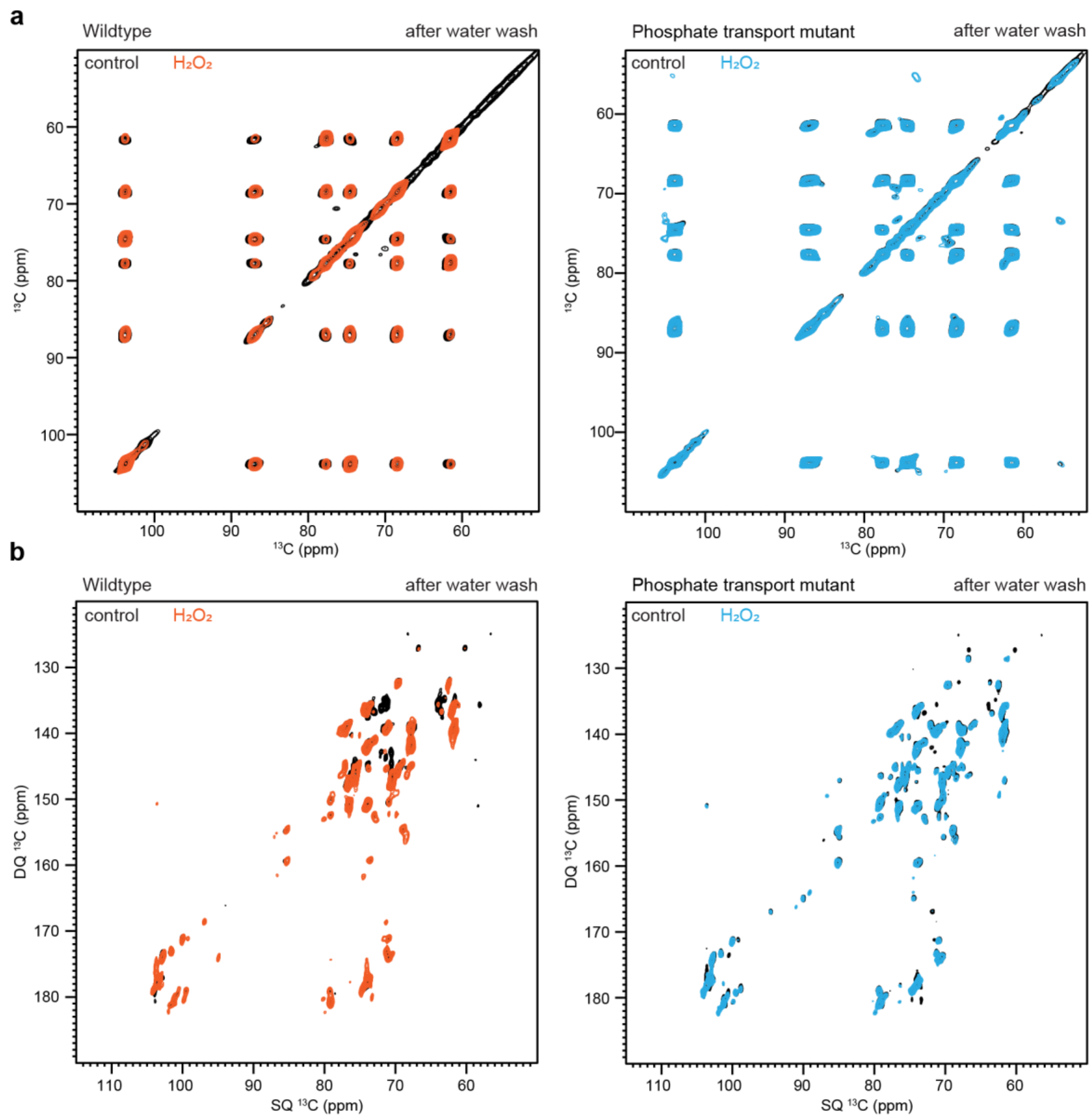

Supplementary Figure 16. 2D ^{13}C spectra of H_2O_2-treated *C. albicans* cell wall after washing. (**a**) 2D ^{13}C-^{13}C CORD correlation spectra which detects the rigid polysaccharide components in control and H_2O_2 treated wildtype and phosphate transport mutant cell wall. (**b**) ^{13}C DP refocused J-INADEQUATE spectra which detects the mobile polysaccharide components in control and H_2O_2 treated wildtype and phosphate transport mutant cell wall. The spectra show wildtype *C. albicans* control and H_2O_2 washed samples in black and orange, respectively and phosphate transport mutant control and H_2O_2 washed samples in black and cyan, respectively.

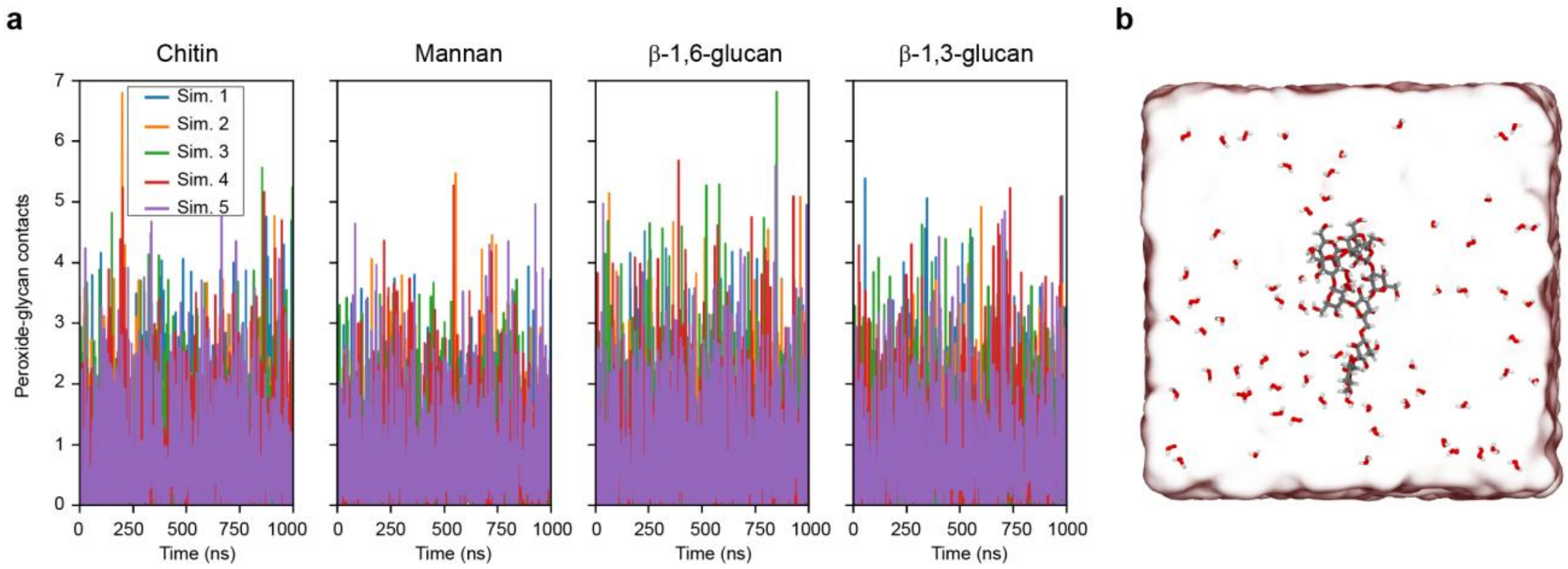

Supplementary Figure 17. **Molecular simulation contact timeseries and snapshot.** (**a**) Timeseries for peroxide-glycan contacts across the four systems of interest. There is little substantial variation between replicates. The contacts are sporadic across the timeseries, and so we use the full trajectory to make the measurements for **Figure 4**. (**b**) A molecular system, featuring a small glycan chain (gray carbons, red oxygens, white hydrogens) within a water box (transparent surface) and a relatively high concentration of hydrogen peroxide molecules, shown here in a licorice representation.

Supplementary Table 1. Solid-state NMR experiments and parameters. To be quantitative, direct pulse (DP) experiments with 35 s long recycling delay were used. cross-polarization (CP), most rigid molecules. With DP and a shorter recycling delay of 2 seconds, suppress the rigid molecules from the spectra, and with Insensitive Nuclei Enhanced by Polarization Transfer (INEPT) the most mobile molecules were selected. For 2D ^{13}C-^{13}C correlation experiments allowed to resolve rigid intramolecular peaks. 2D DQ-SQ, DP J-INADEQAUTE and CP INADEQUATE spectra were used to detect through-bond correlations. The experimental parameters include the ^{1}H Larmor frequency, total experiment time (t), recycle delay (d1), number of scans (NS), The number of points for the direct (td2) and indirect (td1) dimensions, the acquisition time of the direct dimension (aq2) and the evolution time of indirect dimension (aq1), spectral width (sw1 and sw2), mixing time (t_m), increment delay (IN_F) and T filter times. * Indicates the water-polysaccharide spin diffusion. The processing parameters include the window function and associated parameters.

	Acquisition parameters													Processing parameters	
Experiment	$\omega_{0,\,1H}$ (MHz)	t (h)	d1 (s)	NS	td2	td1	aq2 (ms)	aq1 (ms)	sw2 (ppm)	sw1 (ppm)	t_m (ms)	IN_F (μs)	T filters	Window function	Parameter
1D ^{13}C CP	800	0.5	2.0	1024	3200		16.0		496.8		1.0			GM	LB-10, GB0.05
1D ^{13}C DP	800	0.2	2.0	256	3200		16.0		496.8					GM	LB-10, GB0.05
1D ^{13}C DP	800	2.5	35.0	256	3200		16.0		496.8					GM	LB-10, GB0.05
1D ^{13}C INEPT	800	1.5	3.5	1024	3200		16.0		496.8					GM	LB-10, GB0.05
1D ^{13}C T1	400	3.5	2.0	512	2000		16.0		623.3				T_1(10^{-3}-12 s)	GM	LB-10, GB0.05
1D ^{1}H T1ρ	400	4.0	2.0	512	2000		16.0		623.3				SL (10^{-3}-19 ms)	GM	LB-10, GB0.05
2D ^{13}C-^{13}C CORD	800	11.0	2.0	32	2800	600	14.0	7.5	496.8	198.7	53.0	25		QSINE	SSB 3.5
2D ^{13}C-DP INADEQUATE	800	6.0	2.0	16	2800	680	14.0	7.4	496.8	225.8		22			
2D ^{13}C-^{13}C Water-edited	400	8.0	2.0	64	2000	220	16.0	5.4	623.3	199.4	10^{-4} /50*	50	T_2 10^{-4} ms	QSINE	SSB 3.5

Supplementary Table 2. ^{13}C chemical shifts of biomolecules in *C. albicans* cell wall at ambient temperature. Superscripts are used to denote different allomorphs. Not applicable (/). Unidentified (-). Branched (Br). Reducing end (O).

Carbohydrate		C1	C2	C3	C4	C5	C6	CO	CH_3	N	Experiment	References
β-1,3-glucan		103.8	74.6	86.8	68.5	77.7	61.6	/	/	/	^{13}C-^{13}C CORD	Shim *et al*. 2007[1] Fairweather *et al*. 2004[2] Saito *et al*. 1979[3]
Chitin		103.9	55.2	73.3	83.3	75.8	60.9	175.1	22.8	/		
β-1,3-6-glucan (B^{Br})		103.2	73.9	85.4	69.1	75.8	69.2	/	/	/	^{13}C DP J-INADEQUATE	Lowman et al. 2011[4]
β-1,6-glucan		103.7	74.2	76.4	70.5	75.2	69.5	/	/	/		
α-1,2-Mannan	a	101.3	79.1	71.1	67.9	74.3	61.9	/	/	/		Latgé *et al*. 1994[5] Chakraborty *et al*. 2021[6]
	b	99.2	79.4	71.8	67.7	73.8	62.2	/	/	/		
α-1,6-Mannan		102.8	70.8	73.7	67.5	71.2	66.8	/	/	/		

Supplementary Table 3. ^{13}C-T_1 and ^{1}H-$T_{1\rho}$ relaxation time constants with single exponential fitting. A single exponential equation was used to fit the T_1 data $I(t) = e^{-t/T_I}$. A single exponential equation was used to fit the $T_{1\rho}$ data: $I(t) = e^{-t/T_{I\rho}}$. Error bars are standard deviations of the fit parameters.

Sample Type	Cross peaks	T_1 (s)	Cross peaks	$T_{1\rho}$ (ms)
Wildtype	B1	1.5±0.08	B1	10.4±0.61
	B3	1.4±0.10	B3	11.2±0.72
	B5	1.3±0.10	B5	11.3±0.79
	B2	1.4±0.08	B2	10.0±0.83
	B4	1.3±0.07	B4	11.5±0.64
	Ch1	1.3±0.07	Ch1	9.2±0.61
	Ch4	1.9±0.26	Ch4	5.2±1.40
	Ch2	1.5±0.37	Ch2	6.1±0.57
	H3	1.1±0.11	H3	6.9±0.47
	H5	1.2±0.15	H5	7.0±0.83
	H6	1.2±0.08	H6	7.0±0.79
	Mn1	0.4±0.03	Mn1	2.3±0.38
	Mn2	1.3±0.14	Mn2	4.8±0.89
Phosphate transport mutant (JKC 2830)	B1	1.4±0.07	B1	12.5±0.99
	B3	1.4±0.03	B3	13.6±1.24
	B5	1.4±0.08	B5	12.4±1.10
	B2	1.6±0.13	B2	11.2±0.94
	B4	1.4±0.08	B4	13.4±1.20
	Ch1	1.5±0.07	Ch1	12.6±0.85
	Ch4	2.5±0.45	Ch4	6.4±1.03
	Ch2	2.2±0.34	Ch2	9.8±1.03
	H3	0.9±0.15	H3	5.9±0.80
	H5	1.5±0.28	H5	8.2±0.80
	H6	1.4±0.23	H6	10.6±0.63
	Mn1	0.6±0.06	Mn1	4.8±0.32
	Mn2	1.2±0.15	Mn2	7.2±0.68

Supplementary Table 4. ^{1}H and ^{13}C chemical shift of mobile carbohydrates of *C. albicans* wildtype cells.

Carbohydrates	forms	C1	C2	C3	C4	C5	C6	Reference
β-1,3-glucan (B)	a	103.31<br>4.51	74.10<br>4.33	85.0<br>4.12	70.30<br>4.21	75.80<br>3.62	61.6<br>3.8, 3.9	Shim *et al.* 2007[1]<br>Fairweather *et al.* 2009[7]<br>Saito *et al.* 1979[8]
	b	---	74.40	84.75<br>4.12	70.37	---	61.79<br>3.80,3.90	Lowman *et al.* 2011[9]
	c	---	74.53<br>4.33	86.46<br>4.27	71.32<br>4.41	---	62.35<br>3.83, 3.90	
	d	---	74.2<br>4.32	85.95<br>4.21	71.38<br>4.42	---	62.19<br>3.8, 3.9	
β-1,3,6-glucan (Br)	a	103.2<br>4.51	75.0<br>3.6	85.27<br>3.75	73.61<br>3.54	---	69.13<br>3.92	
	b			85.74<br>4.6			68<br>4.0	
β-1,6-glucan (H)								
	b	103.54<br>4.52	74.01<br>3.33	76.3<br>3.5	69.9<br>3.47	---	---	
	d	102.63<br>4.46	74.4<br>3.34	76<br>3.5	69.56<br>3.85			
α-1,6-Mannan ($Mn^{1,6}$)		102.75<br>5.15	70.92<br>4.08	---	67.64<br>3.67	70.92<br>4.08	---	Latge *et al.* 1994[10]<br>Chakraborty *et al.* 2021[11]<br>Kuraoka *et al.* 2021[12]
α-1,2-Mannan ($Mn^{1,2}$)	a	101.28<br>5.27	79.10<br>4.10	70.90<br>4.00	67.80<br>3.70	74.00<br>3.70	61.80<br>3.80/3.90	
	b	98.82<br>5.11	79.3<br>4.00	71.19<br>3.88	67.00<br>3.80	---	61.98<br>3.84	Kuraoka *et al.* 2021[12]<br>Kuraoka *et al.* 2018[13]
	c	100.65<br>5.15	78.16<br>4.26	70.04<br>3.91	68.18<br>3.7	73.78<br>3.70	61.40<br>3.76, 3.9	
	d	101.2<br>5.38	79.00<br>4.10	70.80<br>4.10	67.96<br>3.70	73.77<br>3.70	61.76<br>3.76, 3.90	
	e	102.38<br>5.05	78.37<br>3.93	70.62<br>4.08	67.4<br>3.66	73.89<br>3.3	61.80<br>3.75, 3.9	

	f	101.33<br>5.27	79.10<br>4.10	70.9<br>4.0	67.8<br>3.70	74.00<br>3.70	61.80<br>3.80,3.90	
	g	100.66<br>5.34	78.93<br>4.10	70.80<br>4.0	67.75<br>3.7	73.68<br>3.7	61.87<br>3.7, 3.9	
	h	102.57<br>5.03	78.76<br>3.93	70.69<br>4.08	66.3<br>3.90	73.95<br>3.7	61.80<br>3.75, 3.9	
	i	99.5<br>4.85	79.37<br>4.26	---	67.88<br>3.62	72.43<br>3.69	61.51<br>3.78, 3.92	
	j	100.06<br>4.92	---	71.1<br>3.87	67.2<br>3.61	---	61.82<br>3.84<br>3.68	
	k	101.63<br>4.85	77.43<br>4.22	70.88<br>4.08	67.61<br>3.67	73.89<br>3.74	62.0<br>3.76, 3.9	
	l	101.71<br>4.93	77.87<br>4.25	---	68.41<br>3.7	73.67<br>3.63	61.91<br>3.76, 3.9	
	m	94.79<br>5.54	78.77<br>4.19	69.66<br>4.0	67.56<br>3.67	---	61.11<br>3.77, 3.83	
	a	90.00<br>5.90	---	74.30<br>4.30	70.30<br>4.20	--	61.70<br>3.80/ 3.90	Fontaine *et al.* 2011[14]
	b	96.84<br>5.46	---	74.39<br>3.83	70.87<br>4.03	67.2<br>3.7	61.56<br>3.7, 3.83	
Galactose/Glucose or their derivatives (Gl)	c	94.61<br>4.90	71.60<br>3.94	73.65<br>3.66	67.00<br>3.60	---	61.83<br>3.70, 3.80	
	d	94.95<br>5.18	---	71.15<br>3.85	67.34<br>3.66	---	61.7<br>3.70, 3.80	Archbald et al. 1981[15]<br>Fontaine *et al.* 2011[14]
	e	89.26<br>6.04	---	74.32<br>4.33	71.24<br>4.42	---	62.28<br>3.80, 3.90	
	f	88.51<br>5.87	---	74.37<br>4.33	71.16<br>4.40	---	62.46<br>3.83, 3.9	
Glucose (Glc)	a (α)	92.94<br>5.20	72.40<br>3.50	72.40<br>3.50	70.60<br>3.40	---	61.56<br>3.70, 3.80	Archbald *et al.* 1981[15]
	b (β)	96.72<br>4.64	75.0<br>3.25	76.3<br>3.50	70.50<br>3.40	---	61.66<br>3.70, 3.83	

Supplementary Table 5. Experimental parameters used for both wildtype and phosphate transport mutant of *C. albicans*. Mobile region of the cell wall was characterized using scalar-coupling (*J*) based experiments on 800 MHz (18.8 T) spectrometer using 3.2 mm triple resonance MAS probe with the MAS frequency of 15 kHz.

Experiments	MAS	D1	NS	td2	td1	td3	aq2 (ms)	aq1 (ms)	aq3 (ms)	Decoupling/ Water suppression	*J*-evolution (ms)	DIPSI-3 (ms)	Expt. Time (h)
2D hCCH TOCSY (DIPSI-3)	15	2	32	2614 (^{1}H)	512 (^{13}C)	1	39.9	10.2	-	SPINAL-64 (rf 71.429 kHz)<br>WALTZ-16 (rf 17 kHz)<br>MISSISSIPI (total duration) 40 ms (rf 25.994 kHz)	1.78 (τ_1)<br>1.19 (τ_2	25.5	9h42m
3D hCCH TOCSY (DIPSI-3)	15	2	8	2614 (^{1}H)	128 (^{13}C)	128 (^{13}C)	39.9	2.56	2.56				77h53m

Supplementary Table 6. Linewidth (FWHM) of resolved carbohydrate resonances in 2D ^{13}C-^{13}C CORD spectra. Representative linewidth (FWHM) measurements of α-1,2-mannose and β-1,3-glucan cross peaks from 2D ^{13}C-^{13}C CORD spectra of wildtype and phosphate transport mutant *C. albicans* cell wall from two independent biological replicates under control and H_2O_2 oxidative stress conditions.

Carbohydrates	Cross-peak (ppm)	Batch	WT control LW (Hz/ppm)	WT H_2O_2 LW (Hz/ppm)	Δ LW WT (Hz/ppm)	Mutant control LW (Hz/ppm)	Mutant H_2O_2 LW (Hz/ppm)	Δ LW Mutant (Hz/ppm)
α-1,2-Mannose	101.5	1	270.1/1.34	554.6/2.75	284.5/1.41	272.7/1.35	411.3/2.04	138.5/0.69
α-1,2-Mannose	101.5	2	242.9/1.20	435.6/2.16	192.6/0.96	248.5/1.23	332.9/1.65	84.4/0.42
α-1,2-Mannose	79.8	1	267.4/1.33	298.6/1.48	31.2/0.15	279.2/1.38	306.1/1.52	26.9/0.14
α-1,2-Mannose	79.8	2	287.2/1.42	335.1/1.66	47.9/0.24	241.9/1.20	313.1/1.55	71.2/0.35
α-1,2-Mannose	73.6	1	488.5/2.43	633.2/3.15	144.7/0.72	504.3/2.50	618.4/3.07	114.0/0.57
α-1,2-Mannose	73.6	2	403.0/2.00	783.0/4.88	379.9/2.88	408.3/2.02	735.8/3.65	327.5/1.63
β-1,3-glucan	103.8	1	155.3/0.77	244.5/1.21	89.2/0.44	213.2/1.06	220.8/1.09	7.5/0.03
β-1,3-glucan	103.8	2	207.9/1.03	229.5/1.14	21.5/0.11	207.1/1.02	221.9/1.10	14.7/0.08
β-1,3-glucan	74.6	1	239.0/1.18	277.4/1.37	38.3/0.19	262.2/1.30	280.0/1.39	17.7/0.09
β-1,3-glucan	74.6	2	246.5/1.22	250.8/1.24	4.3/0.02	253.8/1.26	265.6/1.31	11.7/0.05
β-1,3-glucan	86.8	1	224.5/1.11	327.7/1.63	103.2/0.52	252.8/1.25	301.3/1.49	48.5/0.24
β-1,3-glucan	86.8	2	242.3/1.20	274.2/1.36	31.8/0.16	243.9/1.21	261.8/1.30	17.9/0.09
β-1,3-glucan	68.5	1	182.2/0.90	233.6/1.16	51.4/0.26	229.3/1.14	234.5/1.16	5.2/0.02
β-1,3-glucan	68.5	2	222.9/1.10	243.1/1.20	20.1/0.10	233.1/1.15	238.9/1.18	5.7/0.03
β-1,3-glucan	77.7	1	222.2/1.10	254.3/1.26	32.1/0.16	232.0/1.15	254.1/1.26	22.1/0.11
β-1,3-glucan	77.7	2	226.7/1.12	241.1/1.19	14.3/0.07	231.2/1.14	236.2/1.17	5.0/0.03
β-1,3-glucan	61.6	1	213.0/1.05	281.1/1.39	68.0/0.34	210.5/1.04	265.4/1.31	54.8/0.27
β-1,3-glucan	61.6	2	219.7/1.09	231.4/1.15	11.7/0.06	218.5/1.08	234.1/1.16	15.5/0.08

Supplementary References

1 Shim, J. H. *et al.* Antitumor Effect of Soluble b-1,3-Glucan from Agrobacterium sp. R259 KCTC 1019. *J. Microbiol. Biotechnol.* **17**, 1513-1520 (2007).

2 Fairweather, J. K., Him, J. L. K., Heux, L., Driguez, H. & Bulone, V. Structural characterization by ^{13}C-NMR spectroscopy of products synthesized in vitro by polysaccharide synthases using ^{13}C-enriched glycosyl donors: application to a UDP-glucose:(1→ 3)-b-d-glucan synthase from blackberry (Rubus fruticosus). *Glycobiology* **14**, 775-781 (2004).

3 Saitô, H., Ohki, T. & Sasaki, T. A 13C-nuclear magnetic resonance study of polysaccharide gels. Molecular architecture in the gels consisting of fungal, branched (1→ 3)-β-D-glucans (lentinan and schizophyllan) as manifested by conformational changes induced by sodium hydroxide. *Carbohydrate Research* **74**, 227-240 (1979).

4 Lowman, D. W. *et al.* New insights into the structure of (1→ 3, 1→ 6)-β-D-glucan side chains in the Candida glabrata cell wall. *PloS one* **6**, e27614 (2011).

5 Latge, J.-P. *et al.* Chemical and immunological characterization of the extracellular galactomannan of Aspergillus fumigatus. *Infection and immunity* **62**, 5424-5433 (1994).

6 Chakraborty, A. *et al.* A molecular vision of fungal cell wall organization by functional genomics and solid-state NMR. *Nat. Comm.* **12**, 6346 (2021).

7 Fairweather, J. K., Him, J. L. K., Heux, L., Driguez, H. & Bulone, V. Structural characterization by ^{13}C-NMR spectroscopy of products synthesized in vitro by polysaccharide synthases using ^{13}C-enriched glycosyl donors: application to a UDP-glucose:(1→ 3)-b-d-glucan synthase from blackberry (Rubus fruticosus). *Glycobiology* **14**, 775-781 (2004).

8 Saitô, H., Ohki, T. & Sasaki, T. A 13C-nuclear magnetic resonance study of polysaccharide gels. Molecular architecture in the gels consisting of fungal, branched (1→ 3)-b-D-glucans (lentinan and schizophyllan) as manifested by conformational changes induced by sodium hydroxide. *Carbohydr. Res.* **74**, 227-240 (1979).

9 Lowman, D. W. *et al.* New Insights into the Structure of (1→3,1→6)-β-D-Glucan Side Chains in the Candida glabrata Cell Wall. *PLoS One* **6**, e27614 (2011).

10 Latge, J. P. *et al.* Chemical and immunological characterization of the extracellular galactomannan of Aspergillus fumigatus. *Infect. Immun.* **62**, 5424-5433 (1994).

11 Chakraborty, A. *et al.* A molecular vision of fungal cell wall organization by functional genomics and solid-state NMR. *Nat. Commun.* **12**, 6346 (2021).

12 Kuraoka, T., Yamada, T., Takatsutsumi, Y., Ogawa, Y. & Kobayashi, H. Anomeric Proton and Carbon (H1-C1) NMR Chemical Shifts of Antigenic Mannans Obtained from Pathogenic Yeast Candida tropicalis. *Adv. Microbiol.* **11**, 296-301 (2021).

13 Kuraoka, T., Ishiyama, A., Oyamada, H., Ogawa, Y. & Kobayashi, H. Presence of O-glycosidically linked oligosaccharides in the cell wall mannan of Candida krusei purified with Benanomicin A. *FEBS Open Bio.* **9**, 129-136 (2018).

14 Fontaine, T. *et al.* Galactosaminogalactan, a New Immunosuppressive Polysaccharide of Aspergillus fumigatus. *PLoS Pathog.* **7**, e1002372 (2011).

15 Archbald, P. J., Fenn, M. D. & Roy, A. B. 13C-N.M.R. studies of D-glucose and D-galactose monosulphates. *Carbohydr. Res.* **93**, 177-190 (1981).
