# Supplementary material for: Oxidative stress-responsive cell wall remodeling depends on phosphate in Candida albicans
Source: Nat Commun. 2026 Sep 10;17:10676. doi: 10.1038/s41467-026-77594-x (PMC13654929; doi:10.1038/s41467-026-77594-x)
Supplement: Supplementary file 2 [file 41467_2026_77594_MOESM2_ESM.pdf]

nature portfolio

Corresponding author(s): Tuo Wang

Last updated by author(s): 07/30/2026

Reporting Summary

Nature Portfolio wishes to improve the reproducibility of the work that we publish. This form provides structure for consistency and transparency in reporting. For further information on Nature Portfolio policies, see our Editorial Policies and the Editorial Policy Checklist.

Statistics

For all statistical analyses, confirm that the following items are present in the figure legend, table legend, main text, or Methods section.

n/a	Confirmed	
☐	☒	The exact sample size (*n*) for each experimental group/condition, given as a discrete number and unit of measurement
☐	☒	A statement on whether measurements were taken from distinct samples or whether the same sample was measured repeatedly
☐	☒	The statistical test(s) used AND whether they are one- or two-sided<br>*Only common tests should be described solely by name; describe more complex techniques in the Methods section.*
☒	☐	A description of all covariates tested
☒	☐	A description of any assumptions or corrections, such as tests of normality and adjustment for multiple comparisons
☐	☒	A full description of the statistical parameters including central tendency (e.g. means) or other basic estimates (e.g. regression coefficient) AND variation (e.g. standard deviation) or associated estimates of uncertainty (e.g. confidence intervals)
☐	☒	For null hypothesis testing, the test statistic (e.g. *F*, *t*, *r*) with confidence intervals, effect sizes, degrees of freedom and *P* value noted<br>*Give P values as exact values whenever suitable.*
☒	☐	For Bayesian analysis, information on the choice of priors and Markov chain Monte Carlo settings
☒	☐	For hierarchical and complex designs, identification of the appropriate level for tests and full reporting of outcomes
☒	☐	Estimates of effect sizes (e.g. Cohen's *d*, Pearson's *r*), indicating how they were calculated

Our web collection on statistics for biologists contains articles on many of the points above.

Software and code

Policy information about availability of computer code

Data collection	The data collected on Topspin version 3.5 on 800 MHz solid-state NMR spectrometer.
Data analysis	The graphs were generated through OriginPro 9.0<br>The NMR spectra were analyzed and processed using TopSpin 4.3.0<br>The figures and illustrations are drawn in Adobe Illustrator v27.2.0

For manuscripts utilizing custom algorithms or software that are central to the research but not yet described in published literature, software must be made available to editors and reviewers. We strongly encourage code deposition in a community repository (e.g. GitHub). See the Nature Portfolio guidelines for submitting code & software for further information.

Data

Policy information about availability of data

All manuscripts must include a data availability statement. This statement should provide the following information, where applicable:

- Accession codes, unique identifiers, or web links for publicly available datasets
- A description of any restrictions on data availability
- For clinical datasets or third party data, please ensure that the statement adheres to our policy

All relevant data that support the findings of this study are provided in the article and Supplementary Information. All the original ssNMR data files have been deposited in the Zenodo repository and can be freely accessed (DOI: 10.5281/zenodo.21690324; accessible at https://zenodo.org/records/21690324). MD input data and analysis are available for download, and selected outputs are also available from Zenodo (DOI: 10.5281/zenodo.19411396; accessible at https://

doi.org/10.5281/zenodo.19411396). Source Data are provided as a source data file.

Research involving human participants, their data, or biological material

Policy information about studies with human participants or human data. See also policy information about sex, gender (identity/presentation), and sexual orientation and race, ethnicity and racism.

Reporting on sex and gender	sex and gender analysis is not applicable to this fundamental study of fungal cell walls.
Reporting on race, ethnicity, or other socially relevant groupings	Not applicable to fundamental structural analysis of fungal cell walls.
Population characteristics	Not applicable to fundamental structural analysis of fungal cell walls.
Recruitment	Not applicable to fundamental structural analysis of fungal cell walls.
Ethics oversight	Not applicable to fundamental structural analysis of fungal cell walls.

Note that full information on the approval of the study protocol must also be provided in the manuscript.

Field-specific reporting

Please select the one below that is the best fit for your research. If you are not sure, read the appropriate sections before making your selection.

[x] Life sciences [ ] Behavioural & social sciences [ ] Ecological, evolutionary & environmental sciences

For a reference copy of the document with all sections, see nature.com/documents/nr-reporting-summary-flat.pdf

Life sciences study design

All studies must disclose on these points even when the disclosure is negative.

Sample size	ssNMR rotor was used to contain the samples during measurements. Approximately 30-40 mg of fungal materials (native hydrated mass) were packed in 3.2 mm rotors for all high-resolution measurements on 3.2 mm probes. The mass was determined by weighing the material before and after packing into a NMR rotor. The NMR spectra report the average feature of all cells in each sample. A large number of scans were collected for averaging and for each NMR spectrum, resulting in reproducibility of each spectrum. The exact number of scans are summarized in Supplementary Tables 1 and 5.
Data exclusions	There were no data exclusions.
Replication	Each NMR experiment were average by number of scans. All the parameters for NMR experiments are provided in Supplementary Tables 1 and 5. 1D experiments were frequently measured to monitor the sample status before and after each 2D experiment. 2D experiments were replicated as individual blocks. All attempts of replication were successful. One batch of replicate was used for confirming NMR linewidth changes induced by H_2O_2.
Randomization	Different fungal samples were measured by multiple group members independently in a randomized manner, without special allocation.
Blinding	Not Applicable to NMR studies of the fundamental structure of carbohydrate molecules. As the samples studied are natural mixtures of molecules, there is no differentiation and there is no need for randomized controlled trial. The outcome parameters are not subjective and has no bias.

Reporting for specific materials, systems and methods

We require information from authors about some types of materials, experimental systems and methods used in many studies. Here, indicate whether each material, system or method listed is relevant to your study. If you are not sure if a list item applies to your research, read the appropriate section before selecting a response.

Materials & experimental systems

n/a	Involved in the study	
[x]	[ ]	Antibodies
[x]	[ ]	Eukaryotic cell lines
[x]	[ ]	Palaeontology and archaeology
[x]	[ ]	Animals and other organisms
[x]	[ ]	Clinical data
[x]	[ ]	Dual use research of concern
[x]	[ ]	Plants

Methods

n/a	Involved in the study	
[x]	[ ]	ChIP-seq
[x]	[ ]	Flow cytometry
[x]	[ ]	MRI-based neuroimaging

Plants

Seed stocks	This study does not include any plant material
Novel plant genotypes	No plant included
Authentication	Not relevant to this study; no plant material involved
