# Supplementary material for: Oxidative stress-responsive cell wall remodeling depends on phosphate in Candida albicans
Source: Nat Commun. 2026 Sep 10;17:10676. doi: 10.1038/s41467-026-77594-x (PMC13654929; doi:10.1038/s41467-026-77594-x)
Supplement: Supplementary file 3 [file 41467_2026_77594_MOESM3_ESM.pdf]

nature portfolio

Peer Review File

Oxidative Stress-Responsive Cell Wall Remodeling Depends on Phosphate in Candida albicans

Corresponding Author: Professor Tuo Wang

This file contains all reviewer reports in order by version, followed by all author rebuttals in order by version.

Version 0:

Reviewer comments:

Reviewer #1

(Remarks to the Author)
In this manuscript, Jacob, Köhler, and Wang present a molecular study on the impact of phosphate-deprivation and oxidative stress on the cell wall of the fungus Candida albicans.

Candida albicans is a high-priority fungal pathogen that, when turning from commensal to invasive, experiences the two-fold challenge of phosphate-deprivation and oxidative stress. In particular, the effect of the latter – oxidative stress – on the fungal cell envelope has been understudied at conditions considered physiologically relevant. In this manuscript, the authors use comprehensive high-resolution solid-state NMR studies in intact Candida albicans cells to explore the effect the combined effect of phosphate-deprivation and oxidative stress, and the use phosphate transport mutants to disentangle these effects. These studies build upon a truly remarkable series of the Wang group to dissect the molecular architecture of fungal cells under various stress conditions.

Overall, this a very interesting study. The authors convincingly demonstrate that phosphate deprivation – in the absence of oxidative stress - rigidifies and condenses the fungal cell envelope. Using a very nice set of 1H-detected ssNMR data, they also show a homogenization of the cell envelope under these conditions as compared to phosphate-replete conditions.

Subsequently, the authors explore the impact of oxidative stress. To this end, they exposed cells to 0.8 M H2O2. Under these conditions, the authors observe extended damage in all fractions (highly mobile, mobile, rigid core) of the cell envelope of wild-type channels, but this damage is limited to the highly mobile + mobile fractions in the phosphate transport mutant, while the rigid core shows only reduced ROS damage in. Thus, this leads to the conclusion that under combined phosphate-deprivation and oxidative stress, the rigid core of Candida albicans cell envelopes is less susceptible to ROS damage – and this is the key finding of this manuscript.

At the same time, I consider the later (and most critical) finding not well documented. I agree with the authors that spectrum 3C shows a substantial impact of H2O2 on the rigid fraction, which is not observed in the mutant. However, the 1D spectrum of the mutant is already so broadened that I wonder if a H2O2 impact can be visualized in 1D. And the differences in Figure 3E are quite modest and – in either case – difficult to gauge from a 2D spectrum. Cross-sections would be much more meaningful, and the authors should also make these 2D data accessible for the reviewing process.

Minor comment:
1. While I'm knowledgeable in biological solid-state NMR, I do not quite understand the data shown in Figure 2B,C. For me at least, it would help if the authors used conventional NMR-labels of the dimensions and denote at which frequency of the 3rd dimensions planes are extracted (if this how to look at these data?).
2. Figure 3C: Why is the 13C spectrum of the mutant much broader than for the WT if such conditions (see Figure 2) are supposed to reduce polymorphism?

Reviewer #2

(Remarks to the Author)

The manuscript titled "Oxidative Stress-Responsive Cell Wall Remodeling Depends on Phosphate in Candida albicans" by Jacob et al., is an important work in the field of Candidiasis and finding potential therapeutics. It explores the changes in the fungal cell wall in response to oxidative stress encountered in the host system and its dependence on phosphate. A deeper understanding of this mechanism could prove be an avenue to develop antifungal drugs, therefore, the subject is quite relevant.
The work established that the cell wall acts as an active participant in oxidative protection and show that nutrient availability acts as a structural regulator of ROS resistance. Severe restriction of inorganic phosphate uptake extensively remodels the rigid wall core. Using NMR, the authors showed differences between rigid core organization of wild-type and phosphate transport deficient mutant. Upon peroxide exposure, the wild-type rigid core was found to be better accomodated to intercept ROS than the phosphate deficient mutant. Molecular dynamics showed that H2O2 is not intrinsically selective for any particular polymer.

With my expertise in molecular dynamics simulations and being invited for the same, I have technically reviewed only that section. Each simulation was carried out 5 times for 1000 nanoseconds for each complex. This is sufficient time to obtain robust sampling of events. The details provided in the methodology are enough for reproducibility. It is just a suggestion for future work, it would be interesting to simulate these systems with the cell wall components packed in a way to be mimicking a small patch of the wild-type cell wall and phosphate-deficient type. It could be interesting to observe the interaction with water and peroxide molecules in a packed system.

Reviewer #3

(Remarks to the Author)
The manuscript describes solid state NMR analysis of the fungal cell wall of Candida albicans parental strain vs phosphate transport mutant, lacking all 4 phosphate transporters. It then analysis alterations in cell wall structure when cells are exposed to hydrogen peroxide in combination with the phosphate deprivation. This is not a combination of stresses that I would have prioritised to study and I suspect the rationale behind the choice of stresses to study was influenced by the availability of the phosphate transport mutant. The manuscript goes to lengths to describe physiological levels of phosphate that C. albicans will be exposed to, in commensal versus invasive states, and also oxidative stresses that the fungus will encounter in the human host, but the assays are performed in completely synthetic medium, which is not physiologically relevant. The authors chose to use a very mild oxidative stress with 0.8 mM hydrogen peroxide.
Keep points that were not described in the manuscript, what is the level of stress imposed with 0.8 mM hydrogen peroxide, the authors state it was sub-lethal but did it have any effect on cell growth? Were the morphology of cells examined under the different culture conditions, did the phosphate deprivation and/or hydrogen peroxide treatment alter cell morphology which could also impact on cell wall changes. 5mM hydrogen peroxide is known to induce formation of hyper-polarized buds.
I'm surprised there was no mention of the phosphomannan (or phosphopeptidomannan) component of the cell wall? This would be important as the beta-mannosyltransferases (BMT3, BMT4) were upregulated in phosphate deplete conditions (stated in introduction). beta-mannosyltransferases add mannose residues to the phosphomannan chain and if phosphate is lacking perhaps beta-mannosylation is happening on different glycan chains?
Another component you might expect to have been altered is the mannan attached to GPI-modified cell wall proteins, lack of phosphate may impinge on post-translational modification by addition of GPI anchors and eventually alter the outer mannan coat. Alterations in the outer fibrillar mannan layer could be detected by transmission electron microscopy, combining microscopy with the solid NMR data would really solidify the interesting changes detected in the cell wall polysaccharides in this study.
For the N-glycans that constitute mannan the conventional model is an alpha-1,6-linked backbone with alpha-1,2- or -1,3-linked sidechains. Your description (line 94, Line 206/207) would contradict this you state there is an α1,2-linked backbone. This also contradicts your previous paper (https://doi.org/10.1038/s41467-025-61678-1).
Figure 5, is quite hard to follow. Why is the chitin not cross-linked to beta-1,3-glucan in the figure?
What is the evidence that chitin synthesis in the mutant results in deposition in the outer layer of the wall? Do you have a model of how chitin which is synthesize by membrane proteins, normally localised close to the plasma membrane would get localised to the outer wall?
Chitin is usually cross-linked to beta 1,3 glucan and in past cell wall stress studies has reported changes in the number of cross-links, this is reinforced by upregulation of carbohydrate active enzymes under those conditions. And under cell wall stress it has been suggested there are more bonds between chitin and beta 1,6 glucan, any evidence for this?

Version 1:

Reviewer comments:

Reviewer #1

(Remarks to the Author)
I generally appreciate the authors comprehensive rebuttal - but I would feel that some of the conclusions made by the authors are not yet solid enough to judge without some form of statistics.

In particular, I appreciate that the authors provided cross-sections of the 2D CC spectra (new Supplementary Figure 11). I

have also analyzed the raw data (thank you also for providing these data!). Notwithstanding, the - apparent – LW differences between WT and mutant are sometimes less than 20 Hz. This does not seem convincing without some sort of statistics – NMR spectroscopist have observed such LW variations between different sample preparations.

I hence like to ask the authors the following: Please list, in a table format, a LW comparison for all resolved cross-peaks of α-1,2-mannose and β-1,3-glucan, and lease confirm these LW differences with a 2nd set of biologically independent samples. If this article got accepted, please also make the raw NMR data available.

Reviewer #3

(Remarks to the Author)
I would like to thank the reviewers for modifying the manuscript according to reviewers suggestions. I believe the new data/figures such as the images of cell morphology and the TEM to measure cell wall thickness have strengthened the manuscript. I am satisfied that all points have been adequately addressed.

Version 2:

Reviewer comments:

Reviewer #1

(Remarks to the Author)
I thank the authors for their rigorous data analysis and for providing the raw data. For me this manuscript could be advanced to publication stage. Congratulations to this very meaningful work.

Markus Weingarth.

Open Access This Peer Review File is licensed under a Creative Commons Attribution 4.0 International License, which permits use, sharing, adaptation, distribution and reproduction in any medium or format, as long as you give appropriate credit to the original author(s) and the source, provide a link to the Creative Commons license, and indicate if changes were made.
In cases where reviewers are anonymous, credit should be given to 'Anonymous Referee' and the source.
The images or other third party material in this Peer Review File are included in the article's Creative Commons license, unless indicated otherwise in a credit line to the material. If material is not included in the article's Creative Commons license and your intended use is not permitted by statutory regulation or exceeds the permitted use, you will need to obtain permission directly from the copyright holder.
To view a copy of this license, visit https://creativecommons.org/licenses/by/4.0/

Responses to Reviewers' Comments

Reviewer 1

In this manuscript, Jacob, Köhler, and Wang present a molecular study on the impact of phosphate-deprivation and oxidative stress on the cell wall of the fungus Candida albicans. Candida albicans is a high-priority fungal pathogen that, when turning from commensal to invasive, experiences the two-fold challenge of phosphate-deprivation and oxidative stress. In particular, the effect of the latter – oxidative stress – on the fungal cell envelope has been understudied at conditions considered physiologically relevant. In this manuscript, the authors use comprehensive high-resolution solid-state NMR studies in intact Candida albicans cells to explore the effect the combined effect of phosphate-deprivation and oxidative stress, and the use phosphate transport mutants to disentangle these effects. These studies build upon a truly remarkable series of the Wang group to dissect the molecular architecture of fungal cells under various stress conditions. Overall, this a very interesting study. The authors convincingly demonstrate that phosphate deprivation – in the absence of oxidative stress - rigidifies and condenses the fungal cell envelope. Using a very nice set of 1H-detected ssNMR data, they also show a homogenization of the cell envelope under these conditions as compared to phosphate-replete conditions. Subsequently, the authors explore the impact of oxidative stress. To this end, they exposed cells to 0.8 M H2O2. Under these conditions, the authors observe extended damage in all fractions (highly mobile, mobile, rigid core) of the cell envelope of wild-type channels, but this damage is limited to the highly mobile + mobile fractions in the phosphate transport mutant, while the rigid core shows only reduced ROS damage in. Thus, this leads to the conclusion that under combined phosphate-deprivation and oxidative stress, the rigid core of Candida albicans cell envelopes is less susceptible to ROS damage and this is the key finding of this manuscript.

Response: We are very grateful to the reviewer for their positive and supportive feedback.

At the same time, I consider the later (and most critical) finding not well documented. I agree with the authors that spectrum 13C shows a substantial impact of H2O2 on the rigid fraction, which is not observed in the mutant. However, the 1D spectrum of the mutant is already so broadened that I wonder if a H2O2 impact can be visualized in 1D. And the differences in Figure 3E are quite modest and – in either case – difficult to gauge from a 2D spectrum. Cross-sections would be much more meaningful, and the authors should also make these 2D data accessible for the reviewing process.

Response: Following the guide, we have now added analysis of 1D cross-sections extracted from the 2D ^{13}C-^{13}C CORD spectra (**Supplementary Fig. 11**) to improve the interpretability of line-broadening. These cross-sections enable a more direct comparison of broadness (PRE effect) between control and H_2O_2 treated samples, particularly in regions corresponding to α-1,2-mannose and β-1,3-glucan. Full width at half maximum (FWHM) values are measured to quantitatively evaluate peroxide induced line broadening in the cell wall polysaccharides of the wildtype and phosphate transport mutant. The substantially larger linewidth increase observed in the H_2O_2 treated wildtype relative to the mutant indicates stronger paramagnetic perturbation and a greater structural response of the wildtype cell wall. The cross-sectional analysis confirms that, despite the intrinsically broader lines in the mutant control spectra, H_2O_2-induced perturbations remain minimal relative to the wildtype, where pronounced paramagnetic broadening is clearly evident. Furthermore, the inclusion of these cross-sections

improves the visualization of subtle spectral changes that are otherwise difficult to discern in the 2D spectra. All corresponding 2D spectra have now been made available in Zenodo with DOI provided in the Data Availability section.

The added sentences in the maintext read: "These peroxide-induced line-broadening effects were further confirmed by analysis of 1D cross-sections extracted from the 2D CORD spectra (**Supplementary Fig. 11**), enabling direct assessment of resonance linewidths and PRE-associated signal attenuation otherwise masked by spectral congestion in rigid polysaccharide resonances."

Minor comment:
1. While I'm knowledgeable in biological solid-state NMR, I do not quite understand the data shown in Figure 2B,C. For me at least, it would help if the authors used conventional NMR-labels of the dimensions and denote at which frequency of the 3rd dimensions planes are extracted (if this how to look at these data?).

Response: We have now updated **Figs. 2b, c** and the corresponding captions to include information regarding the dimensions and extracted planes/strips. Specifically, an inset has been added to Fig. 2b to illustrate the three-dimensional experiment and to show a representative extraction of a ^{13}C-^{1}H (δ_1-δ_3) plane to help understand the concept. We have also added the labels δ_1(^{13}C), δ_2(^{13}C) and δ_3(^{1}H) to indicate all dimensions of the 2D planes. In addition, the carbon and proton sites corresponding to each extracted 2D plane are now labeled at the top of each panel. Furthermore, more detailed descriptions of the carbon numbering, extraction chemical shifts, and dimensional assignments have been included in the corresponding figure captions.

2. Figure 3C: Why is the 13C spectrum of the mutant much broader than for the WT if such conditions (see Figure 2) are supposed to reduce polymorphism?

Response: The apparent incongruity arises because the two experiments probe distinct molecular domains of the cell wall. The ^{1}H-detected spectra shown in **Fig. 2** were acquired specifically to evaluate how phosphate starvation remodels the mobile polysaccharide matrix. In these spectra, the phosphate transport mutant exhibits simplified and more homogeneous mobile-domain structures, resulting in reduced polymorphism in the ^{1}H-detected signals. In contrast, the ^{13}C CP spectra shown in **Fig. 3c** probe the rigid core of the cell wall, with black and orange spectra representing the control and H_2O_2-treated samples, respectively, for both the wild type and mutant strains. Because the ^{13}C CP experiment selectively detects rigid polysaccharides, it is particularly sensitive to heterogeneity arising from altered packing and crosslinking within the rigid matrix. Accordingly, even under control conditions, the phosphate transport mutant displays broader ^{13}C lines than the wildtype, reflecting increased conformational heterogeneity in the rigid core, whereas the rigid fraction of the wildtype remains comparatively uniform.

We have now added a clarification in the maintext which reads: "Although the phosphate transport mutant exhibits reduced polymorphism and greater structural homogeneity in the mobile polysaccharide matrix, as shown by hCCH TOCSY (DIPSI-3) spectra in **Fig. 2**, the broader ^{13}C CP

signals reflect increased conformational heterogeneity within the distinct rigid cell-wall core probed in **Fig. 3c**."

Reviewer 2

The manuscript titled "Oxidative Stress-Responsive Cell Wall Remodeling Depends on Phosphate in Candida albicans" by Jacob et al., is an important work in the field of Candidiasis and finding potential therapeutics. It explores the changes in the fungal cell wall in response to oxidative stress encountered in the host system and its dependence on phosphate. A deeper understanding of this mechanism could prove be an avenue to develop antifungal drugs, therefore, the subject is quite relevant.

The work established that the cell wall acts as an active participant in oxidative protection and show that nutrient availability acts as a structural regulator of ROS resistance. Severe restriction of inorganic phosphate uptake extensively remodels the rigid wall core. Using NMR, the authors showed differences between rigid core organization of wild-type and phosphate transport deficient mutant. Upon peroxide exposure, the wild-type rigid core was found to be better accommodated to intercept ROS than the phosphate deficient mutant. Molecular dynamics showed that H2O2 is not intrinsically selective for any particular polymer.

Response: We deeply appreciate the encouraging comments from the reviewer regarding the importance of the work.

With my expertise in molecular dynamics simulations and being invited for the same, I have technically reviewed only that section. Each simulation was carried out 5 times for 1000 nanoseconds for each complex. This is sufficient time to obtain robust sampling of events. The details provided in the methodology are enough for reproducibility.

Response: We sincerely appreciate the reviewer's positive assessment of the molecular dynamics simulations. To further support the robustness of the simulations, we have added a new panel to **Supplementary Fig. 15** showing the peroxide-glycan contact time series for all four simulated systems and their independent replicates. The figure demonstrates that the contacts are transient and dynamic, with minimal variation between replicates, supporting the interaction analysis presented in **Fig. 4**. We have also deposited the MD input file into Zenodo for publica access.

It is just a suggestion for future work, it would be interesting to simulate these systems with the cell wall components packed in a way to be mimicking a small patch of the wild-type cell wall and phosphate-deficient type. It could be interesting to observe the interaction with water and peroxide molecules in a packed system.

Response: We thank the reviewer for this insightful suggestion for future work. This is indeed the way to go. We have now added a sentence in the maintext to mention such future possibilities, which reads "Future simulations incorporating packed polysaccharide assemblies that mimic native wildtype and phosphate-deficient cell-wall architectures may further clarify how mesoscale organization influences water and peroxide accessibility."

Reviewer 3

The manuscript describes solid state NMR analysis of the fungal cell wall of Candida albicans parental strain vs phosphate transport mutant, lacking all 4 phosphate transporters. It then analysis alterations in cell wall structure when cells are exposed to hydrogen peroxide in combination with the phosphate deprivation. This is not a combination of stresses that I would have prioritised to study and I suspect the rationale behind the choice of stresses to study was influenced by the availability of the phosphate transport mutant. The manuscript goes to lengths to describe physiological levels of phosphate that C. albicans will be exposed to, in commensal versus invasive states, and also oxidative stresses that the fungus will encounter in the human host, but the assays are performed in completely synthetic medium, which is not physiologically relevant.

Response: We really appreciate the thoughtful comments. We performed the solid-state NMR studies in defined synthetic medium to maximize experimental reproducibility, since the composition of complex media such as YPD can vary substantially between batches and ingredient sources. Unfortunately, growth of *Candida* cells *in vitro* cannot fully recapitulate host conditions to be physiologically relevant. Neither can specific stressors be recreated *in vitro* in the same way as the fungus encounters them in the host. The *in vivo* situation, even only with respect to the phagocyte encounter, involves a large number of simultaneous stressors like antimicrobial peptides, various proteases, hypochlorous acid, reactive nitrogen species, superoxide anion that is immediately dismutated to H_2O_2, elevated cation concentrations, etc. (e.g. as reported in Lehman H. K. *et al*., 2020; Winterbourn C. C. *et al*., 2006; Kobayashi S. D. *et al*., 2022). The fungus confronts these stressors from a situation of nutrient deprivation, that pertains to glucose, iron, and, as we chose to highlight here, phosphate. Many studies isolate the fungus' response to one, at most two of these stressors, and disregard the effect of nutritional deprivation. We therefore believe that focusing on the interplay between phosphate deprivation and oxidative stress provides new mechanistic insight into how nutrient availability shapes the structural resilience of the fungal cell wall during host-associated stress.

We have now added a sentence to the Discussion to clarify this point: "Although experiments performed in defined synthetic media cannot fully recapitulate the complexity of host-associated stresses, this controlled reductionist approach enables mechanistic isolation of phosphate-dependent oxidative-stress responses."

The authors chose to use a very mild oxidative stress with 0.8 mM hydrogen peroxide. Keep points that were not described in the manuscript, what is the level of stress imposed with 0.8 mM hydrogen peroxide, the authors state it was sub-lethal but did it have any effect on cell growth?

Response: The H_2O_2 concentration to which we exposed the cells, 0.8 mM, was chosen in pilot experiments establishing a concentration at which growth was slightly impaired, indicating a biological impact. We have now included the growth curves of wildtype and phosphate transporter mutant without and with 0.8 mM H_2O_2 in the newly added **Supplementary Fig. 6b**. We intended to examine cell wall remodeling responses of the living fungus to oxidative stress, and therefore deliberately refrained from inflicting global cellular damage with a high H_2O_2 concentration.

With respect to physiological relevance, assays of steady-state reactive oxygen species in neutrophil phagosomes have found that "Superoxide stabilizes at approximately 25 μM and hydrogen peroxide in the low micromolar range," (Winterbourn C. C. *et al*., 2006) but this of course occurs in the context of the other microbicidal factors listed above and therefore cannot be directly extrapolated to Candida cultures grown in synthetic medium with 2% glucose. Hence, we believe the H_2O_2 concentration we chose forces fungal cells to respond, as evinced by their slightly slower growth, without causing catastrophic damage that would preclude cell wall remodeling.

Were the morphology of cells examined under the different culture conditions, did the phosphate deprivation and/or hydrogen peroxide treatment alter cell morphology which could also impact on cell wall changes. 5mM hydrogen peroxide is known to induce formation of hyper-polarized buds.

Response: We did examine the cell morphologies of wildtype and phosphate transporter mutant cells under the experimental conditions, and found no significant alterations. We have now added new **Supplementary Figs. 1a** and **6a**, and stated this point in the maintext:

We added two new sentences which read: "During phosphate starvation, wildtype and mutant cells displayed no substantial differences in overall morphology (**Supplementary Fig. 1**)" and "We ruled out cell morphologic changes as the causes of the observed cell wall alterations at 5 mM H2O2, hyperpolarized bud growth has been observed but no substantial morphological changes were observed in either wildtype or phosphate transport mutant under our conditions (**Supplementary Fig. 6**)."

We also modified one sentence, which now reads: "To assess the impact of oxidative stress on *C. albicans* cell wall architecture, both wildtype and phosphate transport deficient mutant cells were treated with 0.8 mM H_2O_2, a concentration selected to permit sustained growth of treated cells, as determined in pilot experiments that showed minor growth defects of cultures exposed to this concentration (**Supplementary Fig. 6**)."

I'm surprised there was no mention of the phosphomannan (or phosphopeptidomannan) component of the cell wall? This would be important as the beta-mannosyltransferases (BMT3, BMT4) were upregulated in phosphate deplete conditions (stated in introduction). beta-mannosyltransferases add mannose residues to the phosphomannan chain and if phosphate is lacking perhaps beta-mannosylation is happening on different glycan chains? Another component you might expect to have been altered is the mannan attached to GPI-modified cell wall proteins, lack of phosphate may impinge on post-translational modification by addition of GPI anchors and eventually alter the outer mannan coat. Alterations in the outer fibrillar mannan layer could be detected by transmission electron microscopy, combining microscopy with the solid NMR data would really solidify the interesting changes detected in the cell wall polysaccharides in this study.

Response: We fully agree, and we thank the reviewer for this insightful comment regarding the phosphomannan-rich outer layer of the *C. albicans* cell wall and its relationship to phosphate transport deficiency. To address this point, we added new TEM data and Alcian blue staining to combine with solid-state NMR analyses to obtain a more comprehensive understanding of the remodeling of the mannan-rich outer wall layer under impaired phosphate uptake.

TEM revealed a pronounced reduction in cell wall thickness in the phosphate transport mutant, indicating that phosphate uptake is required to maintain normal cell wall structure and integrity (new **Fig. 1a, b**). Alcian blue staining further demonstrated a substantial decrease in phosphomannan levels in the mutant. These findings indicate that phosphate uptake is required to maintain normal phosphomannan content and cell wall architecture (new **Fig. 1c** and new **Supplementary Fig. 1b**).

These findings are consistent with the reviewer's suggestion that phosphate limitation may lead to redistribution of mannose residues across different glycan environments. Although our experiments do not directly determine the enzymatic activity of BMT3/BMT4 or the precise glycan acceptors involved, the observed reduction in phosphomannan staining together with the marked simplification of α-1,2-mannose structural diversity supports substantial remodeling of phosphomannan-associated sidechains or mannoprotein linked glycans. In addition, because GPI-anchored cell wall proteins contribute significantly to the outer mannan layer, impaired phosphate uptake could plausibly influence GPI-anchor maturation or downstream mannoprotein assembly, thereby contributing to the altered fibrillar wall organization.

The combined TEM, alcian blue, and ssNMR analyses support a model in which phosphate transport deficiency does not simply alter total wall composition, but specifically remodels the molecular architecture and structural heterogeneity of the outer mannan-rich cell wall layer. This integrated dataset therefore provides complementary evidence linking phosphate homeostasis to phosphomannan organization and cell wall ultrastructure in *C. albicans*.

We have now added a new paragraph in the maintext, which reads: "To define how phosphate acquisition influences cell wall architecture in *C. albicans*, we combined ultrastructural, biochemical, and solid-state NMR analyses of wildtype and phosphate transport mutant cells. During phosphate starvation, wildtype and mutant cells displayed no substantial differences in overall morphology (**Supplementary Fig. 1**). However, transmission electron microscopy (TEM) revealed a marked reduction in cell wall thickness from 138±18 nm in wildtype cells to 93±11 nm in the mutant, indicating that phosphate uptake is required to maintain normal cell wall structure and integrity (**Fig. 1a, b**). Consistent with this structural change, independent quantification of cell wall phosphomannan by Alcian blue staining showed a dramatic reduction in phosphomannan content in the phosphate transport mutant (**Fig. 1c**), revealing extensive remodeling of the mannan layer."

In addition, we have also added the experimental details in the Methods section regarding these new datasets.

For the N-glycans that constitute mannan the conventional model is an alpha-1,6-linked backbone with alpha-1,2- or -1,3-linked sidechains. Your description (line 194, Line 206/207) would contradict this you state there is an α1,2-linked backbone. This also contradicts your previous paper (https://doi.org/10.1038/s41467-025-61678-1).

Response: Thank you for catching this mistake. We appreciate the reviewer pointing it out. We agree that the conventional structure of *C. albicans* N-linked mannan consists of an α-1,6-linked backbone with α-1,2- or α-1,3-linked sidechains. We have corrected it throughout the maintext.

Figure 5, is quite hard to follow. Why is the chitin not cross-linked to beta-1,3-glucan in the figure?

Response: We have now revised the schematic so that chitin microfibrils in the inner wall domain are shown cross-linked to β-1,3-glucan in both the wild-type and mutant cells. Meanwhile, we added a clarification to the caption of Fig. 5, which reads: "The schematic illustrates polysaccharide organization and redistribution within the cell wall and does not represent the number or extent of covalent cross-links between chitin and glucans."

What is the evidence that chitin synthesis in the mutant results in deposition in the outer layer of the wall? Do you have a model of how chitin which is synthesize by membrane proteins, normally localised close to the plasma membrane would get localised to the outer wall?

Response: We thank the reviewer for this thoughtful question. Chitin is synthesized by chitin synthases at the plasma membrane and is therefore initially deposited within the inner rigid cell wall layer adjacent to the membrane. Consequently, the chitin-associated signals observed in the outer mobile region cannot reflect synthesis at the outer cell surface, but instead suggest redistribution and remodeling within the wall architecture during maturation, a phenomenon previously described by Dr. Neil Gow and colleagues, *e.g.*, Gow, N. A. & Lenardon, M. D., *Nat. Rev. Microbiol.* 21, 248-259 (2023). Our data suggest that in the mutant, chitin is no longer restricted to the inner rigid scaffold and instead becomes more closely associated with mannan-rich domains that normally define the outer mobile wall. In wildtype cells, mannan sidechains form a flexible outer layer that is relatively separated from the inner β-glucan-chitin scaffold. In contrast, the mutant exhibits increased rigid mannan signals together with elevated chitin content in the rigid fraction. At the same time, the mobile mannan sidechains become less structurally diverse, suggesting reduced conformational flexibility, and instead, increased association with rigid wall components.

These observations suggest a possible model in which chitin remains membrane-proximal at the time of synthesis but may become progressively incorporated into reorganized wall regions during cell wall maturation. In the mutant, altered wall packing and reduced flexibility of the mannan layer likely promote stronger interactions between chitin fibrils and mannan-rich domains, resulting in the incorporation of chitin into more external regions of the wall without changing its site of synthesis.

We have added new sentences in the Discussion, which reads "In the mutant, some chitin microfibrils appear to reorganize into the outer wall domain, where they contact and rigidify mannan fibrils, similar to the remodeling mechanism observed in *C. albicans* following echinocandin treatment. Although chitin synthesis occurs at the plasma membrane, the reorganized wall architecture in the mutant likely promotes progressive redistribution of chitin into mannan-associated outer wall regions during maturation."

Chitin is usually cross-linked to beta 1,3 glucan and in past cell wall stress studies has reported changes in the number of cross-links, this is reinforced by upregulation of carbohydrate active enzymes under those conditions. And under cell wall stress it has been suggested there are more bonds between chitin and beta 1,6 glucan, any evidence for this?

Response: We do not have direct evidence for this. To avoid confusion, we added a sentence to the caption of Fig. 5 clarifying that the schematic does not represent the number or extent of covalent cross-links between chitin and glucans. Although we could not directly quantify specific covalent cross-links, solid-state NMR data support substantial polysaccharide reorganization under oxidative stress. In the wild type, β-1,6-glucan and α-1,2-mannose redistribute from highly mobile to more rigid fractions, while β-1,3-glucan and chitin remain prominent in the rigid core, consistent with tighter packing and enhanced physical association within the inner wall scaffold. This redistribution is less pronounced in the phosphate transport mutant (**Fig. 3**).

Responses to Reviewers' Comments

Reviewer 1

I generally appreciate the authors comprehensive rebuttal - but I would feel that some of the conclusions made by the authors are not yet solid enough to judge without some form of statistics.

In particular, I appreciate that the authors provided cross-sections of the 2D CC spectra (new Supplementary Figure 11). I have also analyzed the raw data (thank you also for providing these data!). Notwithstanding, the - apparent – LW differences between WT and mutant are sometimes less than 20 Hz. This does not seem convincing without some sort of statistics – NMR spectroscopist have observed such LW variations between different sample preparations.

I hence like to ask the authors the following: Please list, in a table format, a LW comparison for all resolved cross-peaks of α-1,2-mannose and β-1,3-glucan, and lease confirm these LW differences with a 2nd set of biologically independent samples. If this article got accepted, please also make the raw NMR data available.

Response: We thank the reviewer for the guide. We prepared and analyzed a second biologically independent sample replicate and compiled FWHM values for all resolved α-1,2-mannose and β-1,3-glucan cross-peaks from 2D ^{13}C-^{13}C CORD spectra and added as a new table (**Supplementary Table 6**). Newly included 1D cross-sectional analysis of batch 2 (**Supplementary Figs. 12** and **13**) also confirmed consistent H_2O_2-induced linewidth broadening patterns across both batches.

Wildtype cells consistently demonstrated substantially greater H_2O_2 induced linewidth broadening than the phosphate transport mutant across all resolved α-1,2-mannose resonances (e.g., Batch 1 ΔLW = 284.5 Hz vs 138.5 Hz at 101.5 ppm; Batch 2: ΔLW = 192.6 Hz vs 84.4 Hz; which is approximately 2-fold difference; ΔLW = difference of FWHM of control LW and H_2O_2 treated LW), confirming robust oxidative perturbation of the rigid polysaccharide layer in wildtype cell wall. In contrast, β-1,3-glucan resonances showed minimal H_2O_2 induced linewidth broadening in both strains (Batch 1 at 103.8 ppm: WT ΔLW = 89.2 Hz vs mutant ΔLW = 7.5 Hz; Batch 2: WT ΔLW = 21.5 Hz vs mutant ΔLW = 14.7 Hz), indicating that the more rigid core polysaccharide (β-1,3-glucan) are substantially protected from peroxide induced damage in both wildtype and mutant cell wall. This differential susceptibility where α-1,2-mannose exhibit extensive H_2O_2 induced perturbation while β-1,3-glucan remains relatively resistant demonstrates that the rigid core's structural integrity is preserved or undergoes minimal perturbation under oxidative stress.

All raw ssNMR datasets, including spectra from both biological replicates, have now been deposited to Zenodo with the DOI provided in the data availability section.

Reviewer 3

I would like to thank the reviewers for modifying the manuscript according to reviewers suggestions. I believe the new data/figures such as the images of cell morphology and the TEM to measure cell wall thickness have strengthened the manuscript. I am satisfied that all points have been adequately addressed.

Response: We sincerely appreciate the reviewer's positive assessment.

Reviewer #1 (Remarks to the Author):

I thank the authors for their rigorous data analysis and for providing the raw data. For me this manuscript could be advanced to publication stage. Congratulations to this very meaningful work.

Markus Weingarth.

Response: We sincerely appreciate the reviewer's valuable guidance, which significantly contributed to the improvement of our manuscript during the previous revision. We are grateful for the reviewer's support for its publication.
